# Neural Correlates of Non-ordinary States of Consciousness in Pranayama Practitioners: The Role of Slow Nasal Breathing

**DOI:** 10.3389/fnsys.2022.803904

**Published:** 2022-03-21

**Authors:** Andrea Zaccaro, Andrea Piarulli, Lorenza Melosini, Danilo Menicucci, Angelo Gemignani

**Affiliations:** ^1^Department of Surgical, Medical and Molecular Pathology and Critical Care Medicine, University of Pisa, Pisa, Italy; ^2^Department of Neuroscience, Imaging and Clinical Sciences, “G. d’Annunzio” University of Chieti-Pescara, Chieti, Italy; ^3^Giga Consciousness, Coma Science Group, University of Liège, Liège, Belgium; ^4^Pneumology Branch, Azienda Ospedaliero Universitaria Pisana, Pisa, Italy; ^5^Clinical Psychology Branch, Azienda Ospedaliero Universitaria Pisana, Pisa, Italy

**Keywords:** EEG, altered consciousness, small worldness, olfactory epithelium, slow nasal breathing, respiration, cortical activity modulation

## Abstract

The modulatory effect of nasal respiration on integrative brain functions and hence consciousness has recently been unambiguously demonstrated. This effect is sustained by the olfactory epithelium mechanical sensitivity complemented by the existence of massive projections between the olfactory bulb and the prefrontal cortex. However, studies on slow nasal breathing (SNB) in the context of contemplative practices have sustained the fundamental role of respiratory vagal stimulation, with little attention to the contribution of the olfactory epithelium mechanical stimulation. This study aims at disentangling the effects of olfactory epithelium stimulation (proper of nasal breathing) from those related to respiratory vagal stimulation (common to slow nasal and mouth breathing). We investigated the psychophysiological (cardio-respiratory and electroencephalographic parameters) and phenomenological (perceived state of consciousness) aftereffects of SNB (epithelium mechanical – 2.5 breaths/min) in 12 experienced meditators. We compared the nasal breathing aftereffects with those observed after a session of mouth breathing at the same respiratory rate and with those related to a resting state condition. SNB induced (1) slowing of electroencephalography (EEG) activities (delta-theta bands) in prefrontal regions, (2) a widespread increase of theta and high-beta connectivity complemented by an increase of phase-amplitude coupling between the two bands in prefrontal and posterior regions belonging to the Default Mode Network, (3) an increase of high-beta networks small-worldness. (4) a higher perception of being in a non-ordinary state of consciousness. The emerging scenario strongly suggests that the effects of SNB, beyond the relative contribution of vagal stimulation, are mainly ascribable to olfactory epithelium stimulation. In conclusion, slow Pranayama breathing modulates brain activity and hence subjective experience up to the point of inducing a non-ordinary state of consciousness.

## Introduction

Spontaneous breathing is driven and shaped by interactions between neural circuits within the brainstem (Smith et al., 2009). However, cognitive and emotional demands deeply modulate its depth and pacing (Evans et al., 2009). In recent years, a series of studies both in the animal model and in humans has unveiled a fundamental role of nasal breathing in promoting changes of brain activity (Fontanini and Bower, 2006) and coordinating neural oscillations in large cortical and sub-cortical territories (Heck et al., 2017; Tort et al., 2018); indeed, nasal respiration can modulate sensorimotor, cognitive, and emotional processes (Zelano et al., 2016; Heck et al., 2017), as well as the neural correlates of consciousness (Piarulli et al., 2018).

The role of slow breathing in, in the context of meditative practices (Nash et al., 2013, 2019), assumes, therefore, a special psychophysiological interest. Slow breathing is at the basis of the contemplative eastern traditions of voluntary control of breathing, “Pranayama,” which favors the achievement of non-ordinary states of consciousness and self-awareness proper of deeper meditative states. However, both the psychophysiological effects of slow breathing and the neurophysiological mechanisms underpinning its ability to modulate brain processes and consciousness (Piarulli et al., 2018) remain to be fully elucidated.

We have recently highlighted, in a systematic review on slow breathing techniques (Zaccaro et al., 2018), that slow nasal breathing (SNB) induces an improvement of psychological well-being (increased relaxation and reduced anxious and depressive symptoms), sustained by a parasympathetic dominance.

These findings have led some authors to hypothesize a role of respiratory-related vagal stimulation (bottom–up hypothesis, Brown et al., 2013; Gerritsen and Band, 2018) in the slow breathing psychophysiological effects.

Far from rejecting this hypothesis (Narayanan et al., 2002; Brown et al., 2013; Takahashi et al., 2020), we believe that the model lacks its top–down counterpart represented by the respiratory mechanical stimulation of the nasal vault’s olfactory epithelium. The non-olfactory function of the olfactory system was convincingly elucidated by Fontanini and Bower (2006), who underlined the synchronizing effect of odorless air puffs artificially delivered to the olfactory epithelium, on the olfactory bulb and piriform cortex neural activity.

Recent findings have confirmed and extended the hypothesis of Fontanini and Bower (2006); studies on the animal model have shown that nasal breathing entrains and modulates oscillations in vast brain areas, including sub-cortical structures, such as the thalamus, the hippocampus, and the amygdala, together with large cortical territories (Tort et al., 2018). Analogous findings were described in humans; Zelano et al. (2016), studying intracranial electroencephalography (EEG) in epileptic patients, found that spontaneous nasal breathing synchronizes electrical activity in the piriform cortex, as well as in “limbic” areas and that the synchronization was abolished when shifting from nose to mouth breathing. Herrero et al. (2018) further demonstrated that respiratory-driven oscillations spread over several subcortical and cortical structures (i.e., amygdala, hippocampus, parahippocampal gyrus, insula, precuneus, and orbitofrontal, cingulate, temporal, fusiform, and parietal cortices), proposing that nasal breathing could directly modulate cognitive and emotional processes (Varga and Heck, 2017).

In this context, we have previously studied the psychophysiological aftereffects of odorless air-puffs delivered to the olfactory epithelium at a frequency of.05 Hz, in line with slow Pranayama breathing (Piarulli et al., 2018). We observed that the stimulation induced a non-ordinary state of consciousness in healthy awake humans, sustained by an enhancement of delta and theta band power over widespread cortical areas, including the olfactory system, the orbitofrontal cortex, and areas of the default mode network (DMN, Buckner et al., 2008), together with a reversal of the information flow directionality in theta band from the orbitofrontal cortex to the posterior cingulate cortex and precuneus.

We, herein, investigated the role of olfactory epithelium stimulation during slow breathing with the aim of disentangling its effects from those related to respiratory vagal stimulation: nasal breathing activates both the olfactory system by stimulating mechanoceptors within the olfactory epithelium, and the parasympathetic nervous system *via* vagus nerve stimulation, while mouth breathing promotes only the latter mechanism. Thus, the effects of SNB were compared to those induced by a session of slow mouth breathing at the same respiratory rate. Slow nose and slow mouth breathing aftereffects were, in turn, compared to those related to a freely breathing resting state condition.

## Materials and Methods

### Ethics Statement

All the subjects signed written informed consent; the study was approved by the University of Pisa Ethical Committee (AOUP ID 2805) and adhered to the tenets of the Declaration of World Medical Association (2013) and its later amendments.

### Participants

Twelve healthy right-handed volunteers [nine females; age, 48 ± 2 years (mean ± SE)] participated in the study. The eligibility of each volunteer was evaluated by a semi-structured interview conducted by a senior physician and psychiatrist (AG) based on the following criteria:

• no personal or family history of neurological, psychiatric, or somatic disorders
• no chronic or acute condition involving the respiratory tracts
• not having taken any drug acting directly or indirectly on the central nervous system in the previous month

All the subjects had a proven expertise in meditative practices (hours of formal practice, 1,688 ± 177).

### Experimental Protocol

The study consisted of two experimental sessions:

• Slow nasal breathing: the participants were asked to breathe only through the nostrils for 15 min at a respiratory rate of 2.5 breaths per minute, using Samavritti Pranayama technique (Samavritti Pranayama is a squared breathing technique in which subjects focus on respiratory rhythm by mentally counting the duration of each respiratory phase). A respiratory cycle consists of four consecutive phases, each lasting 6 s (i.e., inspiration-pause-expiration-pause; see Supplementary Material S1, SM1 for further details).
• Slow mouth breathing (SMB): the participants were asked to breathe only through the mouth for 15 min at a respiratory rate of 2.5 breaths per minute, following Samavritti Pranayama. In this session, nostrils were kept close using a clinically approved nasal clip.

Each session started with a resting-state phase of 5 min (baseline), followed by 15 min of slow breathing (SNB or SMB) and ended with another 5-min resting-state phase (post). During resting-state phases, the subjects were allowed to breathe spontaneously through their noses.

The two sessions were administered in a randomized order at a time distance of 1 week, one from the other. The order was randomized following a restricted randomization procedure (Lachin et al., 1988); six subjects were submitted first to the SNB and then to the SMB session; the other six first to SMB and then to SNB.

Electrocardiogram (ECG) and respiratory signals were recorded throughout the session; high-density EEG was recorded during baseline and post phases.

Two validated psychometric questionnaires were administered both at the end of the baseline phase and at the end of the session:

• Phenomenology of consciousness inventory [PCI (Pekala, 1991, p. 19)].
• State-trait anxiety inventory Y short form (STAI-6, Marteau and Bekker, 1992; Conti, 1999),

Prior to the beginning of the experimental sessions (10 days before), the volunteers were asked to practice slow mouth breathing one time a day (30 min) for a week to familiarize and get comfortable with the practice.

### Experimental Procedures

All the sessions started at 9:00 a.m. and were conducted in a quiet and darkened room at the Association of Yoga and Natural Therapies’ headquarters (Casciana Terme Lari, Pisa, Italy). After setting-up the physiological recording system (high-density EEG, single-lead ECG, respiratory belt), a 5-min resting state was recorded (baseline); at the end of the resting-state period, PCI and STAI questionnaires were administered. Signals were then recorded for 20 min: 15 min of slow breathing (nose/mouth), and 5 min of resting-state (post phase). The psychometric questionnaires were administered again at the end of the experimental session.

Throughout the recordings, the subjects were lying on a bed in a supine position with their eyes closed. During baseline and post phases, the subjects were asked to breathe spontaneously through their noses, while simply letting the mind wander and avoiding any structured thinking. During slow breathing phases, the subjects had to focalize their top–down attention on the duration of the breathing cycles, mentally counting 6 s for each breathing phase (inspiration-pause-expiration-pause; each breathing cycle lasted thus 24 s, corresponding to a breathing rate of 2.5 breaths per minute).

**FIGURE 1:**
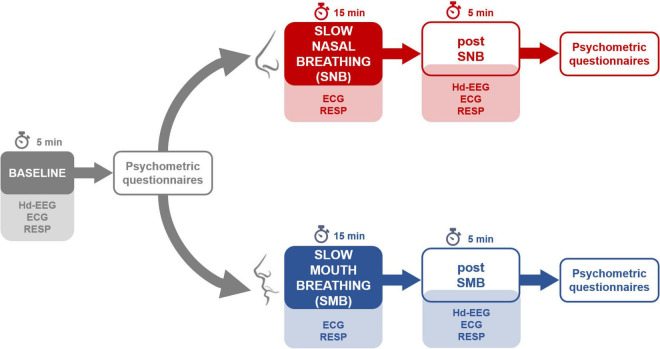
An overview of the experimental procedures for the two sessions. Note that both sessions began with a baseline recording, followed by the administration of psychometric questionnaires.

The blood oxygen level was measured with a clinically validated pulse oximeter (GIMA, Oxy-2, Gima SPA, Milan, Italy) at the end of the slow breathing phase (both for SNB and SMB sessions); blood oxygen saturation was always higher than 98%. An overview of the experimental procedure is presented in Figure 1.

Given the homogeneity of the two baseline phases (SNB and SMB; see Supplementary Material S2), they were collapsed in a single baseline; each physiological and psychometric parameter was thus obtained as the average between SNB and SMB baseline values.

### Polygraphic Signals Acquisition

For each subject and session, polygraphic recordings were carried out using a Net Amps 300 system (GES300, Electrical Geodesic Inc., Eugene, OR, United States), and signals were acquired at a 500 Hz sampling rate using Net Station software (version 4.4.2). EEG was acquired during baselines and post phases, whereas respiratory signals and ECG were recorded also during the slow breathing phase (SNB/SMB). Signal analyses were performed using Matlab framework (MathWorks, Natick, MA, United States).

### Respiratory and Electrocardiogram Signals Processing

Respiratory signals were acquired using a piezo-resistive belt placed on the abdomen (sampling rate, 500 Hz; online band-pass filtering, 0.01–20 Hz). For each subject, session, and phase, the signal was divided in 60-s epochs (50% overlap between consecutive epochs). Each epoch was submitted to a Hanning-windowed Fast Fourier Transform. Mean spectrum density as a function of frequency was obtained for each phase by averaging among epochs pertaining to the phase. The spectrum density peak denoted the dominant breathing frequency (Piarulli et al., 2018).

Cardiac signals were acquired using a single-lead ECG for each subject session and phase. The ECG was filtered (moving average filter) to remove baseline fluctuations, and heartbeat data (QRS complexes) were then extracted from the ECG using the Pan-Tompkins detection algorithm (Pan and Tompkins, 1985). Mis-detected R peaks were identified and corrected using a second algorithm based on point-process modeling (Citi et al., 2012). The obtained tachogram was then processed using Kubios Oy free software (Tarvainen et al., 2014) to extract a series of HRV features (see Supplementary Material S1 for details):

• time domain parameters: HR, SDNN, and RMSSD

• frequency domain parameters: powers in HF (0.15–0.4 Hz), LF (0.04–0.15 Hz), and VLF (0–0.04 Hz) bands, and the LF/HF ratio.

### Electroencephalography Data Collection and Pre-processing

EEG signals were recorded with a 128 electrodes HydroGel Geodesic Sensor Net (Electrical Geodesic Inc., Eugene, OR, United States) at a 500-Hz sampling rate. EEG channels were referenced to the vertex, and electrodes impedance was kept below 50 kΩ. EEG recordings were analyzed using tailored codes written in Matlab (MathWorks, Natick, MA, United States) and EEGLAB toolbox functions (Delorme and Makeig, 2004). Signals were down-sampled to 250 Hz, high-pass filtered at 0.1 Hz (Chebyshev II filter) and notch filtered at 50 Hz and its first harmonic (100 Hz). Channels located on the forehead and cheeks, which mostly contribute to movement-related noise, were discarded, retaining thus 107 channels out of 128 (Piarulli et al., 2018). For each session and phase (baseline and post), epochs with signals exceeding 100 μV were automatically discarded. We confirmed the rejection of these epochs by visual inspection by two EEG experts (AP and DM). Retained epochs were then visually inspected for the removal of artifacts and noisy channels. Rejected signals (ranging from two to seven, depending on the EEG recording) were substituted with signals obtained *via* spline interpolation (Junghofer et al., 2000). Retained epochs of each phase (baseline and post phases) were concatenated and submitted to an independent component analysis to remove ocular and/or muscular artifacts (Delorme and Makeig, 2004). The signals were finally re-referenced to the mastoids’ average (Piarulli et al., 2018).

### Spectral Power and Connectivity Analyses

A spectral analysis was conducted for each subject, session, and phase (post-SNB, post-SMB, and baseline). EEG power was obtained for six frequency bands of interest: delta (1–4 Hz), theta (4–8 Hz), alpha (8–12 Hz), low-beta (12–20 Hz), high-beta (20–30 Hz), and gamma (30–45 Hz). Power densities were estimated, applying a Hanning-windowed FFT on 4-s epochs (50% overlap between consecutive epochs), and log-transformed. For each epoch and electrode, the mean band power was estimated by averaging over its frequency bins. For each phase, the mean power of each band and electrode was then obtained, averaging for each electrode among the band powers of epochs pertaining to the phase and log transformed. At the end of the procedure, we obtained a power scalp map for each subject, session, phase, and frequency band.

For each subject, session and phase, connectivity between each couple of electrodes was estimated using the debiased weighted Phase Lag Index (dwPLI, Vinck et al., 2011). The dwPLI at frequency bins of 0.25 Hz (range, 1–45 Hz) was estimated on 4-s epochs (50% overlap between consecutive epochs). For each epoch and couple of electrodes, the dwPLI in each band of interest was obtained by averaging over its frequency bins. The average dwPLI for each phase, band, and couple of electrodes was finally estimated for each couple of electrodes by averaging over the band dwPLIs of epochs pertaining to the phase. At the end of the procedure, we obtained a dwPLI scalp map for each subject, session, phase, and frequency band.

Finally, for each subject, session, and phase, the dwPLI values across all channel pairs were used to construct symmetric 105-x-105 connectivity matrices for delta, theta, alpha, low-beta and high-beta, and gamma band. Scalp power maps were visualized using EEGLAB functions (Delorme and Makeig, 2004), and connectivity maps were realized using Brain Net toolbox (Xia et al., 2013).

### Phase-Amplitude Coupling

An envelope to signal correlation (ESC, Bruns and Eckhorn, 2004; Onslow et al., 2011) was used to quantify Phase-Amplitude Coupling at each electrode; namely, we estimated the coupling between the time course of the theta band oscillations phase and that of high-beta and gamma bands oscillations amplitudes (as control, ESC was estimated also between delta band and high-beta and gamma bands). The calculation of ESC was based on PAC toolbox functions (Onslow et al., 2011). For each session, phase, couple of bands (e.g., theta-high beta), and electrode, the ESC was estimated on 4-s consecutive epochs; the average ESC at each electrode was then obtained by averaging among epochs.

### Graph Analysis

Connectivity matrices were thresholded, varying the connection density to retain between 90 and 10% of the higher dwPLI values in steps of 2.5% (Chennu et al., 2017). At each connection density, the matrix was represented as a graph, with channels as nodes and non-zero connectivities as edges (links) between the nodes. Each weighted graph was then analyzed by using graph-theoretical algorithms implemented in the brain connectivity toolbox (Rubinov and Sporns, 2010). We characterized each graph using the following metrics:

- *Strength*: the strength of a node is defined as the sum of its edges; the mean graph strength is then estimated as the average over nodal strengths.
- *Clustering coefficient*: it quantifies for each node, the likelihood for two of his neighbors to be connected. The mean clustering coefficient thus reflects the degree of clustered (local) connectivity within a graph.
- *Global efficiency* is a measure of network integration, defined as the average inverse shortest path length.
- *Modular structure and modularity*: The modular structure of a graph is obtained by sub-dividing the network in groups of nodes (maximizing the number of within-group links and minimizing the number of between-group links). Modularity represents the degree of reliability of a given modular structure (Newman, 2004).
- *Participation coefficient*: it represents the degree to which a node within a module is interconnected with other modules. Nodes with a high participation coefficient facilitate the inter-modular integration, and, as such, a network with a high participation coefficient is likely also globally interconnected.

For each subject, session, phase and band, each metric was averaged over the considered connection densities (90–10%, in steps of 2.5%; see Supplementary Material S7: Figures S16–S21).

Graph theoretical metrics allow for a thorough characterization of the topological features of a network, but they do not indicate how the network is embedded in topographical space. We thus used the modular span [first introduced by Chennu et al. (2014)], which estimates the weighted topographical distance spanned by a module, linking a topological construct (i.e., the module) to a topographical measure of its extension on the scalp. Let us consider a graph with a previously identified modular structure; the modular span of the *K^th^* module is defined as:

$$S=\frac{1}{n_{K}}\,\sum_{\left(i,j\in K\right)}d_{i\,j}*w_{i\,j},i\neq j$$

Note that *n*_*k*_ indicates the number of nodes of the *K^th^* module, *d*_*ij*_ represents the normalized Euclidean distance between electrodes *i* and *j*, while *w*_*ij*_ is the weight (dwPLI measure) of the link between the same electrodes (nodes).

For each graph and connection density, only the maximum modular span over the modules was retained (Chennu et al., 2014). For each subject, session, phase, and band, the mean modular span was then obtained by averaging over the connection densities (90–10%, in steps of 2.5%).

### Psychometric Assessment

Two psychometric questionnaires were administered during each session, a first time at the end of the baseline phase and a second time at the end of the session: the Phenomenology of Consciousness Inventory (PCI, Pekala, 1991; Italian validation Pekala et al., 2017), and the State-Trait Anxiety Inventory Y short form (STAI-6, Marteau and Bekker, 1992; Italian validation Conti, 1999).

The PCI is a retrospective self-report questionnaire to be completed in reference to a preceding stimulus condition (within 20 min) that allows phenomenological investigation of the state of consciousness of the subject (Pekala, 1991). It comprises twelve major phenomenological dimensions and fourteen sub-dimensions, for a total of 53 items: altered state (subjective feeling of being in an unusual state of consciousness); altered experience (alterations of some aspects of perception; it is divided into four sub-dimensions – body image: changed perception of the body; time sense: changed perception of time flow; perception: changed perception of the world; meaning: unusual or sacred meaning attributed to the lived experience); volitional control (feeling of control over consciousness contents, e.g., thoughts or images); self-awareness (degree of consciousness of the self); rationality (lucidity and rationality of thoughts); internal dialog (degree of verbal thoughts); positive affect (positive experienced emotions; it is divided into three sub-dimensions: joy, sexual excitement, and love); negative affect (negative experienced emotions; it is divided into three sub-dimensions: anger, fear, and sadness); mental imagery (the quantity and type of eyes-closed imagery; it is divided into two sub-dimensions: imagery amount, and imagery vividness); attention (changes of the attentional level; it is divided into two sub-dimensions – direction of attention: inner or outer directed; absorption: absorption and concentrated into the experience); memory (how much the subjects are able to remember the experience); arousal (feeling of physical and psychological tension). Each item consists of two sentences of opposite meaning, separated by a 7-point Likert scale (from 0 to 6).

The STAI-6 is the short form of the STAI-Y, a widely used self-report questionnaire aimed at identifying and measuring state anxiety (Marteau and Bekker, 1992). State anxiety indicates how much a person perceives anxiety (subjective feeling of tension and worry) “in the moment.” Each item consists of a sentence that must be scored on a 4-point Likert scale.

### Statistical Procedures Overview

The homogeneity of the two baseline phases (nose versus mouth) was verified for each set of data (ECG parameters, respiratory rate, EEG features, and psychometric tests) using paired comparisons: (1) for respiratory-ECG parameters, graph theory metrics, and psychometric tests, putative differences were assessed using permutation tests on paired t-statistics (1,000 randomizations) (Ludbrook and Dudley, 1998); (2) for EEG features involving multiple electrodes (spectral power and phase-amplitude coupling) or couples of electrodes (dwPLI-connectivity); putative differences were assessed using single-threshold permutation tests for the maximum t-statistics [1,000 permutations, Nichols and Holmes, 2001; see Supplementary Material S1]. After these confirmatory analyses, the two baseline phases (nose and mouth) were collapsed in a single baseline; each physiological and psychometric parameter was thus obtained as the average between SNB and SMB baseline values.

Statistical analyses on physiological and psychometric parameters were conducted using a common framework with slight differences depending on the considered dataset. Throughout the manuscript, only *p*-values lower than 0.05 are considered significant.

#### Breathing Rhythms and Electrocardiogram

Each parameter (respiratory rate, and HRV parameters) was first submitted to a repeated measures ANOVA with *phase* as a three-level within factor (baseline, post-SNB, and post-SMB). *Phase* significance was assessed using a permutation test on the F-statistic (Ludbrook and Dudley, 1998) based on 1,000 randomizations. P-values (one for each feature) were then adjusted for multiple testing using Benjamini and Hochberg procedure (FDR, Benjamini and Hochberg, 1995). Here and in the following, the FDR threshold was set at *p* = 0.05. When appropriate, *post hoc* analyses were conducted using permutation tests on paired *t*-statistics (1,000 randomizations, Ludbrook and Dudley, 1998). For each parameter, the obtained *p*-values were then corrected for multiple comparisons using Bonferroni–Holm procedure (Holm, 1979).

For each parameter, (respiratory rate and ECG features) putative differences during slow breathing phases (SNB versus SMB) were then assessed using permutation tests on paired *t*-statistics (1,000 randomizations). *P*-values were again adjusted using Benjamini–Hochberg procedure.

#### Electroencephalography Spectral Power and Phase Amplitude Coupling

For each band (a couple of bands for phase amplitude coupling) and electrode, a repeated measures ANOVA with *phase* as a three-level within factor (baseline, post-SNB, and post-SMB) was conducted. For each band (a couple of bands), *phase* significance at each electrode was assessed using a single threshold permutation test for the maximum F-statistics (1,000 permutations; Nichols and Holmes, 2001). Electrodes showing a significant *phase*-effect were then submitted to *post hoc* analyses; between-*phase* differences were assessed using permutation tests on paired *t*-statistics, and the obtained *p*-values were adjusted using Bonferroni–Holm correction for multiple comparisons (Holm, 1979).

#### Electroencephalography Connectivity

For each band and connectivity (dwPLI between couples of electrodes), a repeated measures ANOVA with *phase* as a three-level within factor (baseline, post-SNB, and post-SMB) was conducted. For each band, the significance of *phase*-effect at each connectivity was assessed using a single threshold permutation test for the maximum *F*-statistics (1,000 permutations). Connectivities showing a significant *phase* effect were then submitted to *post hoc* analyses; between-*phase* differences were assessed using permutation tests on paired *t*-statistics (1,000 permutations), and the obtained *p*-values were corrected using Bonferroni–Holm procedure.

#### Band-Wise Graph Theoretic Analyses

Each graph parameter (graph strength, clustering coefficient, global efficiency, modularity, participation coefficient, and modular span) was submitted to a repeated measures ANOVA with *phase* as a three-level within factor (baseline, post-SNB, and post-SMB). For each band, *phase* significance of each parameter was assessed using a permutation test on the *F*-statistic based on 1,000 randomizations (Ludbrook and Dudley, 1998). *P*-values (one for each parameter) were then adjusted for multiple testing using FDR procedure (Benjamini and Hochberg, 1995). Parameters showing a significant *phase* effect were then submitted to *post hoc* analyses. Between-*phase* differences were assessed using permutation tests on paired *t*-statistics, and the obtained *p*-values were adjusted using Bonferroni–Holm correction for multiple comparisons (Holm, 1979).

#### Psychometric Tests

Phenomenology of consciousness inventory dimensions and sub-dimensions scores, as well as STAI scores, were submitted to a repeated measures ANOVA with *phase* as a three-level within factor (baseline, post-SNB, and post-SMB). *Phase* significance was assessed for each psychometric parameter using a permutation test on the F-statistic based on 1,000 randomizations (Ludbrook and Dudley, 1998). *P*-values (one for each psychometric parameter) were then adjusted for multiple testing using Benjamini–Hochberg correction (Benjamini and Hochberg, 1995). Parameters showing a significant *phase*-effect were then submitted to *post hoc* analyses. Between-*phase* differences were assessed using permutation tests on paired *t*-statistics, and the obtained *p*-values were adjusted using Bonferroni–Holm correction for multiple comparisons (Holm, 1979).

## Results

### Overview

We here describe the psychophysiological after effects of slow nasal breathing (SNB, Samavritti Pranayama – 2.5 breaths/min; see Supplementary Material S1). We compared the effects of SNB with those observed after performing a slow mouth breathing (SMB) session at the same respiratory rate. Psychophysiological and phenomenal after effects of both SNB and SMB were, in turn, compared to those observed in a resting-state condition (see Figure 1 for an overview of the experimental procedures).

Twelve healthy volunteers [9 females; age, 48 ± 2 years (mean ± SE)] with a proven expertise in meditative practices (hours of formal practice, 1,660 ± 177) underwent two experimental sessions administered in a randomized order at 1 week, one from the other, counterbalanced across the participants:

• SNB: the participants had to breathe only through their noses, keeping their mouths closed at a respiratory rate of 2.5 breaths per minute.
• SMB: the participants were asked to breathe through their mouths at a respiratory of 2.5 breaths per minute (nostrils were kept closed using a clinically approved nasal clip).

Each session began with a resting-state phase of 5 min (baseline). At the end of the baseline phase, the phenomenology of consciousness inventory (PCI) (Pekala, 1991; Pekala et al., 2017), and the state-trait inventory Y short form (STAI-6) (Marteau and Bekker, 1992; Conti, 1999), questionnaires were administered. Following the administration, the participants went through 15 min of slow breathing (SNB or SMB, depending on the session). After the slow breathing phase, the participants underwent a second resting-state phase (post, 5 min). Psychometric tests were administered again at the end of this latter phase (see Figure 1 for an outline of the experimental protocol). Respiratory signal and ECG were continuously recorded throughout the session (baseline, slow breathing, and post phase), while high-density EEG was acquired during baseline and post slow-breathing phases.

For each session and phase, we estimated the respiratory rate, the heart rate, and a set of parameters to characterize heart rate variability both in time (RMSSD and SDNN) and frequency domains (HF, LF, LF/HF, and VLF; see Supplementary Material S1 for details).

We then examined EEG activity during baseline and post phases in terms of spectral power, spectral connectivity, cross-frequency coupling, and graph-theoretic network metrics.

For each participant, the baseline resting-state phases of SNB and SMB sessions were collapsed in a single baseline; baseline physiological and psychometric parameters were thus obtained as the average between SNB and SMB baseline values. The homogeneity of SNB and SMB baseline phases in terms of cardio-respiratory parameters, EEG features, and psychometric scores was verified by performing between-phases paired comparisons (see Supplementary Material SS2: Tables S1–S8 and Figures S1–S7).

### Cardio-Respiratory Parameters

We verified whether the participants correctly performed slow breathing (both for SNB and SMB) and whether the two breathing techniques were associated with differences in heart rate and/or heart rate variability. No between-phase difference was found when considering respiratory rate (*t* = −0.40, *p* < 0.79); indeed, respiratory rates of SNB and SMB were respectively 2.47 ± 0.18 and 2.57 ± 0.16 breaths/min, in line with the experimental requirements. No significant difference between SNB and SMB was found also when considering ECG parameters (Supplementary Material S3: Table S9).

We next asked whether slow breathing (either SNB or SMB) induced significant changes in cardio-respiratory parameters during post phases. Again, no significant *phase* effect was found for any parameter (Supplementary Material S3: Table S10) except when considering HRV VLF. This latter parameter showed a significant *phase* effect (*F* = 4.13, *p* < 0.01, non-corrected). However, after FDR correction, the VLF phase effect showed only a tendency toward significance (*p* < 0.07). *Post hoc* analyses (Supplementary Material S3: Table S11) revealed that both post-SNB and post-SMB phases had significantly higher VLF values as compared to the baseline [respectively, *t* = 2.15, *p* < 0.05 and *t* = 3, *p* < 0.003, after Bonferroni–Holm correction (Holm, 1979)]. At variance, no significant difference was observed when comparing post-SNB to post-SMB (*t* = 0.06, *p* < 0.96).

### Slowing of Brain Rhythms After Slow Nasal Breathing

The average power spectral density (PSD) at each EEG channel was estimated for each subject, session, and phase in six bands of interest: delta (1–4 Hz), theta (4–8 Hz), alpha (8–12 Hz), low-beta (12–20 Hz), high-beta (20–30 Hz), and gamma (30–45 Hz). Average PSD scalp maps for each band and phase are presented in Supplementary Material S4: Figure S7. For each band and channel, a repeated-measures ANOVA with *phase* as a three-level within factor (baseline, post-SNB, and post-SMB) was conducted. For each band, *phase* significance at each channel was assessed using a single threshold permutation test for the maximum *F*-statistic (Nichols and Holmes, 2001); see Supplementary Material S4: Figures S8, S9. *Post hoc* analyses were conducted only for those electrodes, showing a significant *phase* effect (Supplementary Material S4: Tables S12, S13).

**FIGURE 2:**
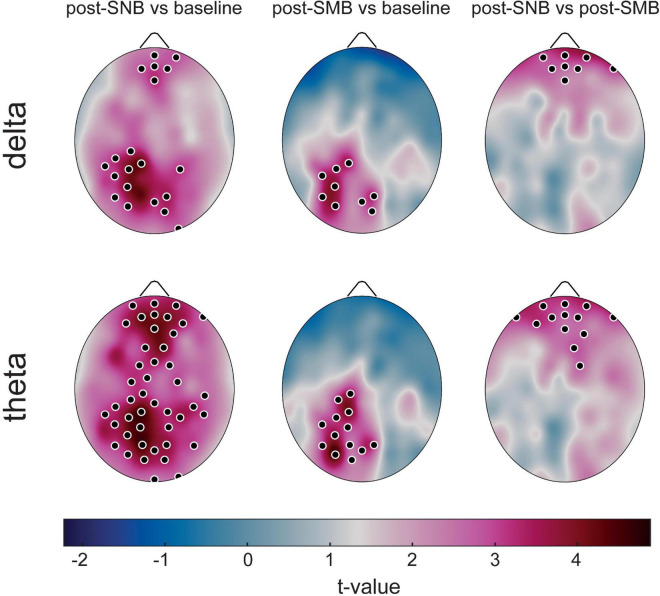
Between-phase power spectral density differences at low frequencies. Scalp maps representing Power Spectral Density between-phase comparisons (*post-hocs*), are presented for delta and theta bands. For each between-phase comparison, the scalp plot denotes the electrode-wise *t*-value distribution related to the band and couple of phases. Electrodes showing a significantly higher value during the first phase, as compared to the second one, are identified by black dots.

We observed an increase of PSD in theta and delta bands after SNB as compared to the baseline (see Figure 2 and Supplementary Material: Tables S12, S13). As apparent from Figure 2, theta significant increases were detected in prefrontal, midline fronto-central, and posterior regions, while, for delta, significant increases involved medial prefrontal areas and posterior regions. Post-SMB, as compared to the baseline, was characterized by PSD increases in medial and left centro-posterior regions both for theta and delta bands.

Finally, a higher PSD in theta and delta bands was observed after SNB as compared to post-SMB in prefrontal and frontal areas (Figure 2). No significant *phase* effect was found either for alpha, low-beta, high-beta, or gamma bands (Supplementary Material S4: Figures S8, S9).

### Heightened Connectivity in Delta, Theta, and High-Beta Bands Following Slow Nasal Breathing

**FIGURE 3:**
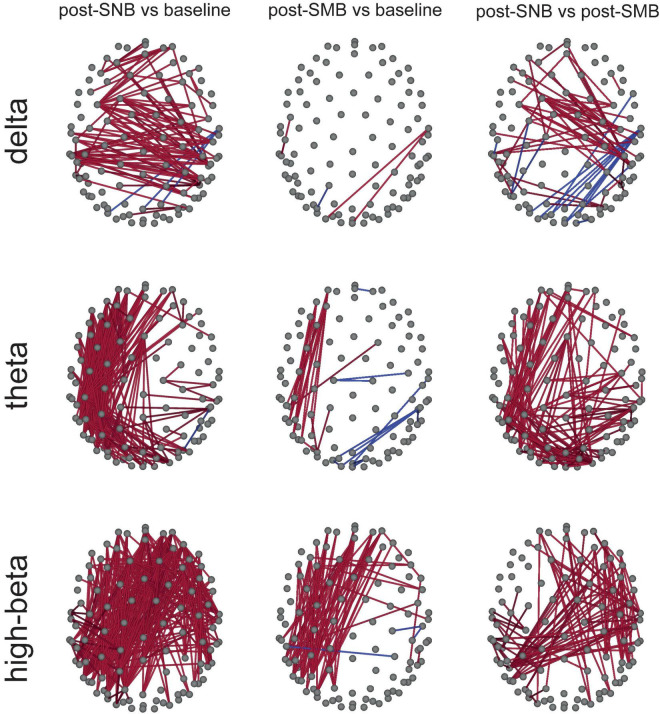
Heightened connectivity in slow- (delta-theta) and high-(high-beta) frequency bands after SNB. Significant connectivity differences between couples of phases (post-SNB, post-SMB, and baseline) are presented for delta, theta, and high-beta bands. Red lines indicate significant comparisons where the former phase value is higher than that related to the latter phase; blue lines indicate the opposite relationship.

Connectivity between all couple of channels was estimated for each subject, session, and phase in the six bands of interest, using the debiased weighted phase lag index (dwPLI, Vinck et al., 2011). For each band and connectivity (between couples of channels), a repeated-measures ANOVA with *phase* as a three-level within factor (baseline, post-SNB, and post-SMB) was conducted. For each band, *phase* significance at each couple of channels (i.e., connectivity) was assessed using a single threshold permutation test for the maximum *F*-statistic (Nichols and Holmes, 2001). *Post hoc* analyses were conducted only for those couples of channels showing a significant *phase* effect (Supplementary Material S5: Figures S10, S11). Relevant connectivity differences between post-SNB, post-SMB, and baseline phases were found for delta, theta, and high-beta bands (Figure 3).

We observed an increase of delta connectivity during post-SNB as compared to the baseline involving frontal, central, and temporo-posterior regions. Post-SNB showed a higher connectivity mostly involving central regions as compared to post-SMB (Figure 3, third column).

When considering theta band, we found a higher connectivity during the post-SNB phase both as compared to the baseline and post-SMB. The connectivity increase was mostly lateralized, involving the whole left hemisphere (from prefrontal to occipital regions). An increase of connectivity between left frontal and left posterior-temporal clusters of electrodes was found when comparing post-SMB to the baseline (Figure 3, second column).

We next considered high-beta band; we observed a massive and widespread increase of connectivity during post-SNB as compared to the baseline. When comparing post-SNB to post-SMB, we found a higher connectivity between right central, posterior, temporal, and frontal regions and between right posterior-temporal regions and regions of the left hemisphere (including fronto-central, temporal, and posterior areas, Figure 3, third column).

When considering alpha, low-beta, and gamma bands, we found few and scattered significant differences (see Supplementary Material S5: Figure S12). Complete tables of *post hoc* statistics for each band and comparison can be found in Supplementary Material S5: Supplementary Tables S14–S19.

### Increased Theta-High-Beta Coupling After Slow Nasal Breathing

We next asked whether SNB could induce changes in the coupling between low and high frequency oscillations. An envelope to signal correlation (ESC) (Bruns and Eckhorn, 2004; Onslow et al., 2011) was used to quantify the phase-amplitude coupling between oscillations in delta and theta bands, and in high-beta and gamma bands, at each electrode for each session and phase. Average ESC scalp maps for each band and phase are presented in Supplementary Material S6: Figure S13. For each couple of bands (i.e., theta-high-beta) and an electrode, a repeated measures ANOVA with *phase* as a three-level within factor (baseline, post-SNB, and post-SMB) was conducted (Supplementary Material S6: Figures S14, S15). For each couple of bands, electrodes showing a significant *phase* effect (Supplementary Material S6: Figures S14, S15) were then submitted to *post hoc* analyses (Supplementary Material S6: Tables S20, S21).

**FIGURE 4:**
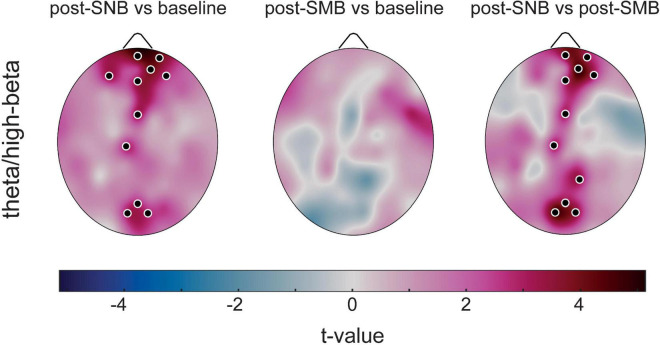
Phase-amplitude coupling (theta/high-beta) increases in DMN regions after SNB. Electrode-wise between-phase differences are presented for each between-phase comparison (*t*-values maps). Significant comparisons (former phase > latter phase) are denoted by black dots.

We observed an overall increase of theta-high-beta coupling after SNB both as compared to the baseline and to post-SMB. Significant increases were found in midline prefrontal/frontal areas and in midline posterior regions with the theta phase modulating high-beta amplitude. At variance, no significant difference was found when comparing post-SMB to the baseline (Figure 4). No significant phase effect was found either for theta-gamma, delta-high-beta, or delta-gamma coupling (Supplementary Material S6: Figures S14, S15).

### Graph Theoretical Metrics

For each subject, session, phase, and band of interest, we estimated six graph theoretical metrics to characterize the network’s key topological features: (1) graph strength, (2) clustering coefficient, (3) global efficiency, (4) modularity, (5) participation coefficient, and (6) modular span.

Each graph parameter was submitted to a repeated measures ANOVA with *phase* as a three-level within factor (baseline, post-SNB, post-SMB). For each band, the *phase* significance of each metric was assessed using a permutation test on the *F*-statistic, and *p*-values (one for each parameter) were adjusted using the FDR procedure (Benjamini and Hochberg, 1995; Supplementary Material S7: Tables S22–S27). Parameters showing a significant *phase* effect were then submitted to *post hoc* analyses (Supplementary Material S7: Tables S23, S26).

No significant *phase* effect was found for any graph metric when considering delta, alpha, low-beta, or gamma networks (Supplementary Material S7: Tables S22, S24, S25, and S27). Graph metrics as a function of connection density are presented for each band in Supplementary Material S7: Figures S16–S21.

#### Theta Networks

**FIGURE 5:**
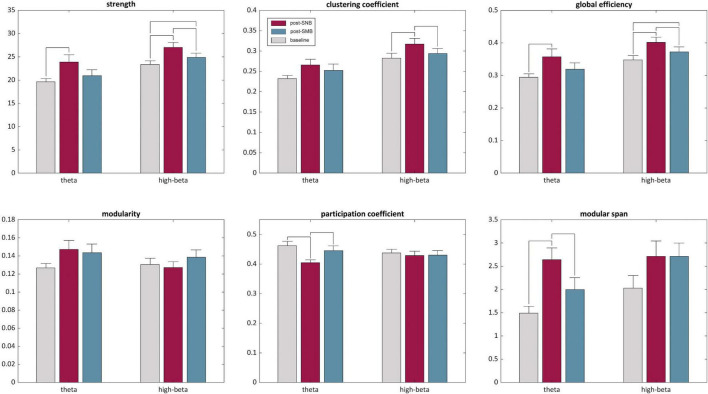
Graph theoretic metrics for theta and high-beta networks. Descriptive statistics of theta and high-beta bands are presented for the three phases (baseline, post-SNB, and post-SMB) as mean + standard error for the estimated graph metrics. For each metric and band, significant *post hoc* (i.e., between phases comparisons) are highlighted by horizontal lines, connecting the corresponding bars.

The post-SNB phase showed higher network strength, clustering coefficient, global efficiency, modularity and modular span, and a lower participation coefficient as compared both to the baseline and post-SMB (see Figure 5). Graph strength, global efficiency, participation coefficient, and modular span showed a significant *phase* effect, while no significant effect was found either for clustering coefficient or modularity (Supplementary Material S7: Table S23a). Statistics related to *post hoc* analyses are detailed in Supplementary Material S7: Table S23b. The post-SNB phase was characterized by higher network strength (*t* = 3.45, *p* < 0.02) and global efficiency (*t* = 3.20, *p* < 0.02) as compared to the baseline. Post-SNB network strength and global efficiency were also higher than those of post-SMB, although differences were not significant.

Post-SNB had a lower participation coefficient both as compared to the baseline (*t* = −3.65, *p* < 0.02) and to post-SMB (*t* = −2.40, *p* < 0.05), suggesting a higher segregation of modules during the post-SNB phase. However, the modular span of the largest module was significantly higher for post-SNB both as compared to the baseline (*t* = 4.18, *p* < 0.003) and to post-SMB (*t* = 2.74, *p* < 0.02).

#### High-Beta Networks

The post-SNB phase showed higher network strength, clustering coefficient, and global efficiency both as compared to the baseline and post-SMB (see Figure 5); we observed a significant *phase* effect for graph strength, clustering coefficient, global efficiency, and modular span, but not for modularity or participation coefficient (Supplementary Material S7: Table S26a). Statistics related to *post hoc* analyses are detailed in Supplementary Material S7: Table S26b. Post-SNB networks had a significantly higher connectivity strength both as compared to post-SMB (*t* = 3.28, *p* < 0.02) and to the baseline (*t* = 4.31, *p* < 0.003). Post-SMB had, in turn, a higher network strength when compared to the baseline (*t* = 2.65, *p* < 0.03).

We found significantly higher levels of clustering (i.e., micro-scale efficiency) during post-SNB both as compared to post-SMB (*t* = 2.77, *p* < 0.03) and to the baseline (*t* = 3.04, *p* < 0.03). Along with the increase of local clustering, post-SNB networks showed higher levels of global efficiency as compared both to post-SMB and to the baseline (*t* = 3.05, *p* < 0.02, and *t* = 4.17, *p* < 0.01, respectively). Finally, post-SMB networks showed a higher global efficiency with respect to the baseline (*t* = 2.41, *p* < 0.03).

### Heightened Perception of Being in an Altered State of Consciousness and Reduction of Anxiety Levels After Slow Nasal Breathing

Each psychometric parameter (PCI dimensions and sub-dimensions as well as STAI scores; see Supplementary Material S8: Table S28 for descriptive statistics) was submitted to a repeated measures ANOVA with *phase* as a three-level within factor (baseline, post-SNB, and post-SMB; see Table 1 and Supplementary Material S8: Table S29). Parameters showing a significant *phase* effect were submitted to *post hoc* analyses (see Table 1 and Supplementary Material S8: Table S30).

**TABLE 1:** Repeated measures ANOVA statistics for PCI scales and sub-scales and STAI-Y scores are reported (phase: baseline, post-SNB, post-SMB, as a three-level within factor).

	RM ANOVA		Post-SNB vs. baseline		Post-SMB vs. baseline		Post-SNB vs. post-SMB	
	*F*	*P*_*FDR*_	*t*	*P*_*BH*_	*t*	*P*_*BH*_	*t*	*P*_*BH*_
**PCI-Positive Affect**	**4.34**	**0.05**	1.45	0.33	–1.53	0.33	**2.97**	**0.04**
Joy	**6.80**	**0.03**	**2.51**	**0.05**	–0.98	0.37	**3.36**	**0.006**
Sex	1.23	0.46	–	–	–	–	–	–
Love	2.53	0.21	–	–	–	–	–	–
**PCI- Negative Affect**	2.00	0.30	–	–	–	–	–	–
Anger	1.65	0.37	–	–	–	–	–	–
Sadness	1.44	0.38	–	–	–	–	–	–
Fear	1.95	0.33	–	–	–	–	–	–
**PCI** – **Altered Experience**	**8.04**	**0.03**	**3.32**	**0.03**	0.53	0.62	**3.00**	**0.03**
Body	**6.53**	**0.04**	**2.54**	**0.05**	–0.48	0.64	**3.28**	**0.02**
Time	**8.59**	**0.03**	**3.40**	**0.02**	**3.64**	**0.02**	1.17	0.25
Perception	2.05	0.33	–	–	–	–	–	–
Meaning	**5.49**	**0.04**	**2.17**	**0.05**	–1.19	0.29	**2.72**	**0.02**
**PCI** – **Visual Imagery**	**5.72**	**0.05**	0.36	0.72	–2.05	0.07	**3.06**	**0.05**
amount	3.18	0.17	–	–	–	–	–	–
vividness	0.97	0.46	–	–	–	–	–	–
**PCI** – **Attention**	1.29	0.42	–	–	–	–	–	–
Inward	1.46	0.38	–	–	–	–	–	–
Absorption	0.27	0.83	–	–	–	–	–	–
**PCI** – **Self Awareness**	0.17	0.87	–	–	–	–	–	–
**PCI** – **AlteredAwareness**	**5.66**	**0.04**	**3.95**	**0.01**	0.30	0.78	**2.40**	**0.05**
**PCI** – **InternalDialogue**	1.62	0.35	–	–	–	–	–	–
**PCI** – **Rationality**	0.11	0.88	–	–	–	–	–	–
**PCI** – **Volition**	0.55	0.67	–	–	–	–	–	–
**PCI** – **Memory**	1.27	0.42	–	–	–	–	–	–
**PCI** – **Arousal**	**4.82**	**0.05**	–1.66	0.32	2.00	0.13	–2.55	0.06
**STAI-Y**	**5.81**	**0.03**	–**2.78**	**0.05**	–0.73	0.51	–**2.63**	**0.05**

*F denotes the statistics of the repeated measures ANOVA (phase effect) and pFDR, the significance after Benjamini–Hochberg correction. Post hoc analyses were conducted only for those psychometric parameters, showing a significant phase effect (pFDR < 0.05). For each psychometric parameter and comparison (i.e., post-SNB vs. baseline, post-SMB vs. baseline, and post-SNB vs. post-SMB), two statistics are presented: t indicates the t-statistics of the paired t-test, and pBH, the test significance after Bonferroni–Holm correction. Everywhere in the table, significant test outcomes (p < 0.05) are written in bold letters.*

Post-SNB was accompanied by an increase of positive experienced emotions (i.e., positive affect), as compared to the baseline (even if the increase was not significant) and to post-SNB (*t* = 2.97, *p* < 0.04). Among positive affect sub-dimensions, we observed a heightened feeling of joy related to post-SNB both as compared to the baseline (*t* = 2.51, *p* < 0.04) and to post-SMB (*t* = 3.36, *p* < 0.01).

The participants reported a heightened perception of being in an altered state of consciousness related to the post-SNB phase; post-SNB (as compared to the baseline and post-SMB) was, in fact, characterized by higher scores both in the altered awareness (*t* = 3.95, *p* < 0.01 and *t* = 2.40, *p* < 0.05) and in the altered experience scale (*t* = 3.32, *p* < 0.02; *t* = 3.00, *p* < 0.02). When considering altered experience sub-dimensions, we observed:

• An alteration in the perception of the body during the post-SNB phase as compared to the baseline (*t* = 2.54, *p* < 0.05) and to post-SMB (*t* = 3.28, *p* < 0.02).

• An unusual meaning attributed to the experience during post-SNB as compared to the baseline (2.17, *p* < 0.05) and to post-SMB (*t* = 2.72, *p* < 0.02).

• An altered perception of the flow of time during post-SNB and post-SMB with respect to the baseline (*t* = 3.40, *p* < 0.02 and *t* = 3.64, *p* < 0.02).

The post-SNB phase was associated with a lower physical and psychological tension as compared to the baseline and post-SMB, although the differences were not significant (arousal levels, *t* = −1.66, *p* < 0.32 and *t* = −2.55, *p* < 0.06, and respectively).

The lower arousal was paralleled by a significant decrease of anxiety levels (STAI), both as compared to the baseline (*t* = −2.78, *p* < 0.04) and to post-SMB (*t* = −2.63, *p* < 0.04); see Table 1 and Supplementary Material S8: Table S30.

## Discussion

Breathing is a fundamental component of many contemplative practices from eastern traditions, which have become popular also in the western culture. Slow paced breathing (e.g., yoga breathing or Pranayama) has been associated with beneficial effects on physical and mental health (Gerritsen and Band, 2018). Despite this broad interest, the psychobiological mechanisms underpinning the efficacy of slow-paced breathing in modulating brain activity, cognition, and, ultimately, consciousness (Piarulli et al., 2018) remain to be fully clarified. Most of the studies dealing with respiration in the context of contemplative practices posit that the sole mechanism by whom breathing enhances psychological well-being and cognitive abilities is related to the activation of the parasympathetic nervous system *via* respiratory stimulation (Brown et al., 2013; Gerritsen and Band, 2018). We do not dispute the contribution of respiratory vagal nerve stimulation, but we believe that the resulting model is incomplete at its best, as it overlooks the contribution of the respiratory-driven activation of the olfactory system in modulating brain activity (Zelano et al., 2016; Heck et al., 2017; Piarulli et al., 2018; Perl et al., 2019).

Here, we investigated the psychophysiological aftereffects of a SNB technique (Samavritti Pranayama at 2.5 breaths/min) in healthy meditative practitioners. The aftereffects of SNB were compared both to those observed after a slow mouth breathing (SMB, same breathing rate, nostrils kept closed using a nasal clip) and to those related to a resting state condition (baseline). The experimental protocol was specifically designed to disentangle the effects of olfactory epithelium stimulation from those related to the vagus nerve, as SNB activates both the olfactory system by stimulating mechanoceptors within the olfactory epithelium, and the parasympathetic nervous system *via* vagus nerve stimulation, while slow mouth breathing promotes only the latter mechanism.

Before proceeding, we believe that some considerations on the use of mouth breathing as a control condition are due; while nasal breathing is innate to human respiration, this is less true when considering mouth breathing, which could theoretically lead to discomfort and cause a different activation of the autonomic nervous system. Having this in mind, we asked the participants to perform at-home training of SMB for a week, starting 10 days before the experiment to gain confidence with the technique (see the “Materials and Methods” section). Moreover, specific subscales of the Phenomenology of Consciousness Inventory Questionnaire (Pekala, 1991), along with self-reports from the participants, seem to exclude any discomfort linked to the SMB practice; indeed, we did not observe any significant change either in arousal levels or negative affect after post-SMB as compared either to post-SNB or baseline phases (Supplementary Material S8: Tables S29, S30).

All the subjects correctly performed both slow nasal and slow mouth breathing sessions (respiratory rates of SNB and SMB were, respectively, 2.47 ± 0.18 and 2.57 ± 0.16 breaths/min). SMB and SNB phases did not differ when considering cardio-respiratory parameters and did not induce significant changes even when comparing the post-slow breathing phases, strongly suggesting that the two techniques have analogous modulatory effects on the autonomic nervous system (i.e., activation of the parasympathetic branch *via* stimulation of the vagus nerve). On the other side, both post-SNB and post-SMB phases showed significantly higher VLF oscillations of HRV as compared to the baseline (respectively, *p* < 0.05 and *p* < 0.003, Supplementary Material S3: Table S11). The identification of the mechanisms underlying VLF is a matter of an ongoing debate, as they were firstly attributed to thermo regulation (Kitney, 1980) and renin-angiotensin-aldosterone system influences (Akselrod et al., 1981). On the other side, Pomeranz et al. (1985) demonstrated that low frequency fluctuations in the HRV (less than.12 Hz, including thus both LF and VLF), in the supine position (analogous to our experimental condition), are mediated by the parasympathetic nervous system.

More recently, several studies (Taylor et al., 1998; Tripathi, 2011; Reyes Del Paso et al., 2013) have confirmed that VLF oscillations are primarily generated by the parasympathetic nervous system.

When considering brain electrical activity (high-density EEG), we observed an increase of slow rhythms (delta and theta bands), both after SNB and after SMB as compared to the baseline phase. During the post-SNB phase, the increase involved prefrontal and centro-posterior areas; that is regions associated with the intrinsic network (Golland et al., 2007) and/or the DMN (Buckner et al., 2008), while the increase observed after SMB was circumscribed to posterior regions. Finally, SNB as compared to SMB induced a significant increase of theta and delta rhythms in medial prefrontal areas.

Present findings about the effects of SNB bring further support to the modulatory role of the olfactory bulb on cortical activity (Fontanini and Bower, 2006; Piarulli et al., 2018) and are consistent with the organization of the olfactory system and its set of anatomical connections; the olfactory bulb has massive projections toward the piriform cortex, which, in turn, projects both directly (Potter and Nauta, 1979; Cinelli et al., 1987) and indirectly (*via* medio-dorsal thalamic nuclei, Ongur and Price, 2000; Courtiol and Wilson, 2015) to the prefrontal cortex. However, these findings only partially replicate previous results from Piarulli et al. (2018), who observed a widespread increase of delta and theta powers over the whole scalp, following a passive mechanical stimulation of the olfactory epithelium. This apparent discrepancy may arise from the differences between the two experimental procedures. In the latter study (Piarulli et al., 2018), the stimulation was specifically conveyed toward the mechanoceptors within the olfactory epithelium to isolate their effects on modulating brain activity, thus ruling out confounding effects related to vagus nerve stimulation.

In the present study, we, instead, investigated the effect of SNB in an ecological setting, possibly modulating its effectiveness in coordinating cortical rhythms over large scalp areas, with two aims:

1) Disentangle modulatory effects of the olfactory system stimulation from those related to the activation of the parasympathetic autonomic nervous system (comparisons between SNB and SMB aftereffects).
2) Verify the vagus nerve stimulation contribution to slow-paced breathing aftereffects (comparisons between slow breathing and the baseline).

Both post-SNB and post-SMB phases showed an enhancement of slow rhythms in posterior regions (as compared to the baseline phase). This enhancement could depend on bottom–up autonomic modulation derived from the slowing of breathing frequency that would elicit vagus nerve activity (Gerritsen and Band, 2018). Indeed, vagal afferents pass through the solitary tract of the brainstem, ending in four main nuclei within the medullary complex, which, in turn, sends direct projections to several structures, including hypothalamus, thalamus, amygdala, insula, and to widespread cortical areas, including parieto-occipital cortices (Chae et al., 2003).

The slowing of cortical rhythms (delta-theta) has been shown to be a hallmark of many non-ordinary states of consciousness; increases of slow activity were observed as an aftereffect of meditation (Lomas et al., 2015; Lee et al., 2018), hypnotic states (Holroyd, 2003), psychedelic drugs administration (Timmermann et al., 2019), and, notably, after passive nostril stimulation at frequencies comparable to those of Samavritti Pranayama (Piarulli et al., 2018). The non-ordinary state of consciousness experienced by the subjects, characterized at a phenomenological level by the perception of an altered experience accompanied by an altered state of awareness, could thus be related to the enhancement of slow activity within key areas of the fronto-parietal network (Piarulli et al., 2018). A plausible hypothesis would be that, during SNB, the thalamus, instead of promoting alpha activity (Hughes and Crunelli, 2005), and the idle states typical of DMN activation (Knyazev et al., 2011), could be driven and paced by the complimentary activity of its afferents:

- The olfactory bulb and piriform cortex, whose activity is paced by SNB (Piarulli et al., 2018).

- The parabrachial area, which receives projections from the nucleus of the tractus solitarius and its vagal afferents, whose activity is promoted by respiratory slow-paced vagal stimulation (Chae et al., 2003; Gerritsen and Band, 2018).

We next observed a widespread increase of connectivity in delta, but, mostly, in theta and high-beta bands after SNB, both as compared to the baseline and to SMB, suggesting that nasal breathing can promote the integration of information over large cortical territories, owing to the increase in long-range connectivity (Zelano et al., 2016). Irrespective of frequency bands and comparisons, significant connections always involved prefrontal regions, bringing further evidence of a prominent role of the olfactory system non-olfactory stimulation in coordinating electrical activity over the cortex. Interestingly, as observed by Gallotto et al. (2017), the emergence of a conscious experience seems to be linked to cortical long-range synchronization in high-beta and gamma bands in line with our findings and with predictions based on the neuronal global workspace theory (Deahene and Naccache, 2001).

When considering phase-amplitude coupling, we found that SNB induced an increase of theta-high-beta coupling, especially when considering midline frontal and posterior regions involved in the DMN, both as compared to the baseline and to post-SMB.

As stated by Canolty and Knight (2010), phase-amplitude coupling provides an effective means to integrate “*fast, spike-based computation and communication with slower external and internal state events guiding perception, cognition, and action*.”

Indeed, slow (theta and delta) oscillations synchronization over large distances may be optimal for promoting interregional (long-range) connectivity (Uhlhaas and Singer, 2010; Hyafil et al., 2015), while faster oscillations (beta-gamma) have been related to increased local synchronization and computation (Sirota et al., 2008; Uhlhaas and Singer, 2010), although long-range synchronization (as observed also in the present study) does occur also at these frequencies (Gross et al., 2004; Uhlhaas et al., 2009).

Theta-high-beta coupling in regions belonging to the DMN, together with heightened long-range connectivity, both at slow and high frequencies, could thus promote two complimentary mechanisms: (1) synchronization and communication between high-beta assemblies across distant areas (Uhlhaas et al., 2009) and (2) local integration of slow and fast activity.

Indeed, cross-frequency coupling has been proposed, along with long-range connectivity, as the core mechanisms at the basis of activity-information retention and local-global information integration (Buzsáki et al., 2013) in a variety of cognitive processes, including working memory tasks, decision-making, and learning (Canolty and Knight, 2010; Hyafil et al., 2015), up to the point of favoring conscious access (Nakatani et al., 2014).

The observed findings, paralleled at a phenomenological level by an increase in positive affect, a reduced anxiety level, and by a change in the state and quality of consciousness, suggest that theta-high-beta coupling increase after SNB may contribute to the modulation of brain functions sustained by fronto-parietal networks (Golland et al., 2007), thus contributing to large-scale integration processes involved in self-awareness (Lou et al., 2017) and consciousness (Varela et al., 2001).

From a purely anatomo-functional standpoint, the increase of positive affect complemented by the reduction of anxiety levels could be explained when looking at the massive bidirectional connectivity between the prefrontal cortex and the amygdala itself, with the densest connections located in the orbitofrontal and medial cortices (Ghashghaei et al., 2007). Moreover, the activity of the amygdala is directly modulated by the olfactory bulb as it receives direct afferent inputs from this latter structure (Carmichael et al., 1994), although a possible role of vagus nerve stimulation cannot be entirely ruled out (Chae et al., 2003).

Current theories on consciousness posit that its emergence critically depends on dynamic balancing between cortical integration and differentiation, sustained by brain networks, enabling an efficient and flexible information transfer (Tononi, 2004; Chennu et al., 2014). In this framework, graph theoretical analysis has gradually become a well-established approach to investigate brain network organization and, hence, the very basis of conscious experience. Indeed, during conscious states, the brain’s functional systems show features typical of complex networks, such as small-world topology, highly connected hubs, and modularity (Bullmore and Sporns, 2009).

Adopting this approach, we observed significant differences in theta but, mostly, in high-beta networks after SNB, both as compared to post-SMB and to baseline phases. When considering the high-beta band, the post-SNB phase was characterized by significantly higher network strength, higher levels of clustering (i.e., micro-scale efficiency), and higher levels of global efficiency, both as compared to the baseline and to post-SMB. These data indicate that high-beta post-SNB networks have stronger small-world attributes (with respect both to the baseline and to post-SMB), as they are simultaneously highly segregated (a higher clustering coefficient) and highly integrated (higher global efficiency). Indeed, during conscious wakefulness, brain networks exhibit optimal organization patterns for information processing and high efficiency of information integration (Achard and Bullmore, 2007), while states of diminished or absent consciousness are characterized by a decrease in brain network integration and an increase in segregation (Bullmore and Sporns, 2009; Chennu et al., 2017). Additionally, disruptions of small-world properties have been found in a variety of pathological conditions, such as major depressive disorders (Ye et al., 2015), schizophrenia (Liu et al., 2008), and multiple sclerosis (He et al., 2009). The higher small-worldness induced by SNB as compared to that observed in ordinary states of consciousness (i.e., “normal wakefulness”) could result in a different functional organization of whole brain neural circuitries.

Our findings about high-beta networks properties gain support from an fMRI study by Gard et al. (2014), who demonstrated that brain functional networks of Yoga practitioners and meditators are characterized by a higher global efficiency and small-worldness as compared to healthy controls.

The small-world architecture (Luppi et al., 2019) seems to be at the very basis of the richness and variety of conscious experience as it is preserved across multiple frequency bands and cognitive demands, while the adaptability to diverse conditions is supported by a dynamic reconfiguration of the set of specific interregional connections that subtend the same global network architecture (Bassett, 2006). The dynamic reconfiguration, along with cross-frequency coupling in key cortical areas, such as prefrontal and posterior cortices, could be at the very basis of the non-ordinary yet full state of consciousness experienced by the volunteers during Pranayama breathing. This hypothesis agrees with the key structure of Tononi’s theory of consciousness (Tononi, 2004) as:

- the emergence of consciousness critically depends on two phenomenological properties (a) the availability of a rich repertoire of different pieces of information (differentiation supported by high local clustering) and (b) the ability of a system/network to integrate the available information (unity-high global efficiency).
- the *quality* of consciousness crucially depends on the informational relationships between the elements of the system/network (i.e., enhanced long-range connectivity both at slow- and high-frequency, theta-high-beta coupling).

This interpretation gains support from phenomenological observations, as post-SNB is characterized by an increase of positive emotions and, specifically, by a heightened feeling of joy (both as compared to the baseline and to post-SMB) and a significant decrease of anxiety. The post-SNB phase is also characterized by a heightened perception of being in a non-ordinary state of consciousness, whose key features are an altered perception of the body, an unusual meaning attributed to the experience accompanied by the perception of being in an altered state of awareness. Notably, and at variance with Piarulli et al. (2018), the non-ordinary state of consciousness here described is not accompanied either by a reduction of rational thinking or volitional control; from a phenomenological perspective, the emerging scenario is that of a deeply relaxed but fully aware non-ordinary state of consciousness comparable to meditation (Nash et al., 2013, 2019).

In conclusion, we found that nasal, and not mouth breathing, is able to induce a non-ordinary state of consciousness characterized at a neurophysiological level by:

1) An enhancement of power at slow frequencies (especially in the theta band) in medial prefrontal and posterior areas
2) A widespread increase of connectivity both at slow (theta) and fast (high-beta) frequencies
3) Heightened theta/high-beta coupling in medial prefrontal and posterior areas

These findings, taken together, suggest that the non-ordinary state of consciousness experienced by the volunteers with its rich phenomenological content seems to be mainly related to the slow-paced stimulation of the olfactory bulb (in line with findings from Piarulli et al., 2018), although we observed a contribution of vagus nerve stimulation when considering power within delta and theta bands in posterior areas of the scalp. Indeed, even if no difference in cardiorespiratory parameters and discomfort was observed when comparing slow nose breathing to slow mouth breathing practices, it might still be possible that vagus nerve stimulation could be more effective during nose breathing than during mouth breathing.

From a neuro-phenomenological standpoint, this study expands the current knowledge about the psychophysiological effects of SNB and on the neural correlates of non-ordinary states of consciousness (Dietrich, 2003; Vaitl et al., 2005).

The presented findings enrich the emerging line of research about high-order functions of respiration in humans, showing that SNB is an effective means for modifying human state and quality of consciousness through the reorganization of neural electrical activity both in the temporal and spatial domains, in line with predictions from the temporo-spatial theory of consciousness (Northoff and Huang, 2017).

Future studies aiming at shedding further light on the relationships between SNB and consciousness, involving a higher number of participants, are warranted; prospective lines of research will include investigations into SNB using combined high-density EEG and functional magnetic resonance imaging, investigations including repeated slow nose breathing sessions with the aim of verifying whether SNB could influence DMN connectivity on a long-term basis, and studies on patients with selective anatomical lesions of the olfactory bulb.

## Data Availability Statement

The raw data supporting the conclusions of this article are available by contacting the authors, without undue reservation.

## Ethics Statement

The studies involving human participants were reviewed and approved by University of Pisa Ethical Committee (AOUP ID 2805). The patients/participants provided their written informed consent to participate in this study.

## Author Contributions

AG and AZ conceived the idea on the basis of the study. AG, AZ, and DM designed and performed the experiments. AP and AZ analyzed the data. AP, AG, AZ, and LM wrote the manuscript. All authors read and approved the final manuscript.

## Conflict of Interest

The authors declare that the research was conducted in the absence of any commercial or financial relationships that could be construed as a potential conflict of interest.

## Publisher’s Note

All claims expressed in this article are solely those of the authors and do not necessarily represent those of their affiliated organizations, or those of the publisher, the editors and the reviewers. Any product that may be evaluated in this article, or claim that may be made by its manufacturer, is not guaranteed or endorsed by the publisher.
