# Supplementary material for: Neural Correlates of Non-ordinary States of Consciousness in Pranayama Practitioners: The Role of Slow Nasal Breathing
Source: Front Syst Neurosci. 2022 Mar 21;16:803904. doi: 10.3389/fnsys.2022.803904 (PMC8977447; doi:10.3389/fnsys.2022.803904)
Supplement: Supplementary file 1 [file Data_Sheet_1.PDF]

SUPPLEMENTARY MATERIAL

SM1

Samavritti pranayama

Slow Samvritti pranayama breathing was performed following the indication published in Zaccaro et al. (2018):

- Breathing was consciously attended and controlled.
- Subjects had to focus on respiratory rhythm by mentally counting the duration of each respiratory phase.
- The mean respiratory frequency was set at 2.5 b/min (0.04 Hz).
- Each breath consisted of four consecutive phases: inspiration-pause-expiration-pause); each phase lasted 6 seconds.
- The inspiration/expiration ratio was consequently equal to 1.
- The breathing was performed using the abdomen (i.e. abdominal breathing).
- No metronome was used.

Zaccaro, A. et al. How breath-control can change your life: a systematic review on psycho-physiological correlates of slow breathing. *Front. Hum. Neurosci.* ***12***, 353 (2018).

ECG features

HR: mean heart rate (*beats/min*).

SDNN: standard deviation of normal-to-normal RR intervals (*ms*, where R is the peak of a QRS complex). It estimates the degree of heart rate variability (HRV) in the time domain.

RMSSD: Square root of the mean squared differences between successive RR intervals (*ms*). It is used to estimate vagally mediated changes in HRV.

VLF: Absolute power of very low frequency band (0-0.04 Hz) (ms^2/Hz). It has been related to vagal tone, thermoregulation, and renin-angiotensin-aldosterone system regulatory functions.

LF: Absolute power of low frequency band (0.04-0.15 Hz) (ms^2/Hz). It reflects baroreceptor-mediated sympathetic and parasympathetic influences on heart rate.

HF: Absolute power of high frequency band (0.15-04 Hz) (ms^2/Hz). It is a marker of parasympathetic activation. It reflects also respiratory influences on the heart rate.

LF/HF: Ratio between LF and HF band powers. It is an estimated of sympatho-vagal balance, and its decrease is a marker of parasympathetic activity prevalence.

Statistical non-Parametric Mapping, SnPM

Single threshold test for the maximum t-statistic

We here briefly introduce the basis and rationale of SnPM: let us assume without loss of generality to have collected an EEG feature for each electrode in two different phases (i.e. SMB and SNB), for a group of subjects. For each electrode, a paired t-test between the conditions is conducted and its t-value is collected. As the test is applied to multiple electrodes (105), a single-threshold SnPM procedure is used to assess the significance of each t-test, tackling with the multiple comparison issue. Let us consider the null-hypothesis of no significant *phase*-effect: under the null-hypothesis, for each subject the labeling of the collected feature can be randomly assigned (i.e. a feature estimated during SMB can be assigned to SNB and vice-versa). Based on this assumption, 1000 random relabeling are made, and for each of them, the t-value related to each single comparison is estimated. For each relabeling, only the maximum t-value (in absolute value, for two-tailed significance assessment) among simultaneous comparisons (i.e. over the electrodes) is retained. At the end of the procedure, the maximum t-value distribution under the null-hypothesis of no significant *phase*-effect is obtained. The significance of each original t-value is then estimated as the ratio between the number of t-values of the null-distribution exceeding the original t-value (in absolute value) and the number of relabeling.

Single threshold test for the maximum F-statistic

Let us now assume to have collected an EEG feature for each electrode (or couples of electrodes, i.e. dwPLI) in three different phases (i.e. post-SNB, post-SMB and baseline), for a group of subjects. For each electrode, a repeated measures ANOVA with *phase* as a within-subject factor is conducted and its F-value is retained. As the test is applied to multiple electrodes (105), a single-threshold SnPM procedure is used to assess the significance of each *phase*-effect (F-value derived from the repeated measures ANOVA), again tackling with the multiple comparison issue. Let us consider the null-hypothesis of no significant *phase*-effect: under the null-hypothesis, for each subject the labeling of the collected feature can be randomly assigned (i.e. a feature estimated during post-SMB can be assigned to baseline, that of baseline phase to post-SNB and the latter to post-SMB or any other possible relabeling). Based on this assumption, 1000 random relabeling are made, and for each of them, the F-value related to each single repeated measures ANOVA is retained. For each relabeling, only the maximum F-value among simultaneous ANOVAs (i.e. over the electrodes) is retained. At the end of the procedure, the maximum F-value distribution under the null-hypothesis of no significant *phase*-effect is obtained. The significance of each original F-value is then estimated as the ratio between the number of F-values of the null-distribution exceeding the original F-value and the number of relabeling.

SM2. Baselines Phases Comparisons

In this section we demonstrate the homgeneity of the two baseline phases (BN, the one of the SNB session and BM the one of the SMB session), in terms of electrophysiological parameters (ECG and EEG) and psychometric scores.

Cardio-respiratory Parameters

	**BN**		**BM**		**BN vs BM**			
	**mean**	**stde**	**mean**	**stde**	**$\|t_{0.05}\|$**	**t**	**p**	**p_{FDR}**
**HR** (hb/min)	63.23	2.44	59.71	1.70	2.20	1.64	< 0.12	< 0.41
**VLF** (ms^2/Hz)	39.75	9.29	50.55	11.77	2.06	-0.71	< 0.51	< 0.59
**LF** (ms^2/Hz)	776.85	321.47	992.00	344.27	2.09	-0.72	< 0.48	< 0.59
**HF** (ms^2/Hz)	449.77	138.48	724.00	244.97	2.02	-1.94	< 0.07	< 0.41
**LF/HF** (au)	2.48	1.06	1.74	0.25	1.81	0.75	< 0.59	< 0.59
**RMSSD** (ms)	31.70	4.92	36.00	7.26	2.05	-1.01	< 0.34	< 0.59
**SDNN** (ms)	33.14	5.06	36.10	6.16	2.14	-0.77	< 0.45	< 0.59

Table S1. Descriptive statistics of BN and BM phases are presented (mean and standard error), along with the statistics of the between-phase comparisons. $|t_{0.05}|$ indicates the two-sided significance threshold (at $p < 0.05$), derived by a permutation test (1000 permutation), on the original dataset, *t*, the t-statistics of the paired t-test, *p* the non-corrected significance of the test (based on the permutation test), and p_{FDR} the significance after adjusting significance values with the Benjamini-Hochberg procedure.

Each parameter was submitted to a paired t-test. The t-value significance was estimated based on 1000 random permutation of the original dataset: under the null hypothesis of no significant session effect, for each subject the value related to BN phase could be assigned to BM and vice-versa. The distribution of t-values under the null-hypothesis was thus obtained by randomly assigning a set of values related to the BN group to the BM group and vice-versa, and at each step (i.e. each permutation), the t-value (absolute value for two-tailed significance), was collected, At the end of the procedure we obtained for each parameter a distribution of 1000 t-values under the null-hypothesis of no significant session effect. The BN-BM comparison significance was thus estimated as the ratio between the t-values of the null-distribution exceeding the real-one (taken in absolute value) and the number of permutations (1000, see Ludbrook and Dudley, 1998). As apparent from Table S1, no significant difference was found between BN and BM, even before applying Benjamini-Hochberg correction for multiple testing.

Power spectral density in the six bands of interest. The procedure for band-wise power spectral density estimation is detailed in the main text (Materials and Methods section).

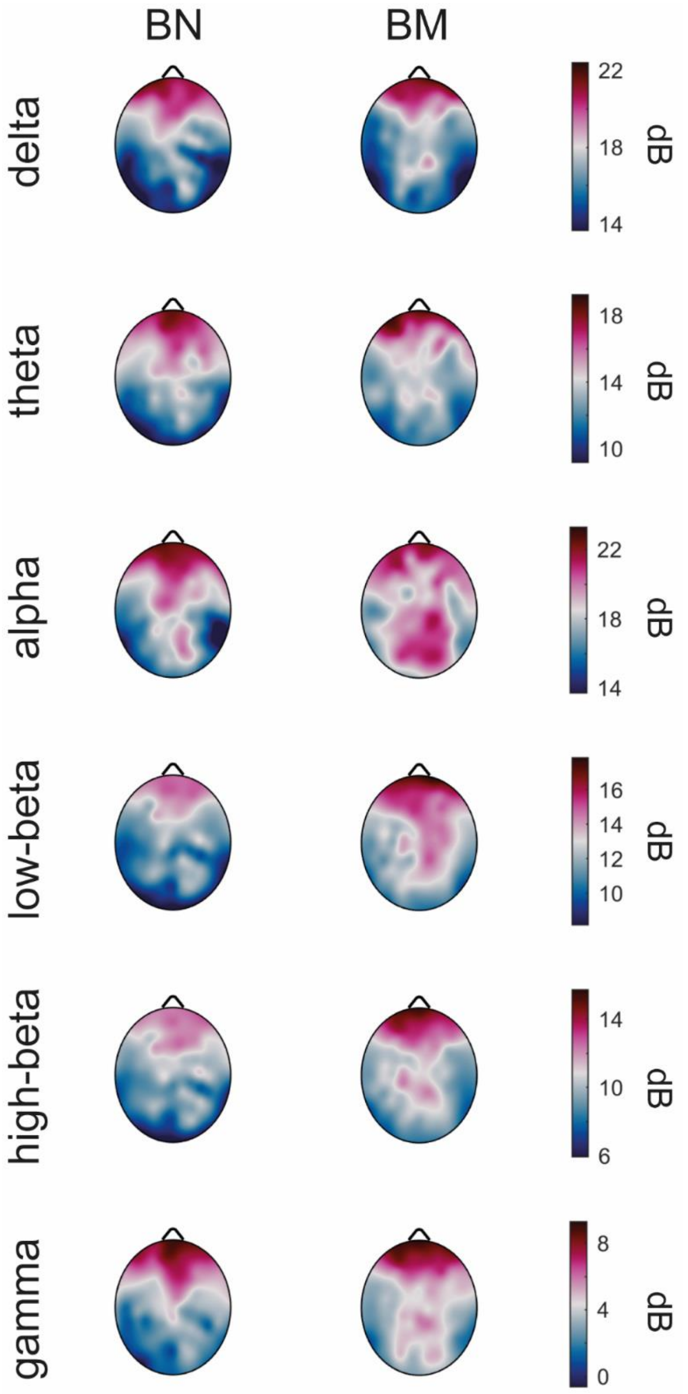

Figure S1. In the present figure, the average spectral density (expressed in dB) is presented for each band and condition (BN and BM). For visual clarity, each band has its own colorbar.

Band-wise PSD comparisons. For each band we performed electrode-wise comparisons between BN and BM. The significance threshold was obtained for each band, using a single threshold permutation test for the maximum t-statistics (Nichols and Holmes, 2001, 1000 permutations).

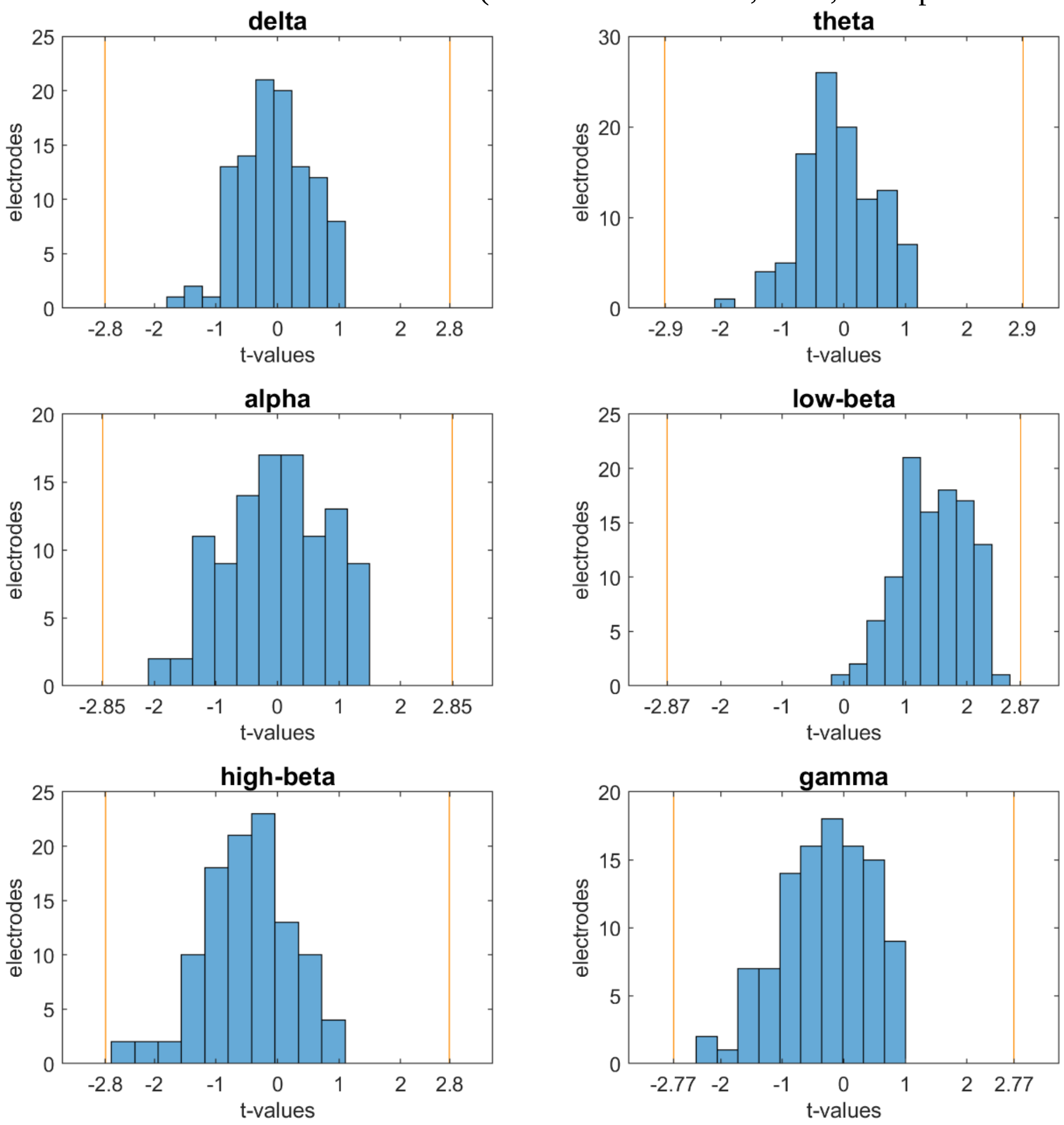

Figure S2. In this figure, the distributions of t-values related to the electrode-wise comparisons are presented for each band (blue bars). In each plot the t-threshold for significance at $p < 0.05$ (estimated using a single threshold test for the maximum statistics) are denoted by orange lines. As apparent from the figure, no significant between-phase difference was found for any band and electrode.

Envelope to signal correlation (ESC, Onslow et al., 2011). We next verified the existence of putative difference in coupling between oscillations in delta and theta bands (modulating signals), and in high-beta and gamma bands (modulated signals).

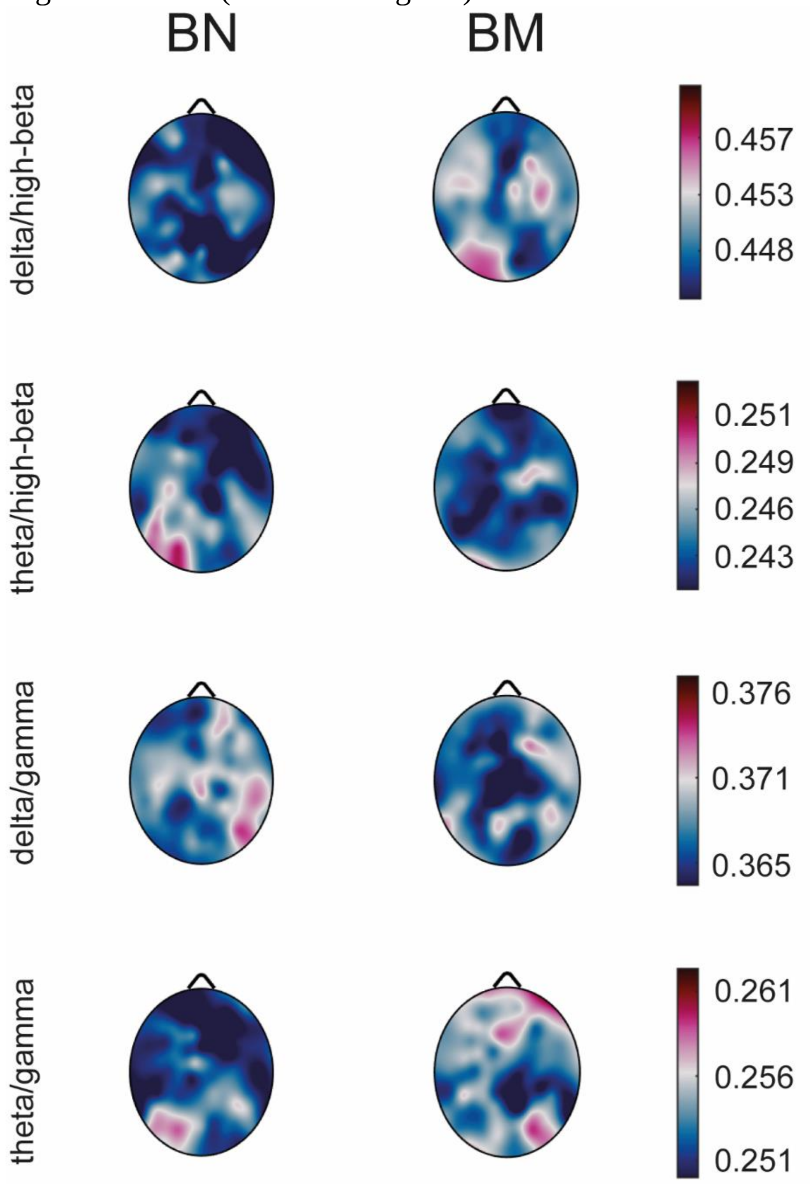

Figure S3. The average coupling over the scalp is depicted for four couple of bands in the two conditions: delta/high-beta, theta/high-beta, delta/gamma and theta/gamma. Note that the coupling can vary between 0 and 1. For visual clarity, each band has its own colorbar.

Couple-wise ESC comparisons. For each couple of bands, electrode-wise comparisons between BN and BM were performed. The significance threshold was obtained for each couple of bands using a single threshold permutation test for the maximum t-statistics (Nichols and Holmes, 2001, 1000 permutations).

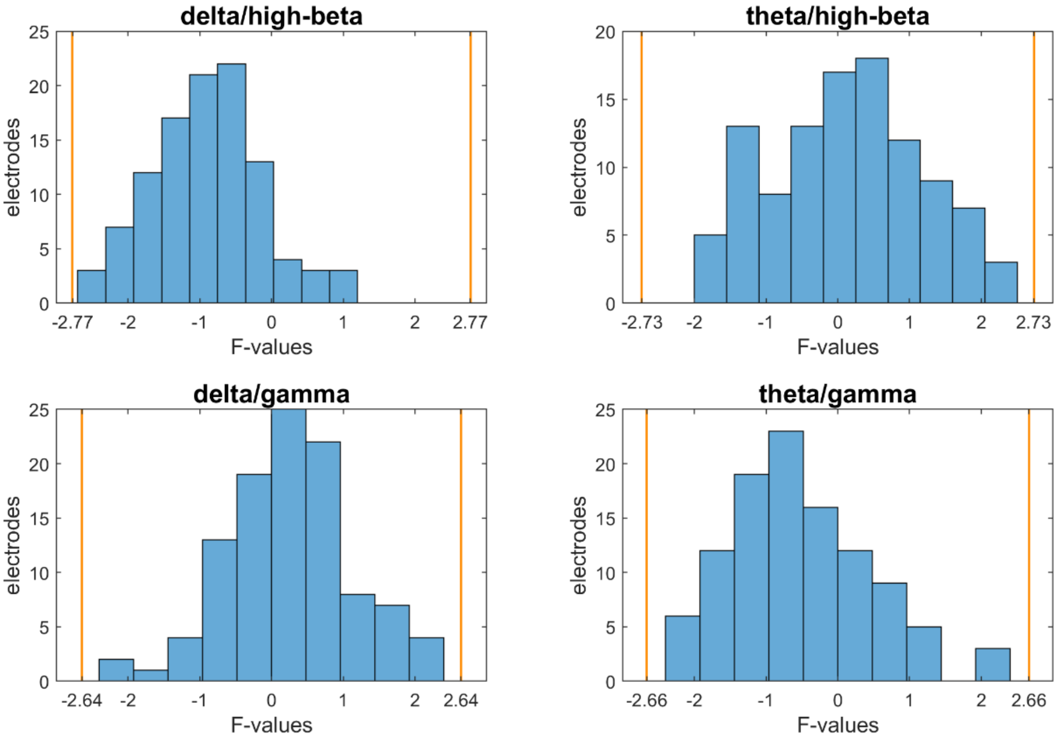

Figure S4. The distributions of t-values related to the electrode-wise comparisons are presented for each couple of bands (blue bars). In each plot the t-thresholds for significance at $p < 0.05$ (estimated using a single threshold test for the maximum statistics) are denoted by orange lines.
As apparent from the figure above, no significant between-phase difference was found for any couple of bands and electrode.

Band-wise connectivity comparisons (debiased Weighted Phase Lag Index, dWPLI).
Connectivity between all couple of channels was estimated for each subject, session and phase in the six bands of interest using the debiased weighted Phase Lag Index (dwPLI, Vinck et al., 2011). For each band and connectivity (between couples of electrodes) comparisons between BN and BM were performed. The significance threshold was estimated for each band, using a single threshold permutation test for the maximum t-statistics (Nichols and Holmes, 2001, 1000 permutations).

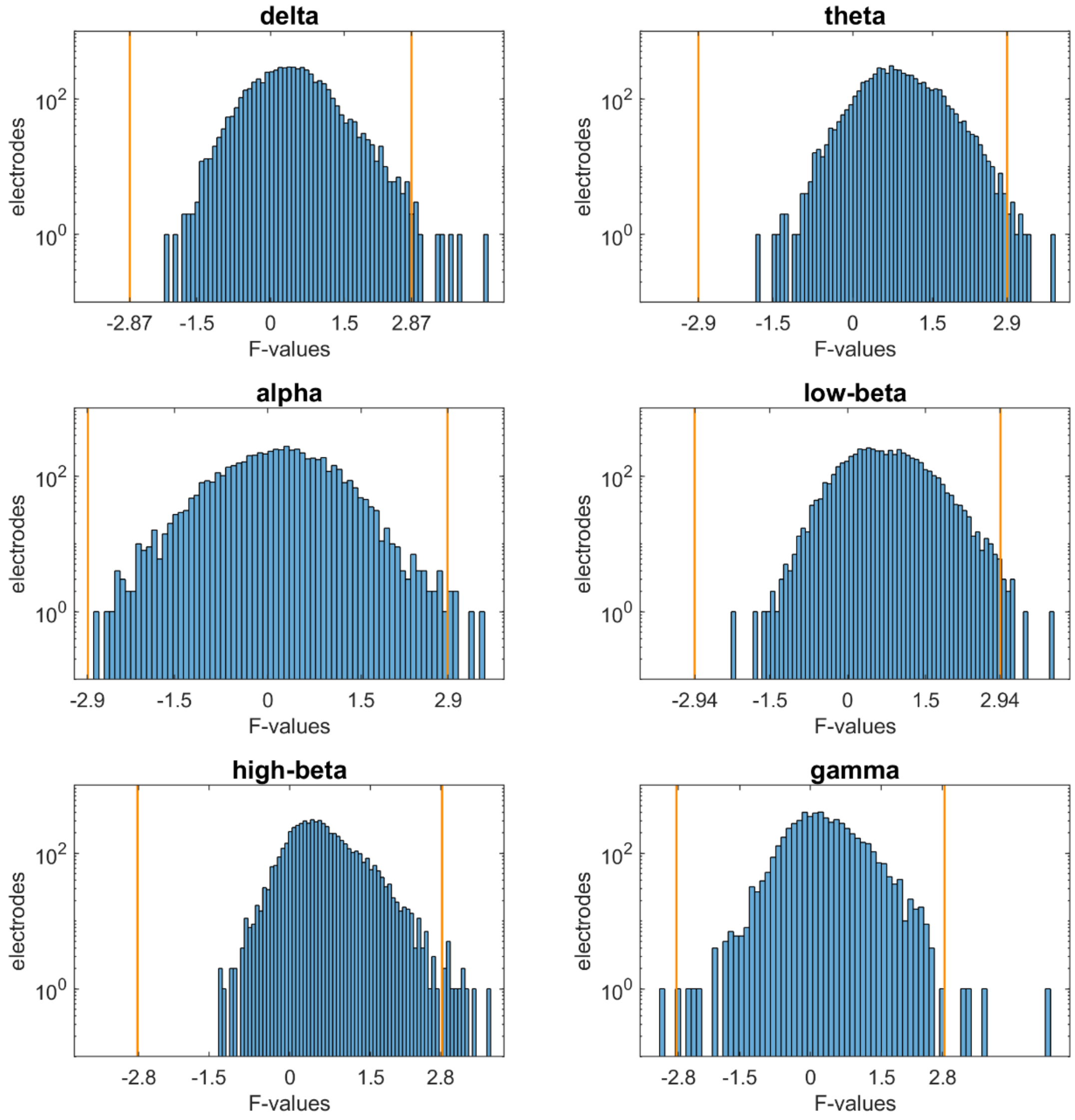

Figure S5. The distributions of t-values related to the connectivity comparisons are presented for each couple of bands (blue bars). In each plot the t-thresholds for significance at $p < 0.05$ (estimated using a single threshold test for the maximum statistics) are denoted by orange lines.

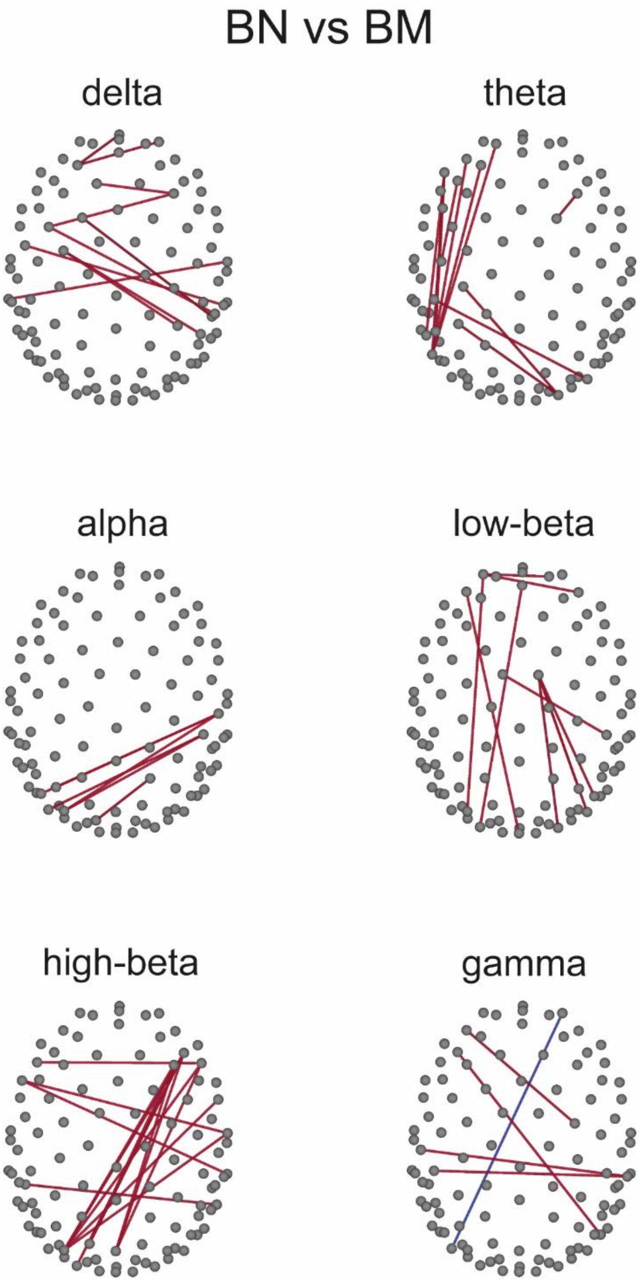

Figure S6. In this figure, significant differences in dWPLI values are presented for each band. Red lines indicate higher connectivity during BN as compared to BM, blue lines higher connectivity during BM.

While we found some difference in connectivity between BN and BM, their number was extremely limited:

- delta 10 out of 5460 couples.
- theta 11 out of 5460 couples.
- alpha 6 out of 5460 couples.
- low-beta 10 out of 5460 couples.
- high-beta 15 out of 5460 couples.
- gamma 5 out of 5460 couples.

Graph Theoretic Metrics

For each subject, session and baseline phase (either related to SNB or SMB sessions), the dwPLI values across all channel pairs were used to construct symmetric 105x105 connectivity matrices for the six bands of interest. Connectivity matrices were then thresholded varying the connection density to retain between 90% and 10% of the higher dwPLI values in steps of 2.5% (Chennu et al., 2017). At each connection density, we estimated six graph theoretical metrics (see Materials and Methods): 1) clustering coefficient, 2) global efficiency, 3) graph strength, 4) modularity, 5) participation coefficient and 6) modular span (Chennu et al., 2014). Graph metrics were subsequently averaged across connection densities (90% to 10 %).

For each band and metric, comparisons between BN and BM were performed using a permutation tests on paired t-statistics (Ludbrook and Dudley, 1998, 1000 permutations), and p-values were adjusted for each band separately, using FDR correction for multiple testing (Benjamini-Hochberg, 1995). As apparent from the tables below, no significant difference between BN and BM was found for any band and graph metric.

**delta metrics**	**BN**		**BM**		**BN vs BM**			
	**mean**	**stde**	**mean**	**stde**	**$\|t_{0.05}\|$**	**t**	**p**	**p_{FDR}**
**clustering coefficient**	0.20	0.01	0.20	0.01	2.27	-0.07	0.95	0.95
**global efficiency**	0.25	0.02	0.28	0.02	2.27	1.90	0.09	0.27
**graph strength**	17.19	1.12	18.04	1.40	2.33	-0.41	0.71	0.85
**Modularity**	0.15	0.01	0.13	0.01	2.15	1.95	0.07	0.27
**participation coefficient**	0.45	0.01	0.47	0.02	2.30	-1.23	0.25	0.50
**modular span**	1.59	0.19	1.43	0.16	2.11	0.67	0.55	0.82

Table S2. Descriptive statistics of BN and BM phases are presented for delta band metrics (mean and standard error), along with the statistics of the between-phase comparisons. $|t_{0.05}|$ indicates the two-sided significance threshold (at $p < 0.05$) derived by the permutation test on t-statistic (1000 permutations), *t*, the t-statistics of the paired t-test, *p* the non-corrected significance of the test (based on the permutations), and p_{FDR} the significance after Benjamini-Hochberg correction.

**theta metrics**	**BN**		**BM**		**BN vs BM**			
	**mean**	**stde**	**mean**	**stde**	**$\|t_{0.05}\|$**	**t**	**p**	**p_{FDR}**
**clustering coefficient**	0.25	0.01	0.23	0.01	2.20	1.63	0.13	0.39
**global efficiency**	0.24	0.01	0.30	0.02	2.23	0.93	0.38	0.39
**graph strength**	21.28	0.62	19.57	1.02	2.13	1.96	0.06	0.38
**Modularity**	0.13	0.01	0.15	0.01	2.16	-0.93	0.39	0.39
**participation coefficient**	0.44	0.01	0.45	0.02	2.23	-1.21	0.26	0.39
**modular span**	1.81	0.19	1.47	0.18	2.27	1.20	0.25	0.39

Table S3. Descriptive statistics of BN and BM phases are presented for theta band metrics (mean and standard error), along with the statistics of the between-phase comparisons. $|t_{0.05}|$ indicates the two-sided significance threshold (at $p < 0.05$) derived by the permutation test on t-statistic (1000 permutations), *t*, the t-statistics of the paired t-test, *p* the non-corrected significance of the test (based on the permutations), and p_{FDR} the significance after Benjamini-Hochberg correction.

alpha metrics	BN		BM		BN vs BM			
	mean	stde	mean	stde	$\|t_{0.05}\|$	t	p	p_{FDR}
**clustering coefficient**	0.41	0.02	0.41	0.02	2.14	-0.02	0.97	0.97
**global efficiency**	0.46	0.03	0.53	0.03	2.26	1.93	0.08	0.49
**graph strength**	34.88	2.41	35.02	1.96	2.19	-0.07	0.95	0.97
**Modularity**	0.12	0.01	0.11	0.01	2.09	0.64	0.53	0.97
**participation coefficient**	0.44	0.01	0.43	0.01	2.33	1.42	0.19	0.57
**modular span**	3.65	0.61	3.48	0.45	2.15	0.30	0.75	0.97

Table S4. Descriptive statistics of BN and BM phases are presented for alpha band metrics (mean and standard error), along with the statistics of the between-phase comparisons. $|t_{0.05}|$ indicates the two-sided significance threshold (at $p < 0.05$) derived by the permutation test (1000 permutations) on t-statistic, *t*, the t-statistics of the paired t-test, *p* the non-corrected significance of the test (based on the permutations), and p_{FDR} the significance after Benjamini-Hochberg correction procedure.

low-beta metrics	BN		BM		BN vs BM			
	mean	stde	mean	stde	$\|t_{0.05}\|$	t	p	p_{FDR}
**clustering coefficient**	0.26	0.01	0.26	0.02	2.10	-0.26	0.80	0.94
**global efficiency**	0.28	0.01	0.34	0.03	2.13	1.18	0.27	0.94
**graph strength**	22.05	1.01	22.15	1.79	2.13	-0.08	0.94	0.94
**Modularity**	0.13	0.01	0.13	0.01	2.25	0.34	0.74	0.94
**participation coefficient**	0.42	0.01	0.42	0.01	2.07	-0.26	0.79	0.94
**modular span**	2.24	0.34	2.29	0.34	2.14	-0.12	0.91	0.94

Table S5. Descriptive statistics of BN and BM phases are presented for low-beta band metrics (mean and standard error), along with the statistics of the between-phase comparisons. $|t_{0.05}|$ indicates the two-sided significance threshold (at $p < 0.05$) derived by the permutation test (1000 permutations) on t-statistic, *t*, the t-statistics of the paired t-test, *p* the non-corrected significance of the test (based on the permutations) and p_{FDR} the significance Benjamini-Hochberg correction.

high-beta metrics	BN		BM		BN vs BM			
	mean	stde	mean	stde	$\|t_{0.05}\|$	t	p	p_{FDR}
**clustering coefficient**	0.29	0.01	0.29	0.01	2.13	-0.32	0.73	0.85
**global efficiency**	0.31	0.01	0.36	0.02	2.21	1.38	0.18	0.55
**graph strength**	24.19	0.91	23.91	1.04	2.34	0.26	0.77	0.85
**Modularity**	0.12	0.00	0.14	0.01	2.12	-1.99	0.07	0.40
**participation coefficient**	0.45	0.01	0.45	0.01	2.12	0.30	0.79	0.85
**modular span**	2.06	0.28	2.00	0.19	2.25	0.21	0.85	0.85

Table S6. Descriptive statistics of BN and BM phases are presented for high-beta band metrics (mean and standard error), along with the statistics of the between-phase comparisons. $|t_{0.05}|$ indicates the two-sided significance threshold (at $p < 0.05$) derived by the permutation test (1000 permutations) on the t-statistics, *t*, the t-statistics of the paired t-test, *p* the non-corrected significance of the test (based on the permutations), and p_{FDR} the significance after Benjamini-Hochberg correction.

gamma metrics	BN		BM		BN vs BM			
	mean	stde	mean	stde	$\|t_{0.05}\|$	t	p	p_{FDR}
**clustering coefficient**	0.26	0.01	0.25	0.01	2.25	1.06	0.32	0.36
**global efficiency**	0.27	0.01	0.32	0.01	2.20	2.25	0.04	0.25
**graph strength**	22.08	0.81	20.93	0.79	2.37	1.46	0.19	0.36
**Modularity**	0.14	0.00	0.13	0.01	2.13	1.01	0.36	0.36
**participation coefficient**	0.42	0.01	0.45	0.01	2.30	-1.37	0.20	0.36
**modular span**	2.06	0.23	2.37	0.23	2.14	-1.05	0.34	0.36

Table S7. Descriptive statistics of BN and BM phases are presented for gamma band metrics (mean and standard error), along with the statistics of the between-phase comparisons. $|t_{0.05}|$ indicates the two-sided significance threshold (at $p < 0.05$) derived by the permutation test (1000 permutations) on t-statistic, *t*, the t-statistics of the paired t-test, *p* the non-corrected significance of the test (based on the permutations), and p_{FDR} the significance after Benjamini-Hochberg correction.

Psychometric Tests. Each psychometric parameter (PCI dimensions and sub-dimensions and STAI scores) was submitted to a permutation tests on paired t-statistics (Ludbrook and Dudley, 1998, 1000 permutations), and p-values were adjusted using FDR correction for multiple testing (Benjamini-Hochberg, 1995).

	BN		BM		BN vs BM			
	Mean	stde	mean	stde	$\|t_{0.05}\|$	t	p	p_{FDR}
**PCI**								
**positive affect**	1.63	0.29	1.56	0.32	2.19	0.39	0.72	0.90
**joy**	1.79	0.41	1.38	0.40	2.18	2.42	0.03	0.81
**sex**	0.58	0.31	0.67	0.27	2.14	-0.46	0.66	0.90
**love**	2.50	0.41	2.63	0.46	2.27	-0.28	0.80	0.90
**negative affect**	0.60	0.40	0.47	0.29	1.95	0.93	0.43	0.90
**anger**	0.58	0.38	0.29	0.25	2.00	1.74	0.13	0.81
**sad**	0.75	0.52	0.58	0.31	1.98	0.59	0.63	0.90
**fear**	0.46	0.30	0.50	0.33	1.99	-0.56	0.55	0.90
**altered experience**	3.68	0.42	3.73	0.36	2.14	-0.11	0.92	0.92
**body**	2.14	0.44	2.07	0.35	2.10	0.23	0.83	0.90
**time**	1.39	0.43	1.43	0.37	2.19	-0.16	0.86	0.90
**perception**	1.03	0.31	1.14	0.31	2.18	-0.49	0.62	0.90
**meaning**	1.39	0.38	1.53	0.34	2.26	-0.54	0.61	0.90
**visual imagery**	1.49	0.33	1.54	0.27	2.07	-0.35	0.74	0.90
**amount**	3.75	0.63	3.92	0.58	2.27	-0.25	0.80	0.90
**vividness**	3.58	0.35	3.50	0.34	2.15	0.22	0.83	0.90
**attention**	3.65	0.21	4.15	0.33	2.09	-1.42	0.18	0.81
**inward**	3.69	0.31	4.18	0.38	2.11	-1.02	0.34	0.90
**absorption**	3.58	0.38	4.08	0.36	2.32	-1.48	0.18	0.81
**self awareness**	4.16	0.30	4.43	0.25	2.12	-1.50	0.17	0.81
**altered awareness**	1.97	0.40	1.72	0.43	2.19	0.79	0.42	0.90
**internal dialogue**	2.25	0.41	2.00	0.53	2.27	0.57	0.59	0.90
**rationality**	3.97	0.41	4.56	0.38	2.23	-1.83	0.10	0.81
**volition**	3.27	0.41	3.43	0.39	2.20	-0.40	0.69	0.90
**memory**	4.34	0.40	4.49	0.30	2.11	-0.52	0.62	0.90
**arousal**	1.08	0.44	0.96	0.37	2.05	0.56	0.61	0.90
**STAI**	10.08	0.78	10.67	0.91	2.19	-0.98	0.37	0.90

Table S8. Descriptive statistics of both BN and BM phases are presented (mean and standard error), along with the statistics of the between-phase comparisons. $|t_{0.05}|$ indicates the two-sided significance threshold (at $p < 0.05$) derived by the permutation test (1000 permutations) on t-statistic, *t*, the t-statistics of the paired t-test, *p* the non-corrected significance of the test (based on the permutations), and p_{FDR} the significance after Benjamini-Hochberg correction. No significant difference was found for any psychometric parameter.

References

- Benjamini, Y. & Hochberg, Y. Controlling the False Discovery Rate: a practical and powerful approach to multiple testing. *J. Roy. Stat. Soc. B Met.* ***57***, 289-300 (1995).
- Ludbrook, J. & Dudley, H. Why permutation tests are superior to t and F tests in biomedical research. *Am. Stat.* ***52***, 127-132 (1998).
- Nichols, T. E. & Holmes, A. P. Nonparametric permutation tests for functional neuroimaging: a primer with examples. *Hum. Brain Mapp.* ***15***, 1-25 (2001).
- Onslow, A. C., Bogacz, R. & Jones, M. W. Quantifying phase-amplitude coupling in neuronal network oscillations. *Prog. Biophys. Mol. Biol.* ***105***, 49-57 (2011).
- Vinck, M., Oostenveld, R., van Wingerden, M., Battaglia, F. & Pennartz, C. M. An improved index of phase-synchronization for electrophysiological data in the presence of volume-conduction, noise and sample-size bias. *Neuroimage* **55**, 1548-1565 (2011).
- Chennu, S. et al. Brain networks predict metabolism, diagnosis, and prognosis at the bedside in disorders of consciousness. *Brain* ***140***, 2120-2132 (2017).
Chennu, S. et al. Spectral signatures of reorganised brain networks in disorders of consciousness. *Plos Comput. Biol.* ***10***, e1003887 (2014).

SM3. Cardio-Respiratory Parameters

SNB vs SMB comparisons. Each cardio-respiratory parameter (collected respectively during SNB and SMB phases) was submitted to permutation tests on paired t-statistics (Ludbrook and Dudley, 1998, 1000 permutations), and p-values were adjusted using FDR correction for multiple testing (Benjamini-Hochberg, 1995). As apparent from Table S9, we did not find any significant difference either in breathing rate or ECG parameters (i.e. Heart rate and Heart Rate Variability parameters).

parameters	SNB		SMB		SNB VS SMB			
	mean	stde	mean	stde	$\|t_{0.05}\|$	t-value	p	p_{FDR}
**RESP** (br/min)	2.47	0.18	2.57	0.16	2.15	-0.40	0.79	0.79
**HR** (b/min)	63.24	2.16	62.16	1.56	2.26	0.58	0.58	0.66
**HF** (ms^2/Hz)	490.40	149.40	322.48	85.59	2.54	1.72	0.19	0.66
**LF** (ms^2/Hz)	2667.92	773.60	1989.26	409.63	2.34	0.85	0.45	0.66
**LF/HF** (au)	8.52	1.36	9.65	1.76	2.08	-0.66	0.50	0.66
**VLF** (ms^2/Hz)	380.78	97.52	311.02	73.05	2.25	0.66	0.53	0.66
**RMSSD** (ms)	37.87	6.63	31.88	4.44	2.26	1.30	0.21	0.66
**SDNN** (ms)	56.00	7.78	49.58	4.68	2.13	1.04	0.34	0.66

Table S9. Descriptive statistics of SNB and SMB phases are presented (mean and standard error), along with the between-phase comparisons statistics. $|t_{0.05}|$ indicates the two-sided significance threshold (at p < 0.05) derived by the permutation test (1000 permutations), *t-value*, the t-statistics of the paired t-test, *p-value* the non-corrected significance of the test (based on the permutation), and p_{FDR} the significance after Benjamini-Hochberg correction.

Between-Phase (post-SNB, post-SMB, baseline) comparisons. We verified whether slow breathing (i.e. SNB and/or SMB) induced changes in cardio-respiratory parameters. Each feature was thus submitted to a repeated measures ANOVA with *phase* (post-SNB, post-SMB, baseline), as a three-levels within factor. *Phase* significance was assessed using a permutation test on F-statistics (1000 permutations). The resulting p-values (one for each tested parameter) were adjusted using FDR correction. No parameter showed any significant *phase*-effect (see Table C2 below), hence no post-hoc analysis was conducted.

	post-SNB		post-SMB		baseline		ANOVA RM			
	Mean	stde	mean	stde	mean	stde	$F_{0.05}$	F	p	p_{FDR}
**RESP**	7.82	0.93	8.58	1.22	10.02	0.94	3.31	2.09	0.14	0.36
**HR**	60.67	2.10	58.55	1.54	61.47	1.81	3.30	3.21	0.06	0.36
**HF**	742.13	107.74	480.73	45.12	586.88	156.71	2.24	0.42	0.81	0.90
**LF**	1027.92	89.79	1026.59	100.61	884.43	85.94	2.96	0.13	0.89	0.90
**LF/HF**	2.95	0.65	2.44	0.51	2.11	0.59	3.53	1.18	0.30	0.60
VLF	*80.78*	*22.78*	*79.70*	*13.16*	*40.98*	*6.47*	*3.11*	*4.13*	*0.01*	*0.07*
**RMSSD**	37.27	9.88	34.67	6.14	33.85	5.82	2.60	0.13	0.90	0.90
**SDNN**	40.21	8.44	38.16	5.09	34.62	5.30	2.55	0.43	0.71	0.90

Table S10. Descriptive statistics of post-SNB, post-SMB and baseline phases are presented (mean and standard error), along with the Repeated Measures ANOVA statistics (*phase*: baseline, post-SNB, post-SMB, baseline, as a three-levels within factor. $|F_{0.05}|$ indicates the *phase*-effect threshold for significance (at p < 0.05) derived by the permutation test (1000 permutations), *F-value*, the statistics of the Repeated Measures ANOVA, *p-value* the non-corrected significance of the test (based on the permutation test), and p_{FDR} the significance after Benjamini-Hochberg correction.

	post-SNB vs baseline				post-SMB vs baseline				post-SNB vs post-SMB			
	$t_{\|0.05\|}$	t	p	p_{BH}	$t_{\|0.05\|}$	t	p	p_{BH}	$t_{\|0.05\|}$	t	p	p_{BH}
**VLF**	**1.96**	**2.15**	**0.024**	**0.05**	**1.96**	**3.86**	**0.001**	**0.003**	1.94	0.06	0.95	0.95

Table S11. Post-hoc analyses are presented for HRV VLF. For each comparison, $t_{|0.05|}$ indicates the two-sided significance threshold (at $p < 0.05$) derived by the permutation test (1000 permutations) on t-statistic, *t*, the t-statistics of the paired t-test, *p* the non-corrected significance of the test (based on the permutations), and p_{BH}, significance after Bonferroni-Holm correction.

References

- Ludbrook, J. & Dudley, H. Why permutation tests are superior to t and F tests in biomedical research. *Am. Stat.* ***52***, 127-132 (1998).
- Benjamini, Y. & Hochberg, Y. Controlling the False Discovery Rate: a practical and powerful approach to multiple testing. *J. Roy. Stat. Soc. B Met.* ***57***, 289-300 (1995).
- Holm, S. A simple sequentially rejective multiple test procedure. *Scand. J. Stat.* ***6***, 65-70 (1979).

SM4. Power Spectral Density

Power Spectral Density, PSD.

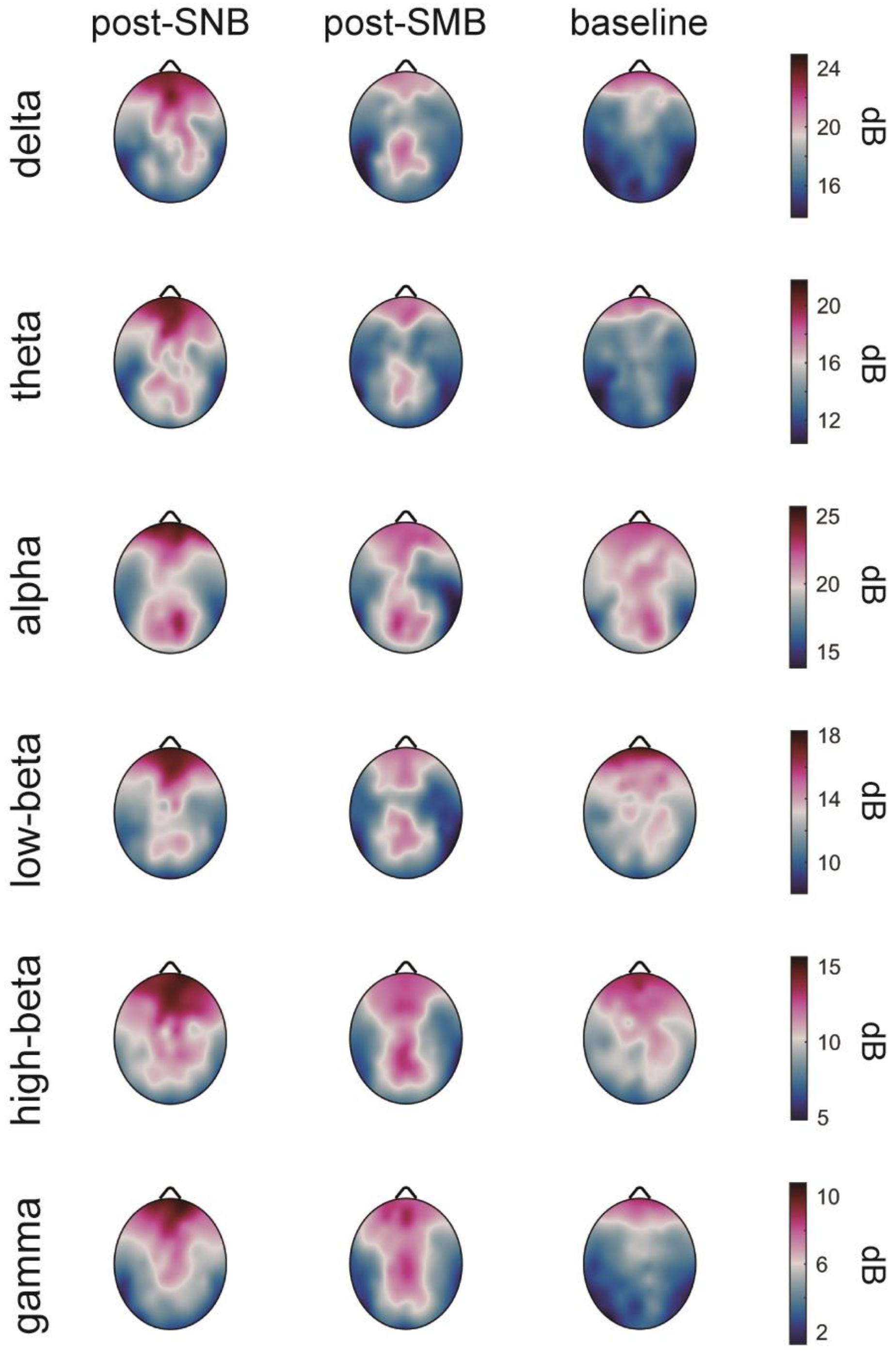

Figure S7. Average PSD scalp maps are presented for each band and phase. For visual clarity, each band has its own colorbar and PSDs are presented in decibels.

For each band and channel, a repeated-measures ANOVA with *phase* as a three-levels within factor (post-SNB, and post-SMB, baseline) was conducted. For each band, *phase* significance at each channel was assessed using a single threshold premutation test for the maximum F-statistic (1000 permutations).

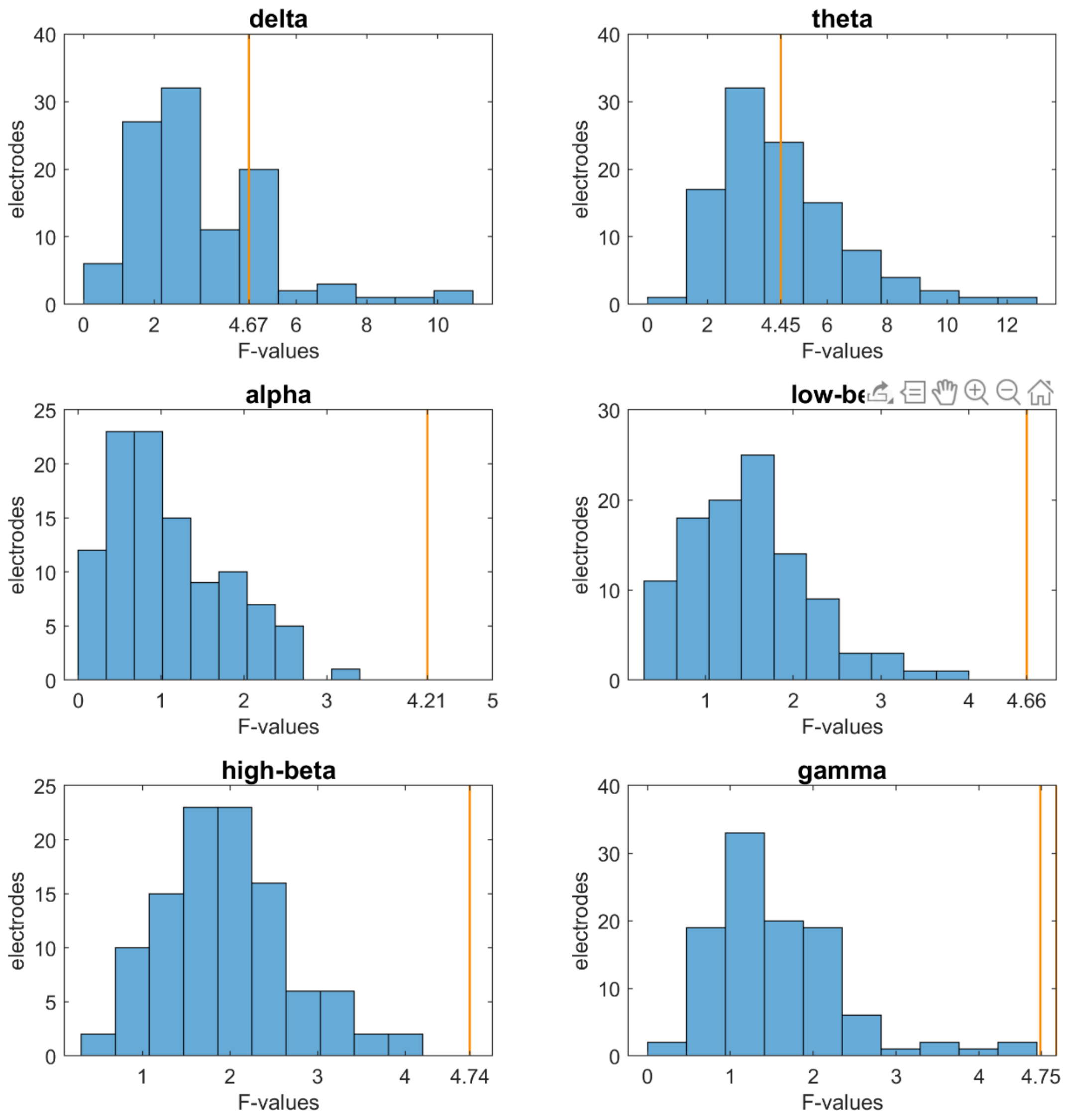

Figure S8. The distributions of F-values related to the electrode-wise Repeated Measures ANOVA are presented for each band (blue bars). In each plot the F-threshold for significance at $p < 0.05$ (estimated using a single threshold permutation test for the maximum F-statistics, 1000 permutations), is denoted by an orange line.

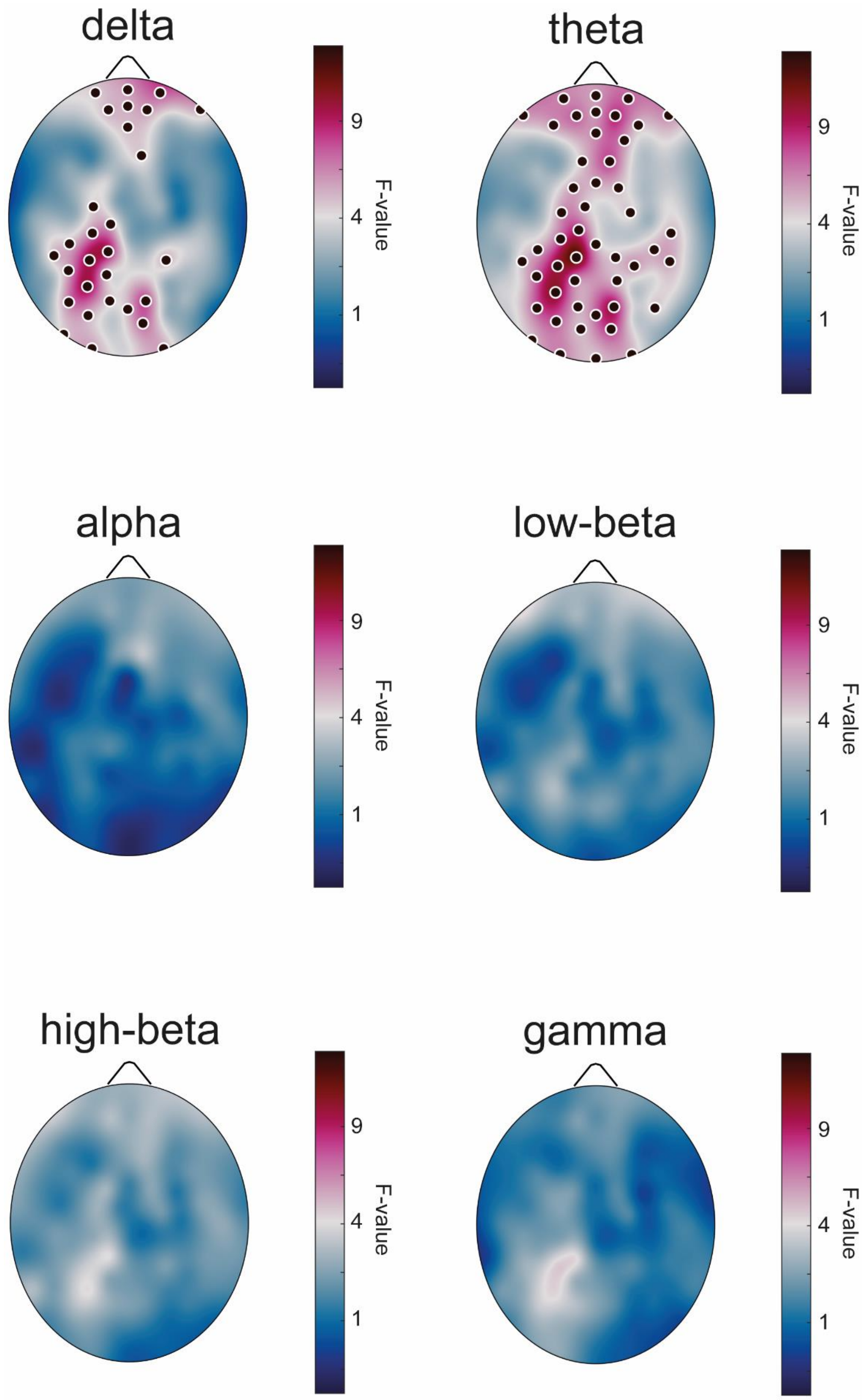

Figure S9. Topographic maps of F-values distributions (within subject *phase*-effect: post-SNB, post-SMB, baseline) are presented for each band. Black dots indicate electrodes showing a significant *phase*-effect ($p < 0.05$, single threshold permutation test for the maximum F-statistics).

Post-hocs tables

Delta

electrode	post-SNB vs post-SMB			post-SNB vs baseline			post-SMB vs baseline		
	$\|t_{0.05}\|$	t-value	p_{BH}	$\|t_{0.05}\|$	t-value	p_{BH}	$\|t_{0.05}\|$	t-value	p_{BH}
**E2**	2.19	3.05	0.006	2.03	1.97	0.116	2.19	-1.11	0.307
**E9**	2.18	3.24	0.012	2.15	2.85	0.022	2.32	-1.18	0.253
**E10**	2.10	2.86	0.036	2.07	2.82	0.012	2.31	0.03	0.979
**E11**	1.92	2.53	0.050	2.18	3.22	0.009	2.27	0.43	0.694
**E15**	2.17	3.16	0.009	2.06	2.58	0.026	2.20	-0.88	0.432
**E16**	2.06	2.58	0.027	2.03	3.07	0.027	2.32	-0.02	0.975
**E18**	2.22	2.69	0.036	2.11	2.83	0.036	2.36	-0.38	0.713
**E22**	2.22	2.81	0.049	2.10	2.47	0.051	2.35	-0.58	0.580
**E37**	2.18	1.20	0.259	2.16	3.17	0.027	2.28	2.62	0.062
**E42**	2.18	1.08	0.310	2.16	3.23	0.003	2.29	1.99	0.164
**E47**	2.27	0.59	0.555	2.23	2.97	0.012	2.36	2.66	0.056
**E52**	2.16	0.83	0.453	2.09	3.02	0.012	2.40	3.34	0.020
**E53**	2.06	0.94	0.392	2.24	4.16	0.003	2.30	3.18	0.028
**E54**	2.11	0.78	0.470	2.24	4.32	0.006	2.28	3.54	0.016
**E59**	2.18	0.66	0.527	2.01	2.80	0.012	2.17	3.53	0.012
**E60**	2.22	1.20	0.254	2.16	4.12	0.003	2.32	3.80	0.012
**E66**	2.04	0.60	0.609	2.08	3.28	0.009	2.24	3.64	0.014
**E72**	1.89	0.92	0.438	2.27	3.16	0.033	2.32	2.64	0.038
**E76**	2.17	1.71	0.128	2.23	3.30	0.024	2.26	2.87	0.026
**E77**	2.16	1.49	0.166	2.14	3.52	0.018	2.36	3.23	0.024
**E83**	2.14	2.26	0.072	2.16	2.76	0.021	2.22	1.34	0.192
**E86**	2.04	1.75	0.254	2.25	3.14	0.021	2.21	1.37	0.254

Table S12. Results of post-hoc analyses are presented for those electrode showing a significant phase-effect in delta band. For each electrode and comparison (i.e. post-SNB vs post-SMB, post-SNB vs baseline and post-SMB vs baseline) three statistics are presented: $|t_{0.05}|$ indicates the two-sided significance threshold (at $p < 0.05$) derived by the permutation test on t-statistic (1000 permutations), *t-value*, the t-statistics of the paired t-test, and p_{BH} the test significance after Bonferroni-Holm correction.

Theta

electrode	post-SNB vs post-SMB			post-SNB vs baseline			post-SMB vs baseline		
	$\lvert t_{0.05} \rvert$	t-value	p_{BH}	$\lvert t_{0.05} \rvert$	t-value	p_{BH}	$\lvert t_{0.05} \rvert$	t-value	p_{BH}
**E2**	2.05	3.03	0.01	2.22	2.67	0.01	2.26	-0.67	0.53
**E3**	2.15	2.18	0.09	2.06	3.42	0.01	2.22	-0.23	0.85
**E4**	2.20	2.56	0.03	2.04	3.29	0.01	2.05	-0.23	0.86
**E5**	2.29	2.89	0.04	2.13	4.07	0.00	2.21	0.31	0.77
**E6**	2.32	1.77	0.20	2.32	3.39	0.03	2.21	1.34	0.21
**E7**	2.01	1.08	0.34	2.11	2.94	0.04	2.29	2.17	0.12
**E9**	2.19	3.16	0.01	2.13	3.65	0.00	2.20	-0.53	0.63
**E10**	2.14	2.75	0.01	2.15	4.03	0.00	2.28	0.38	0.71
**E11**	1.86	2.43	0.01	2.15	4.27	0.00	2.13	0.27	0.82
**E12**	2.12	2.41	0.06	2.10	3.26	0.00	2.18	0.00	1.00
**E13**	2.25	2.32	0.09	1.99	3.15	0.00	2.30	0.85	0.41
**E15**	2.06	2.96	0.01	2.09	3.50	0.00	1.91	-0.44	0.75
**E16**	2.00	2.57	0.02	2.05	3.95	0.00	2.12	-0.06	0.97
**E18**	2.03	2.99	0.01	2.08	4.23	0.00	2.16	-0.36	0.74
**E22**	2.19	3.11	0.02	2.11	3.35	0.00	2.15	-0.38	0.73
**E23**	2.17	3.11	0.02	2.19	3.45	0.00	2.16	-0.24	0.83
**E26**	2.14	3.08	0.04	2.25	2.35	0.06	2.36	-0.86	0.44
**E30**	2.06	1.88	0.18	2.20	3.21	0.01	2.25	1.79	0.18
**E31**	2.14	1.21	0.26	2.22	3.26	0.00	2.36	3.56	0.02
**E37**	2.22	1.04	0.31	2.12	3.26	0.01	2.32	3.40	0.02
**E42**	2.22	1.18	0.27	2.21	3.21	0.02	2.39	2.06	0.15
**E47**	2.26	0.87	0.41	2.19	3.21	0.01	2.35	2.28	0.11
**E52**	2.27	1.08	0.30	2.10	3.39	0.00	2.36	3.07	0.03
**E53**	2.16	0.98	0.36	2.09	4.29	0.00	2.34	3.10	0.02
**E54**	2.20	0.91	0.39	2.12	4.65	0.00	2.32	4.17	0.01
**E55**	2.12	0.92	0.39	2.14	3.18	0.01	2.14	2.15	0.10
**E59**	2.24	0.91	0.36	2.12	3.11	0.00	2.13	3.19	0.01
**E60**	2.15	1.43	0.19	2.12	4.13	0.00	2.34	3.62	0.01
**E61**	2.11	-0.04	0.97	2.08	4.41	0.00	2.16	3.01	0.01
**E66**	1.97	0.91	0.43	2.16	3.34	0.00	2.23	4.65	0.00
**E67**	2.08	0.20	0.89	2.26	4.64	0.01	1.97	2.37	0.02
**E71**	1.90	0.42	0.67	2.07	3.91	0.01	1.92	2.26	0.04
**E72**	1.72	1.14	0.33	2.13	3.67	0.01	2.33	3.21	0.02
**E75**	2.16	1.15	0.28	2.11	2.52	0.05	2.24	2.16	0.12
**E76**	2.15	2.12	0.07	2.03	3.54	0.01	2.27	2.51	0.07
**E77**	2.18	1.82	0.10	2.02	3.80	0.01	2.30	3.13	0.02
**E78**	2.00	1.39	0.22	1.92	3.16	0.00	2.25	1.83	0.21
**E79**	1.85	1.50	0.27	2.14	3.24	0.00	2.22	1.49	0.27
**E83**	2.17	2.08	0.12	2.02	2.64	0.02	2.16	0.68	0.49
**E86**	2.06	2.04	0.11	2.13	3.60	0.00	2.31	0.99	0.36
**E91**	2.05	2.03	0.11	2.04	3.04	0.01	2.13	0.68	0.52
**E93**	2.09	1.58	0.27	2.11	3.38	0.00	2.25	1.56	0.27
**E98**	2.09	1.79	0.19	2.14	3.01	0.01	2.22	1.72	0.19
**E103**	2.17	1.67	0.17	2.12	2.66	0.04	2.12	1.89	0.17
**E105**	2.18	1.92	0.17	2.19	3.07	0.03	2.31	0.80	0.43

**E112**	2.03	2.70	0.03	2.20	2.74	0.04	2.16	-0.32	0.75

Table S13. Results of post-hoc analyses are presented for those electrode showing a significant phase-effect in theta band. For each electrode and comparison (i.e. post-SNB vs post-SMB, post-SNB vs baseline and post-SMB vs baseline) three statistics are presented: $|t_{0.05}|$ indicates the two-sided significance threshold (at $p < 0.05$) derived by the permutation test on t-statistic (1000 permutations), *t-value*, the t-statistics of the paired t-test, and p_{BH} the test significance after Bonferroni-Holm correction.

Please note that in the post-hoc tables, the statistics of those electrodes showing both a significant phase-effect and at least one significant post-hoc comparison are reported.

SM5. Connectivity

For each band and couple of channels, a repeated-measures ANOVA with *phase* as a three-levels within factor (post-SNB, and post-SMB, baseline) was conducted. For each band, *phase* significance at each couple was assessed using a single threshold premutation test for the maximum F-statistic (1000 permutations).

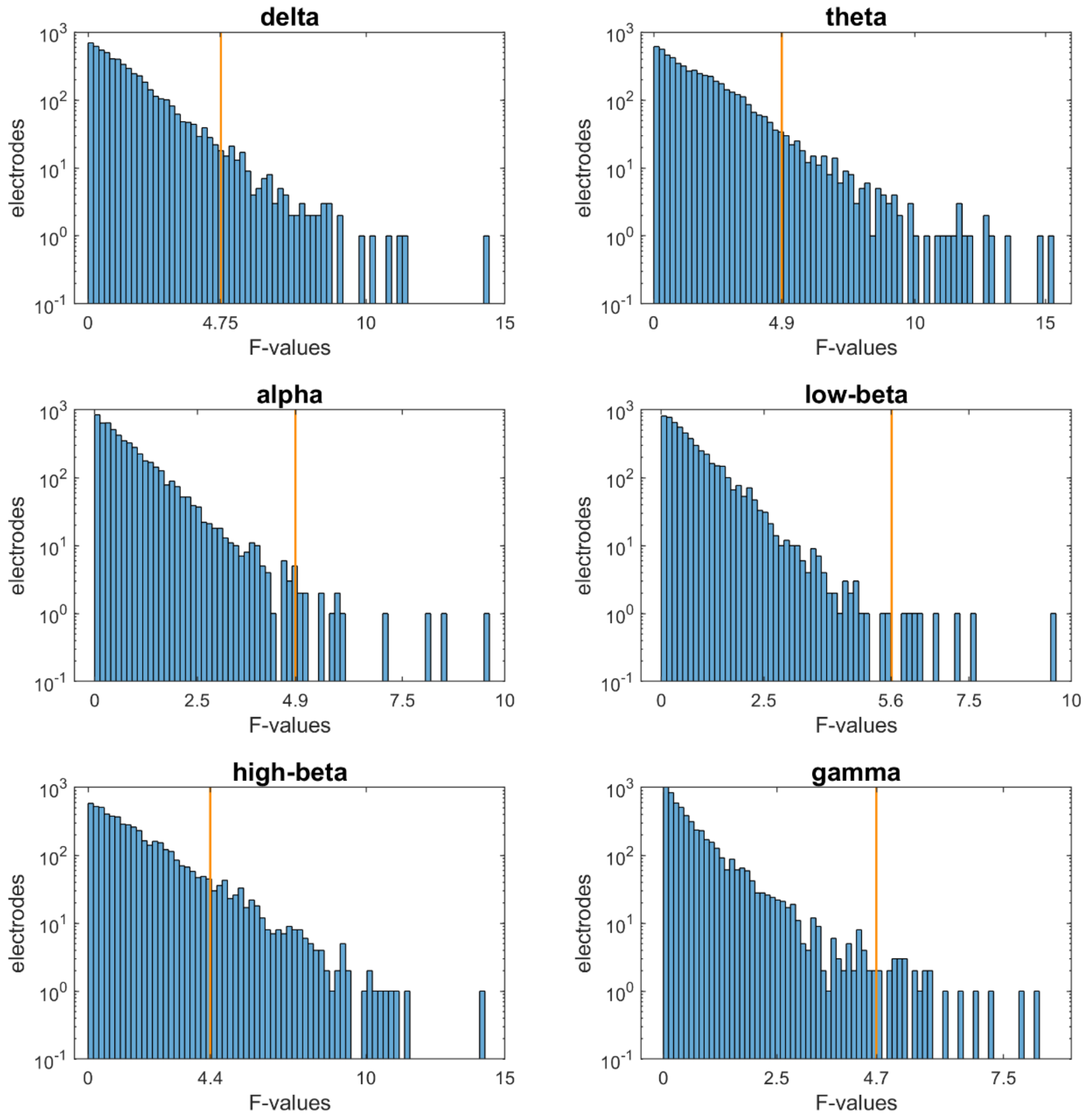

Figure S10. The distributions of F-values related to the electrode-wise Repeated Measures ANOVA are presented for each band (blue bars). In each plot the F-threshold for significance at $p < 0.05$ (estimated using a single threshold permutation test for the maximum F-statistics, 1000 permutations), is denoted by an orange line.

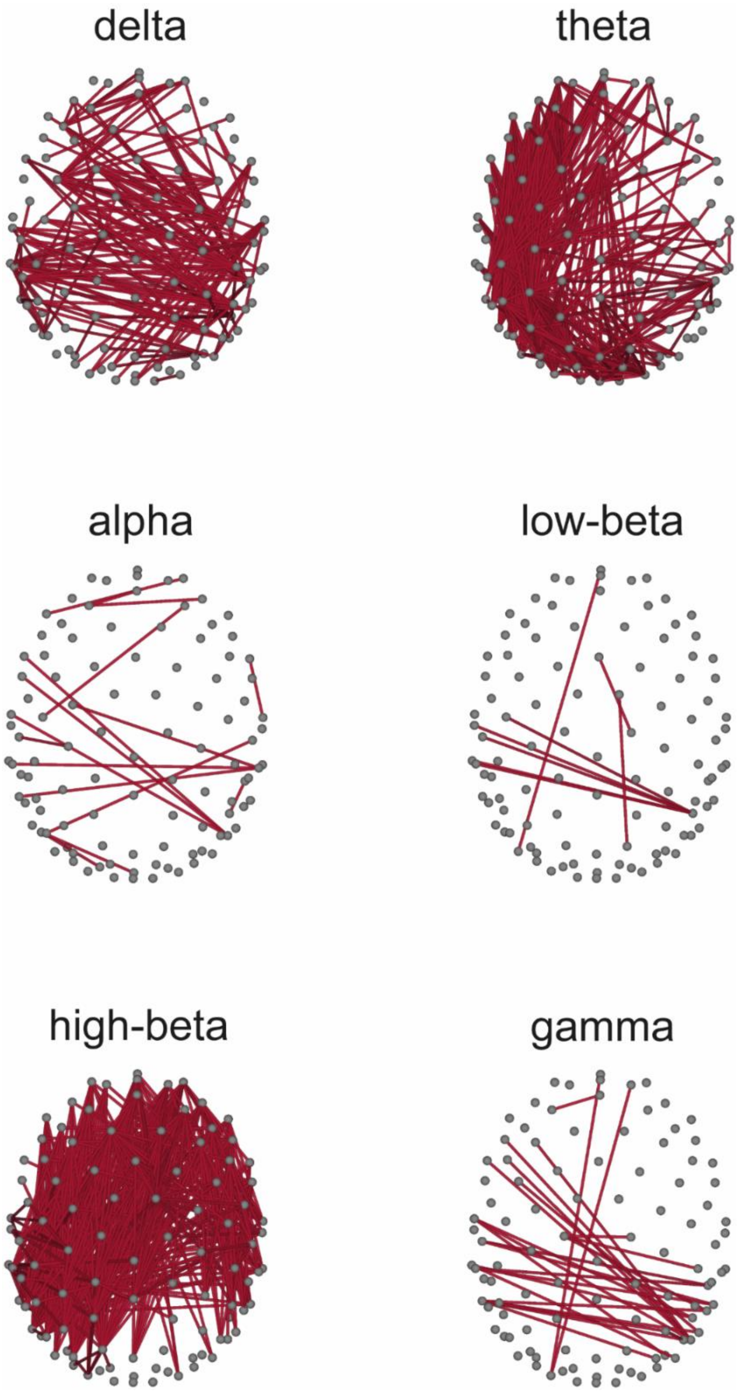

Figure S11. Topographic maps of F-values distributions (within subject *phase*-effect: post-SNB, post-SMB, baseline) are presented for each band and couples of channels (i.e. dWPLI-connectivity). Red lines denote those connectivities showing a significant *phase*-effect ($p < 0.05$, single threshold permutation test for the maximum F-statistics).

A massive number of significant connectivities (i.e. *phase*-effect) was found in delta, theta and high-beta bands, whereas an extremely lower number was found when considering alpha, low-beta and gamma bands:

- **delta 167 out of 5460 couples.**
- **theta 312 out of 5460 couples.**
- alpha 16 out of 5460 couples.
- low-beta 8 out of 5460 couples.

- **high-beta 390 out of 5460 couples.**
- gamma 26 out of 5460 couples.

Alpha, low-beta and gamma post-hocs

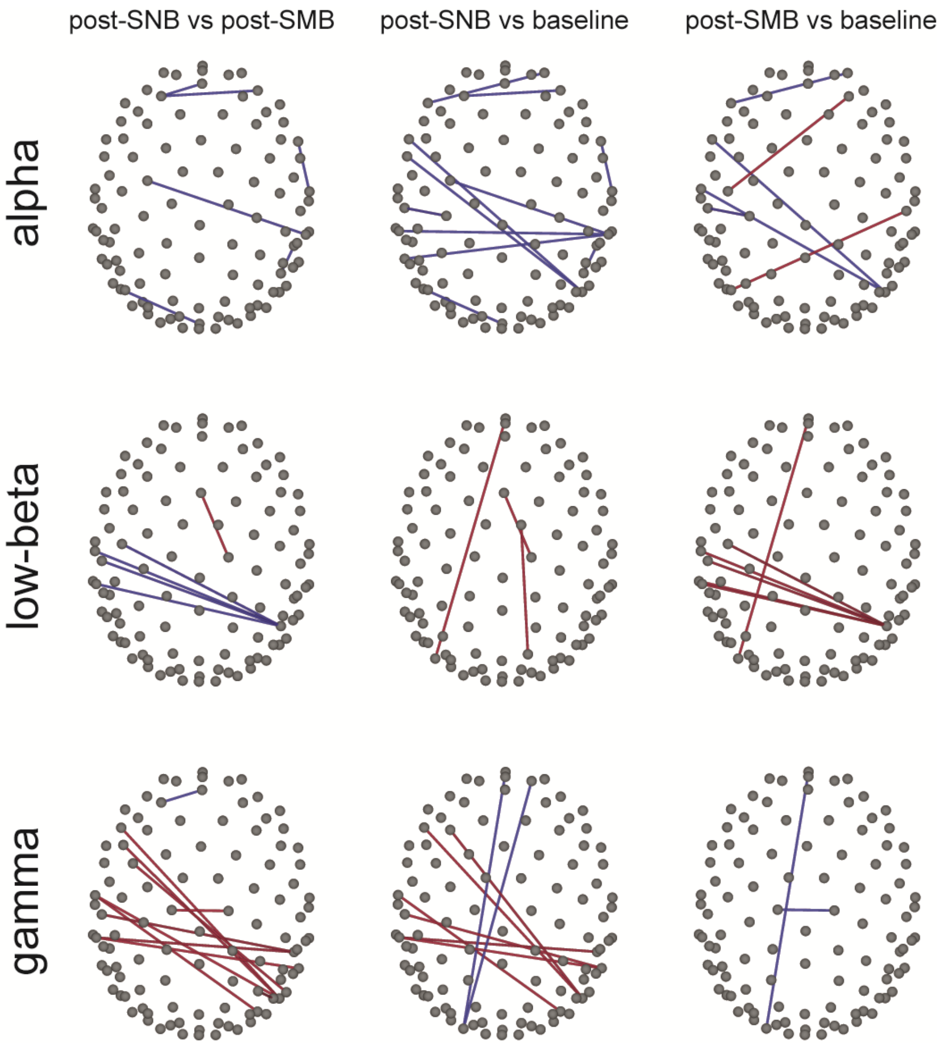

Figure S12. Significant connectivity differences are presented for each band and post-hoc. Red lines indicate higher connectivity in the former phase as compared to the latter, blue lines the opposite relationship.

Post-hocs tables

Delta

electrodes couples	post-SNB vs post-SMB			post-SNB vs baseline			post-SMB vs baseline		
	$\lvert t_{0.05}\rvert$	t-value	p_{BH}	$\lvert t_{0.05}\rvert$	t-value	p_{BH}	$\lvert t_{0.05}\rvert$	t-value	p_{BH}
**E2-E29**	2.20	2.03	0.128	2.19	4.74	0.003	2.23	0.29	0.759
**E4-E29**	2.07	1.76	0.214	2.44	7.70	0.003	2.24	0.71	0.476
**E5-E107**	2.23	3.55	0.004	2.37	4.16	0.003	2.25	0.52	0.589
**E6-E104**	2.12	2.89	0.048	2.31	2.12	0.140	2.22	-1.22	0.238
**E6-E105**	2.19	3.14	0.003	2.38	2.16	0.148	2.13	-1.39	0.196
**E6-E107**	2.18	3.05	0.028	2.34	5.04	0.003	2.26	1.26	0.234
**E6-E111**	2.19	2.68	0.027	2.23	2.28	0.088	2.22	0.26	0.810
**E7-E31**	2.30	3.97	0.009	2.22	1.86	0.184	2.19	-1.67	0.184
**E7-E99**	2.21	2.34	0.074	2.35	3.11	0.039	2.12	0.62	0.561
**E7-E104**	2.02	2.77	0.009	1.94	2.02	0.086	2.22	-0.20	0.830
**E7-E107**	2.27	2.32	0.094	2.21	3.95	0.003	2.17	0.88	0.393
**E9-E19**	2.20	1.43	0.172	2.24	5.28	0.003	2.17	1.93	0.172
**E9-E24**	2.17	0.99	0.346	2.22	3.59	0.006	2.36	2.13	0.136
**E9-E29**	2.28	1.37	0.318	2.19	4.60	0.003	2.16	1.60	0.318
**E9-E107**	2.16	1.58	0.162	2.32	3.17	0.036	2.34	1.91	0.162
**E10-E19**	2.12	1.12	0.320	2.17	4.13	0.003	2.23	2.10	0.140
**E10-E20**	2.03	1.58	0.262	2.18	3.54	0.009	2.23	1.45	0.262
**E10-E24**	2.18	1.74	0.107	2.09	3.41	0.003	2.30	2.35	0.086
**E10-E29**	2.24	1.77	0.208	2.31	5.64	0.003	2.26	1.25	0.253
**E11-E29**	2.12	2.60	0.050	2.25	3.97	0.009	2.10	-0.08	0.938
**E11-E107**	2.37	3.58	0.018	2.40	3.21	0.026	2.20	0.73	0.492
**E11-E111**	2.25	3.14	0.048	2.29	2.41	0.090	2.22	-0.14	0.872
**E12-E28**	2.15	1.79	0.214	2.26	3.12	0.021	2.17	1.10	0.286
**E12-E29**	2.13	2.25	0.086	2.31	3.37	0.033	2.14	0.07	0.945
**E12-E107**	2.29	3.05	0.028	2.32	3.37	0.021	2.23	1.03	0.335
**E12-E118**	2.08	0.67	0.508	2.24	3.19	0.036	2.20	2.45	0.064
**E13-E41**	2.24	2.41	0.080	2.07	3.44	0.036	2.27	0.10	0.931
**E13-E50**	2.16	2.77	0.048	2.22	2.98	0.027	2.24	-0.55	0.606
**E13-E93**	2.00	3.14	0.012	2.26	2.03	0.154	2.18	-1.56	0.159
**E13-E98**	1.97	2.80	0.030	2.22	2.23	0.098	2.12	-0.61	0.562
**E13-E102**	2.01	2.62	0.026	2.03	3.11	0.021	2.19	0.97	0.355
**E13-E103**	2.03	3.18	0.003	2.12	3.40	0.010	2.28	0.16	0.883
**E13-E104**	2.15	4.13	0.003	2.11	3.06	0.014	2.12	-1.49	0.171
**E13-E105**	2.17	2.83	0.046	2.12	2.04	0.114	2.22	-0.48	0.654
**E13-E107**	2.36	4.90	0.006	2.35	5.29	0.003	2.17	0.85	0.419
**E13-E109**	2.23	3.41	0.009	2.25	2.93	0.010	2.30	-0.71	0.493
**E13-E110**	2.19	3.16	0.015	2.27	2.83	0.015	2.23	0.27	0.790
**E13-E111**	2.16	3.24	0.018	2.18	2.69	0.018	2.28	0.05	0.954
**E15-E19**	2.22	2.74	0.044	2.30	5.68	0.003	2.39	1.10	0.295
**E15-E24**	2.22	1.62	0.238	2.13	3.60	0.003	2.37	1.63	0.238
**E16-E19**	2.22	1.42	0.232	2.15	3.40	0.015	2.35	1.72	0.232
**E16-E107**	2.18	2.46	0.044	2.38	2.92	0.051	2.25	0.89	0.376
**E19-E107**	2.19	1.73	0.232	2.31	3.51	0.012	2.19	1.53	0.232
**E23-E24**	2.26	2.45	0.070	2.25	3.06	0.027	2.13	0.33	0.769

**E23-E31**	2.25	2.70	0.042	2.27	2.21	0.106	2.21	-1.19	0.256
**E23-E105**	2.34	3.26	0.021	2.33	3.17	0.032	2.30	-0.26	0.776
**E24-E86**	2.21	2.85	0.049	2.22	1.92	0.164	2.30	-1.71	0.164
**E26-E105**	2.16	3.07	0.047	2.25	2.66	0.057	2.03	-0.53	0.665
**E27-E105**	2.35	2.87	0.046	2.32	2.96	0.066	2.30	-0.69	0.476
**E29-E86**	2.16	2.73	0.034	2.31	3.34	0.033	2.17	-0.51	0.623
**E29-E87**	2.30	4.00	0.003	2.23	2.41	0.074	2.17	-0.78	0.467
**E29-E92**	2.19	2.23	0.086	2.30	4.01	0.006	2.07	0.33	0.770
**E29-E93**	2.23	3.03	0.021	2.31	3.33	0.021	2.19	-0.18	0.866
**E29-E98**	2.22	2.51	0.050	2.24	3.64	0.012	2.17	0.41	0.686
**E29-E103**	2.23	1.84	0.184	2.24	3.32	0.012	2.07	1.17	0.289
**E29-E104**	2.28	3.12	0.012	2.24	4.16	0.012	2.21	0.05	0.958
**E29-E105**	2.27	2.16	0.130	2.28	3.32	0.039	2.14	0.80	0.459
**E29-E111**	2.35	2.26	0.114	2.29	3.46	0.045	2.31	0.51	0.629
**E29-E118**	2.12	1.84	0.182	2.16	3.67	0.003	2.16	0.77	0.448
**E30-E64**	2.36	-3.62	0.012	2.20	-2.48	0.058	2.21	1.66	0.126
**E30-E86**	2.24	2.34	0.086	2.32	4.55	0.003	2.16	0.07	0.934
**E30-E87**	2.30	2.68	0.044	2.07	2.65	0.021	2.14	-0.54	0.579
**E30-E92**	2.21	1.68	0.258	2.26	4.78	0.003	2.22	0.83	0.393
**E30-E93**	2.26	2.37	0.070	2.19	3.90	0.003	2.18	0.86	0.387
**E30-E99**	2.15	1.34	0.222	2.31	4.40	0.003	2.14	1.89	0.164
**E30-E104**	2.25	2.15	0.132	2.23	3.23	0.003	2.13	0.83	0.396
**E30-E111**	2.21	2.78	0.044	2.32	3.70	0.009	2.29	0.07	0.949
**E31-E64**	2.43	-3.44	0.045	2.30	-2.46	0.062	2.29	0.50	0.612
**E35-E46**	2.13	-1.86	0.120	2.32	2.17	0.120	2.18	3.11	0.045
**E36-E93**	2.27	1.84	0.206	2.26	3.55	0.012	2.24	1.14	0.268
**E36-E103**	2.21	1.98	0.158	2.27	3.26	0.006	2.16	1.06	0.303
**E37-E99**	2.21	1.34	0.217	2.15	3.06	0.018	2.03	1.86	0.174
**E40-E93**	2.23	2.77	0.048	2.12	3.53	0.030	2.17	0.36	0.704
**E40-E98**	2.32	2.32	0.098	2.22	4.22	0.009	2.19	1.05	0.312
**E40-E103**	2.21	1.79	0.196	2.28	4.35	0.006	2.22	1.09	0.284
**E41-E46**	2.14	-3.83	0.009	2.22	1.19	0.242	2.33	3.58	0.010
**E41-E50**	2.18	1.23	0.251	2.46	3.78	0.006	2.21	1.81	0.196
**E41-E85**	2.26	1.61	0.294	2.24	4.94	0.006	2.18	1.05	0.308
**E41-E92**	2.16	1.32	0.219	2.33	5.40	0.003	2.18	2.09	0.118
**E41-E93**	2.26	1.43	0.214	2.25	4.08	0.033	2.21	1.74	0.214
**E41-E97**	2.33	1.09	0.301	2.34	4.42	0.009	2.09	2.32	0.064
**E45-E59**	2.21	2.86	0.044	2.12	2.51	0.054	2.18	-0.22	0.832
**E45-E60**	2.26	2.97	0.041	2.19	2.36	0.070	2.15	-0.71	0.488
**E45-E85**	2.11	2.10	0.100	2.25	3.19	0.024	2.18	0.88	0.390
**E45-E86**	2.27	2.52	0.078	2.26	4.07	0.012	2.11	1.09	0.315
**E45-E91**	2.28	1.98	0.170	2.27	4.08	0.012	2.26	1.85	0.170
**E45-E92**	2.32	1.98	0.142	2.26	5.55	0.003	2.29	1.77	0.142
**E45-E93**	2.25	1.97	0.174	2.32	3.61	0.012	2.19	0.93	0.357
**E45-E98**	2.31	1.97	0.154	2.31	3.56	0.024	2.24	1.51	0.169
**E45-E102**	2.32	2.12	0.136	2.23	5.37	0.003	2.19	0.80	0.437
**E45-E103**	2.32	2.94	0.032	2.27	6.86	0.003	2.16	0.74	0.476
**E45-E104**	2.27	3.03	0.020	2.30	5.09	0.006	2.08	-0.28	0.773
**E45-E105**	2.45	2.65	0.060	2.22	4.40	0.003	2.30	0.89	0.373

**E45-E110**	2.36	2.39	0.096	2.23	3.77	0.009	2.23	-0.07	0.948
**E46-E85**	2.19	1.76	0.214	2.38	5.00	0.009	2.22	1.10	0.276
**E46-E92**	2.17	1.48	0.298	2.34	4.46	0.003	2.20	1.60	0.298
**E46-E97**	2.29	1.24	0.244	2.29	4.06	0.012	2.25	2.11	0.116
**E47-E91**	2.22	1.82	0.188	2.44	3.83	0.012	2.19	1.18	0.249
**E47-E93**	2.24	2.56	0.074	2.48	3.43	0.045	2.38	0.78	0.439
**E47-E97**	2.22	2.15	0.112	2.48	4.55	0.003	2.25	1.79	0.112
**E47-E98**	2.20	1.82	0.170	2.49	3.69	0.045	2.37	1.90	0.170
**E47-E103**	2.17	1.20	0.242	2.28	4.01	0.012	2.21	1.73	0.242
**E50-E51**	2.13	2.09	0.122	2.34	4.01	0.006	2.26	0.29	0.780
**E50-E52**	2.23	2.07	0.140	2.32	4.17	0.003	2.25	0.72	0.451
**E51-E90**	2.29	2.22	0.112	2.22	4.26	0.003	2.24	0.30	0.749
**E52-E94**	2.26	3.21	0.018	2.20	1.91	0.162	2.24	-1.26	0.240
**E53-E99**	2.27	1.60	0.150	2.24	2.93	0.045	2.21	2.44	0.070
**E60-E94**	2.21	2.81	0.049	2.29	2.29	0.098	2.13	-0.50	0.645
**E61-E66**	2.30	1.06	0.309	2.08	-2.22	0.080	2.40	-3.17	0.030
**E61-E99**	2.35	2.39	0.086	2.25	3.04	0.030	2.28	-0.12	0.918
**E67-E94**	2.21	2.83	0.043	2.19	2.54	0.063	2.17	-0.20	0.837
**E69-E115**	2.31	-2.83	0.038	2.21	-3.77	0.015	2.25	0.69	0.511
**E70-E115**	2.21	-3.62	0.018	2.30	-1.82	0.104	2.36	2.66	0.048
**E71-E94**	2.42	2.20	0.114	2.27	3.94	0.015	2.17	1.14	0.308
**E71-E115**	2.27	-3.26	0.021	2.00	-2.51	0.054	2.33	2.21	0.057
**E71-E122**	2.21	-3.42	0.024	2.15	-0.96	0.327	2.37	2.49	0.082
**E72-E94**	2.31	2.26	0.108	2.28	3.32	0.036	2.18	0.65	0.556
**E72-E115**	2.20	-3.29	0.021	2.22	-2.78	0.050	2.32	1.85	0.103
**E75-E107**	2.43	-3.26	0.039	2.16	-1.34	0.246	2.36	1.76	0.246
**E75-E115**	2.26	-2.67	0.044	2.30	-1.33	0.219	2.13	2.75	0.024
**E76-E115**	2.26	-3.35	0.024	2.40	-2.51	0.088	2.22	1.81	0.109
**E77-E115**	2.28	-3.36	0.021	2.28	-3.27	0.021	2.34	1.21	0.262
**E78-E99**	2.35	2.28	0.108	2.26	3.23	0.039	2.33	-0.19	0.857
**E82-E83**	2.37	-2.91	0.036	2.26	-0.89	0.372	2.10	2.00	0.120
**E84-E85**	2.46	4.15	0.009	2.41	2.35	0.100	2.21	-0.49	0.611
**E85-E92**	2.27	2.86	0.044	2.35	3.11	0.048	2.35	1.38	0.202
**E86-E99**	2.42	4.17	0.003	2.45	3.74	0.004	2.14	-1.12	0.268
**E92-E99**	2.27	3.27	0.014	2.43	3.64	0.012	2.27	-0.24	0.799
**E93-E99**	2.17	3.21	0.024	2.20	2.34	0.068	2.23	-1.30	0.201
**E96-E99**	2.32	3.04	0.044	2.31	2.85	0.054	2.29	1.13	0.273
**E107-E112**	2.24	1.69	0.238	2.36	3.92	0.003	2.30	1.49	0.238
**E110-E124**	2.30	2.49	0.076	2.27	3.11	0.021	2.34	0.33	0.743
**E116-E118**	2.42	2.46	0.090	2.11	3.03	0.024	2.27	0.71	0.468
**E117-E124**	2.36	3.53	0.021	2.38	3.12	0.024	2.19	0.15	0.880

Table S14. Results of post-hoc analyses are presented for those electrode couples (i.e. connectivities), showing a significant phase-effect in delta band. For each couple and comparison (i.e. post-SNB vs post-SMB, post-SNB vs baseline and post-SMB vs baseline) three statistics are presented: $|t_{0.05}|$ indicates the two-sided significance threshold (at $p < 0.05$) derived by the permutation test on t-statistic (1000 permutations), *t-value*, the t-statistics of the paired t-test, and p_{BH} the test significance after Bonferroni-Holm correction.

electrodes couples	Theta								
	post-SNB vs post-SMB			post-SNB vs baseline			post-SMB vs baseline		
	$\lvert t_{0.05} \rvert$	t-value	p_{BH}	$\lvert t_{0.05} \rvert$	t-value	p_{BH}	$\lvert t_{0.05} \rvert$	t-value	p_{BH}
**E2-E47**	2.22	1.51	0.274	2.30	4.91	0.003	2.20	1.62	0.274
**E2-E82**	2.13	2.98	0.033	2.16	1.70	0.244	2.14	-1.25	0.247
**E3-E118**	2.12	0.79	0.453	2.34	3.79	0.003	2.24	2.60	0.062
**E4-E6**	2.18	1.87	0.188	2.16	3.83	0.006	2.33	1.24	0.245
**E4-E42**	2.31	2.07	0.140	2.20	3.48	0.015	2.15	0.74	0.487
**E5-E42**	2.33	2.06	0.158	2.27	3.48	0.003	2.13	0.85	0.414
**E5-E70**	2.18	2.75	0.049	2.26	2.29	0.094	2.30	0.27	0.778
**E6-E58**	2.24	2.19	0.104	2.29	3.29	0.042	2.20	1.00	0.321
**E6-E60**	2.27	2.31	0.080	2.31	3.46	0.036	2.20	0.60	0.567
**E6-E64**	2.23	2.88	0.048	2.29	3.42	0.015	2.33	0.59	0.545
**E6-E65**	2.22	3.17	0.027	2.22	3.16	0.028	2.16	0.49	0.613
**E6-E66**	2.19	2.76	0.048	2.32	3.36	0.027	2.18	0.72	0.466
**E6-E67**	2.26	2.13	0.130	2.24	3.42	0.030	2.30	0.62	0.537
**E6-E69**	2.37	3.32	0.030	2.20	2.79	0.042	2.16	-0.46	0.666
**E6-E70**	2.24	2.67	0.050	2.36	3.24	0.033	2.30	0.79	0.438
**E6-E71**	2.21	2.13	0.112	2.25	3.16	0.033	2.25	0.75	0.449
**E6-E75**	2.15	1.65	0.254	2.23	3.52	0.015	2.29	1.49	0.254
**E6-E82**	2.15	2.78	0.024	2.17	3.04	0.018	2.16	-0.34	0.729
**E6-E83**	2.18	2.47	0.050	1.98	2.42	0.024	2.19	-0.48	0.629
**E7-E65**	2.20	1.32	0.214	2.32	3.59	0.027	2.31	1.96	0.170
**E9-E47**	2.22	1.62	0.212	2.27	4.56	0.003	2.28	1.71	0.212
**E9-E51**	2.17	2.28	0.084	2.15	4.26	0.003	2.16	1.22	0.233
**E9-E58**	2.31	3.27	0.018	2.25	3.87	0.003	2.17	0.97	0.335
**E9-E82**	2.12	2.82	0.049	2.16	1.82	0.188	2.02	-1.16	0.286
**E9-E118**	2.13	1.46	0.258	2.28	4.44	0.009	2.30	1.63	0.258
**E10-E16**	2.07	2.88	0.022	2.26	0.20	0.829	2.19	-3.50	0.015
**E10-E42**	2.23	1.93	0.164	2.25	3.50	0.018	2.18	0.90	0.363
**E10-E47**	2.27	1.79	0.194	2.19	4.28	0.003	2.29	1.48	0.194
**E10-E51**	2.20	2.83	0.028	2.20	3.94	0.006	2.13	0.83	0.419
**E10-E58**	2.30	3.78	0.003	2.23	3.45	0.004	2.01	0.15	0.890
**E11-E51**	2.20	2.62	0.042	2.17	3.81	0.012	2.10	1.25	0.234
**E11-E56**	2.24	1.32	0.218	2.26	3.84	0.006	2.26	2.31	0.088
**E11-E58**	2.26	4.06	0.003	2.22	3.62	0.006	2.09	1.35	0.187
**E12-E23**	2.17	1.70	0.226	2.21	3.80	0.003	2.31	1.51	0.226
**E12-E42**	2.22	2.03	0.132	2.15	3.01	0.033	2.21	1.11	0.284
**E12-E58**	2.28	2.22	0.110	2.22	2.98	0.039	2.25	1.92	0.110
**E13-E19**	2.09	1.30	0.220	2.23	4.06	0.003	2.12	2.32	0.066
**E13-E23**	2.11	1.16	0.249	2.30	3.79	0.003	2.19	2.25	0.090
**E15-E42**	2.16	1.61	0.260	2.14	3.75	0.003	2.03	1.58	0.260
**E15-E47**	2.21	1.58	0.226	2.21	4.35	0.003	2.08	1.72	0.226
**E15-E51**	2.25	2.72	0.046	2.24	4.29	0.003	2.06	1.61	0.137
**E15-E52**	2.17	2.76	0.046	2.26	2.64	0.060	2.04	0.53	0.594
**E15-E58**	2.32	3.30	0.006	2.24	3.78	0.003	1.95	1.58	0.126
**E16-E51**	2.26	3.08	0.018	2.26	4.33	0.003	2.04	0.81	0.446

**E16-E58**	2.22	4.44	0.004	2.26	3.76	0.003	1.96	0.63	0.572
**E16-E82**	2.11	2.87	0.049	2.15	1.71	0.138	2.16	-2.02	0.138
**E16-E83**	2.18	3.19	0.012	1.78	1.47	0.290	2.11	-1.52	0.290
**E18-E31**	2.19	1.22	0.244	2.27	4.09	0.006	2.20	1.75	0.202
**E18-E47**	2.22	1.56	0.140	2.21	4.81	0.003	2.15	1.97	0.136
**E18-E51**	2.21	2.80	0.036	2.31	6.18	0.003	2.17	2.13	0.055
**E18-E52**	2.20	2.19	0.100	2.29	4.21	0.003	2.07	1.74	0.104
**E18-E56**	2.20	1.17	0.252	2.22	4.57	0.003	2.13	1.98	0.148
**E18-E58**	2.23	3.34	0.010	2.29	4.87	0.003	2.10	1.89	0.068
**E18-E59**	2.01	1.91	0.076	2.31	3.94	0.015	2.15	2.34	0.076
**E18-E60**	2.14	1.47	0.209	2.24	3.67	0.021	2.07	1.98	0.142
**E18-E65**	2.28	3.10	0.042	2.30	2.94	0.042	2.15	1.86	0.080
**E18-E66**	2.20	1.94	0.162	2.30	2.97	0.048	2.20	1.52	0.162
**E18-E122**	2.08	2.76	0.042	2.35	2.73	0.046	2.30	0.22	0.832
**E19-E42**	2.18	1.76	0.138	2.25	4.66	0.003	2.21	2.05	0.138
**E19-E47**	2.16	1.46	0.157	2.30	5.04	0.003	2.26	2.54	0.050
**E19-E51**	2.28	2.36	0.078	2.29	5.48	0.003	2.16	2.31	0.078
**E19-E52**	2.17	2.28	0.037	2.28	4.72	0.003	2.11	2.68	0.034
**E19-E53**	2.27	0.93	0.383	2.28	3.23	0.024	2.17	2.49	0.038
**E19-E56**	2.19	0.44	0.655	2.32	5.86	0.003	2.41	2.67	0.060
**E19-E58**	2.32	2.40	0.046	2.27	4.50	0.003	2.11	2.41	0.046
**E19-E59**	2.07	1.76	0.108	2.31	4.01	0.006	2.15	2.57	0.064
**E19-E60**	2.06	1.57	0.171	2.32	4.05	0.012	2.12	2.29	0.066
**E19-E65**	2.17	2.91	0.039	2.33	3.06	0.039	2.23	1.94	0.079
**E19-E66**	2.06	2.09	0.088	2.27	3.54	0.027	2.19	1.63	0.124
**E20-E24**	2.27	2.82	0.050	2.42	3.31	0.033	2.23	-0.03	0.963
**E20-E51**	2.24	1.31	0.203	2.32	3.87	0.006	2.20	2.42	0.058
**E20-E52**	2.15	1.85	0.104	2.29	3.46	0.009	1.93	1.91	0.104
**E20-E56**	2.25	-0.42	0.661	2.23	3.11	0.028	2.34	3.43	0.018
**E20-E59**	2.18	1.57	0.282	2.29	3.18	0.006	2.11	1.57	0.282
**E22-E31**	2.26	1.12	0.287	2.30	4.62	0.003	2.27	2.25	0.104
**E22-E42**	2.04	1.56	0.145	2.33	4.37	0.003	2.11	1.88	0.142
**E22-E47**	2.12	1.59	0.143	2.30	4.61	0.003	2.02	2.23	0.072
**E22-E50**	2.18	3.45	0.006	2.16	2.85	0.034	2.02	0.45	0.704
**E22-E51**	2.17	2.46	0.050	2.35	4.75	0.003	2.13	2.41	0.050
**E22-E52**	2.25	2.55	0.046	2.25	4.14	0.009	2.00	2.20	0.066
**E22-E56**	2.19	1.41	0.180	2.22	4.31	0.003	2.14	2.16	0.090
**E22-E58**	2.26	2.52	0.029	2.27	4.05	0.003	1.95	2.71	0.024
**E22-E59**	2.09	1.33	0.238	2.32	3.59	0.024	2.19	3.11	0.026
**E22-E60**	2.22	1.05	0.341	2.25	3.62	0.024	2.17	2.63	0.044
**E22-E108**	2.15	2.92	0.036	2.27	3.06	0.036	2.04	0.17	0.872
**E22-E122**	2.14	3.90	0.006	2.28	2.63	0.062	2.19	-0.04	0.969
**E23-E42**	2.14	1.78	0.202	2.33	6.35	0.003	2.03	1.53	0.202
**E23-E47**	2.10	1.55	0.146	2.28	5.42	0.003	2.15	2.48	0.052
**E23-E51**	2.21	2.48	0.050	2.27	5.76	0.003	2.10	2.51	0.046
**E23-E52**	2.05	2.16	0.084	2.29	5.39	0.003	2.04	1.92	0.084
**E23-E56**	2.22	1.15	0.266	2.31	5.00	0.003	2.15	2.60	0.028
**E23-E58**	2.22	2.58	0.023	2.19	4.91	0.003	2.10	3.33	0.003
**E23-E59**	2.03	1.85	0.076	2.30	4.15	0.006	2.18	2.54	0.032

**E23-E60**	2.02	1.67	0.125	2.36	4.38	0.003	2.11	2.07	0.110
**E23-E65**	2.16	3.22	0.016	2.20	3.55	0.009	2.11	1.72	0.120
**E23-E66**	2.19	2.52	0.048	2.32	3.69	0.009	1.99	1.63	0.143
**E24-E42**	2.16	1.66	0.206	2.37	4.36	0.003	2.17	1.44	0.206
**E24-E47**	2.07	1.77	0.178	2.24	4.15	0.006	2.14	1.41	0.194
**E24-E51**	2.17	1.86	0.076	2.34	4.93	0.003	2.21	2.64	0.044
**E24-E52**	2.15	2.27	0.084	2.39	4.22	0.006	2.13	1.80	0.101
**E24-E58**	2.25	1.61	0.146	2.24	3.35	0.024	2.06	2.44	0.046
**E24-E59**	2.13	1.59	0.153	2.42	3.94	0.003	2.32	2.57	0.054
**E24-E60**	2.14	1.82	0.186	2.34	3.69	0.003	2.09	1.49	0.186
**E24-E65**	2.12	2.61	0.047	2.39	2.92	0.057	2.33	1.40	0.201
**E24-E66**	2.10	1.78	0.208	2.33	3.17	0.012	2.16	1.58	0.208
**E26-E47**	2.13	1.79	0.192	2.32	4.10	0.003	2.07	1.18	0.278
**E26-E51**	2.12	2.08	0.106	2.42	5.61	0.003	2.12	2.00	0.106
**E26-E52**	2.10	2.11	0.098	2.28	5.63	0.003	2.09	1.53	0.159
**E26-E53**	2.16	1.24	0.256	2.31	4.53	0.003	2.12	1.65	0.256
**E26-E58**	2.35	4.48	0.003	2.29	5.04	0.003	2.17	2.06	0.061
**E26-E59**	1.92	1.80	0.124	2.37	4.64	0.003	2.17	1.97	0.124
**E26-E60**	1.97	1.73	0.156	2.36	4.43	0.003	2.15	1.89	0.156
**E26-E65**	2.16	3.67	0.020	2.32	3.92	0.009	2.31	1.78	0.107
**E26-E66**	2.13	2.04	0.122	2.26	3.50	0.012	2.24	1.51	0.161
**E26-E67**	2.12	1.58	0.156	2.29	3.94	0.009	2.19	1.97	0.156
**E26-E72**	2.14	1.19	0.251	2.36	3.54	0.015	2.14	2.07	0.132
**E27-E51**	2.08	1.39	0.170	2.33	3.73	0.015	2.24	2.47	0.064
**E27-E52**	2.11	1.75	0.194	2.33	3.71	0.012	2.18	1.66	0.194
**E27-E59**	2.11	1.65	0.210	2.41	3.53	0.012	2.18	1.79	0.210
**E27-E60**	2.08	1.68	0.224	2.36	3.78	0.003	2.05	1.44	0.224
**E27-E66**	2.03	1.89	0.142	2.44	3.23	0.015	2.08	1.35	0.232
**E28-E52**	2.17	1.49	0.137	2.31	3.45	0.027	2.27	2.21	0.118
**E28-E59**	2.08	1.53	0.143	2.26	3.62	0.018	2.29	2.31	0.094
**E28-E60**	2.09	1.68	0.196	2.36	4.08	0.006	2.18	1.83	0.196
**E28-E66**	2.11	1.98	0.128	2.32	3.57	0.018	2.25	1.56	0.158
**E29-E66**	2.03	0.91	0.378	2.31	3.09	0.018	2.30	2.54	0.078
**E31-E80**	2.28	2.94	0.040	2.34	0.98	0.343	2.25	-3.05	0.036
**E31-E104**	2.35	2.79	0.044	2.30	1.36	0.196	2.20	-2.77	0.042
**E33-E59**	2.09	2.15	0.082	2.33	3.61	0.009	2.21	0.67	0.523
**E33-E60**	2.14	1.95	0.118	2.32	3.98	0.003	2.27	1.11	0.323
**E33-E66**	2.08	2.62	0.044	2.35	3.18	0.054	2.05	0.58	0.609
**E33-E67**	2.04	2.19	0.070	2.35	4.42	0.003	2.19	1.25	0.264
**E33-E70**	2.16	2.69	0.043	2.24	2.91	0.051	2.32	0.56	0.570
**E33-E71**	2.15	2.50	0.044	2.33	3.70	0.009	2.11	0.63	0.567
**E34-E60**	2.16	2.01	0.122	2.32	3.83	0.012	2.02	1.00	0.386
**E34-E66**	2.06	1.97	0.128	2.29	3.42	0.033	2.17	1.26	0.255
**E34-E71**	2.16	1.90	0.136	2.21	3.57	0.006	2.05	0.92	0.411
**E35-E66**	2.11	1.49	0.244	2.29	3.65	0.015	2.29	1.68	0.244
**E36-E59**	2.20	1.21	0.243	2.25	4.16	0.006	2.30	2.83	0.054
**E36-E60**	2.06	1.66	0.178	2.27	3.77	0.012	2.11	1.78	0.178
**E36-E66**	2.12	0.85	0.421	2.30	4.37	0.006	2.21	2.81	0.052
**E36-E70**	2.15	1.25	0.225	2.37	4.08	0.003	2.11	2.51	0.060

**E39-E67**	2.16	1.97	0.148	2.27	4.66	0.003	2.29	1.37	0.200
**E39-E71**	2.11	1.84	0.178	2.22	5.05	0.003	2.25	1.37	0.187
**E39-E72**	2.10	1.84	0.194	2.28	3.90	0.012	2.09	1.00	0.294
**E42-E75**	2.14	2.09	0.116	2.39	3.24	0.036	2.24	0.77	0.428
**E42-E82**	2.24	3.08	0.021	2.33	3.38	0.021	2.04	-1.02	0.303
**E42-E83**	2.11	2.73	0.038	2.27	4.00	0.003	2.17	0.85	0.424
**E42-E89**	2.27	2.92	0.039	2.24	2.76	0.046	2.21	-0.65	0.509
**E42-E90**	2.16	2.04	0.128	2.32	3.92	0.003	2.21	0.82	0.417
**E42-E124**	2.31	2.13	0.146	2.17	3.44	0.012	2.10	0.86	0.432
**E45-E60**	2.08	2.61	0.049	2.28	2.47	0.069	2.12	-0.68	0.526
**E45-E67**	2.07	2.69	0.048	2.28	2.77	0.027	2.23	0.66	0.535
**E50-E60**	2.28	3.33	0.012	2.41	2.76	0.056	2.05	-0.67	0.501
**E52-E60**	2.21	2.82	0.022	2.39	3.72	0.015	2.25	0.41	0.713
**E52-E66**	2.14	1.32	0.192	2.22	3.75	0.018	2.25	2.26	0.094
**E52-E67**	2.05	2.69	0.016	2.20	3.35	0.006	2.22	0.07	0.914
**E52-E71**	2.17	2.80	0.018	2.29	3.71	0.006	1.88	0.97	0.415
**E52-E75**	2.15	2.48	0.048	2.03	2.86	0.009	2.28	0.28	0.774
**E52-E82**	2.24	2.87	0.027	2.05	2.19	0.066	2.14	-1.13	0.246
**E52-E83**	2.06	3.03	0.014	2.03	3.02	0.006	2.17	-0.14	0.910
**E52-E90**	2.14	2.23	0.078	2.38	2.95	0.003	2.27	0.47	0.632
**E52-E104**	2.20	3.09	0.021	2.18	2.05	0.104	2.04	-2.02	0.104
**E53-E66**	2.22	0.00	0.998	2.26	2.69	0.038	2.16	4.07	0.003
**E53-E71**	2.21	1.07	0.302	2.28	3.38	0.009	2.27	2.44	0.078
**E53-E75**	2.21	2.70	0.042	2.23	3.07	0.042	2.17	-0.05	0.967
**E53-E82**	2.20	2.73	0.050	2.28	2.72	0.060	2.13	-0.76	0.431
**E53-E83**	2.09	3.36	0.016	2.42	4.95	0.003	2.12	1.11	0.294
**E53-E87**	1.95	2.61	0.027	2.10	1.18	0.272	2.02	-2.35	0.054
**E53-E90**	2.04	1.93	0.120	2.53	3.22	0.003	2.20	1.35	0.211
**E53-E101**	2.18	2.77	0.045	2.30	2.33	0.094	2.16	-0.89	0.369
**E54-E66**	2.14	0.92	0.409	2.33	3.43	0.020	2.29	3.93	0.003
**E54-E67**	2.21	1.93	0.174	2.26	4.26	0.006	2.22	1.16	0.296
**E54-E80**	2.28	2.99	0.049	2.22	1.36	0.221	2.23	-2.33	0.072
**E54-E83**	2.12	2.91	0.046	2.42	2.94	0.066	2.16	-0.10	0.914
**E54-E107**	2.30	3.44	0.021	2.40	3.42	0.021	2.07	-0.26	0.807
**E55-E98**	2.06	2.56	0.018	2.17	2.10	0.116	2.08	-0.89	0.393
**E55-E102**	2.30	3.38	0.027	2.35	2.99	0.050	2.10	-0.40	0.695
**E55-E103**	2.41	3.01	0.049	2.39	2.32	0.102	2.14	-1.27	0.218
**E55-E104**	2.35	2.97	0.027	2.25	1.20	0.250	2.17	-2.49	0.056
**E56-E60**	2.20	3.06	0.020	2.31	3.90	0.012	2.09	0.24	0.810
**E56-E65**	2.13	1.39	0.187	2.19	4.09	0.009	2.23	2.44	0.074
**E56-E70**	2.09	1.30	0.193	2.40	4.12	0.012	2.34	2.09	0.150
**E56-E73**	2.09	1.87	0.170	2.36	3.47	0.006	2.22	1.16	0.280
**E56-E112**	2.32	0.06	0.971	2.28	3.73	0.015	2.35	2.80	0.036
**E59-E66**	2.08	0.83	0.443	2.28	3.94	0.003	2.34	2.93	0.034
**E59-E83**	2.17	2.78	0.042	2.33	3.00	0.042	2.15	0.25	0.794
**E60-E67**	2.44	2.96	0.044	1.98	2.60	0.018	2.17	-0.43	0.693
**E60-E71**	2.29	2.84	0.042	2.32	3.17	0.042	2.24	-0.34	0.752
**E60-E72**	2.45	3.23	0.042	2.17	1.77	0.105	2.44	-2.71	0.060
**E60-E75**	2.18	2.65	0.032	2.32	3.45	0.009	2.25	0.22	0.822

**E60-E82**	2.22	3.27	0.021	2.21	2.83	0.034	2.20	-1.01	0.309
**E60-E83**	2.35	4.02	0.003	2.32	4.24	0.004	2.19	0.23	0.803
**E60-E101**	2.18	2.94	0.047	2.28	2.44	0.062	2.29	-0.45	0.663
**E61-E83**	2.30	3.37	0.021	2.25	3.49	0.024	2.20	0.20	0.834
**E61-E104**	2.18	2.85	0.049	2.23	2.12	0.120	2.09	-1.12	0.301
**E61-E107**	2.21	2.95	0.026	2.35	3.07	0.024	2.12	-0.10	0.940
**E62-E76**	2.32	1.89	0.168	2.24	3.74	0.009	2.39	1.29	0.226
**E62-E83**	2.27	3.01	0.033	2.34	3.64	0.033	2.11	0.58	0.560
**E62-E102**	2.23	2.86	0.027	2.28	2.24	0.108	2.23	-0.90	0.357
**E62-E104**	2.13	2.91	0.027	1.70	1.75	0.062	2.16	-2.01	0.072
**E62-E107**	2.29	3.54	0.008	2.33	3.38	0.003	2.17	-0.48	0.655
**E65-E66**	2.27	3.77	0.003	2.02	1.71	0.218	2.25	-1.31	0.218
**E65-E71**	2.43	4.09	0.024	2.21	1.29	0.314	2.22	-1.60	0.314
**E65-E83**	2.34	2.81	0.047	2.38	2.71	0.060	2.37	0.41	0.680
**E67-E76**	2.35	3.01	0.048	2.36	2.62	0.066	2.25	-0.61	0.547
**E67-E83**	2.39	6.21	0.003	2.37	3.93	0.012	2.23	0.43	0.694
**E67-E89**	2.24	3.82	0.009	2.30	2.50	0.074	2.29	0.20	0.824
**E67-E94**	2.18	3.41	0.010	2.18	3.92	0.003	2.16	-0.26	0.778
**E68-E74**	2.39	3.96	0.021	2.28	4.11	0.021	2.32	-0.55	0.551
**E68-E81**	2.20	2.08	0.116	2.37	3.55	0.021	2.36	0.71	0.501
**E68-E83**	2.44	3.15	0.040	2.39	2.16	0.150	2.19	-0.24	0.814
**E68-E101**	2.18	4.22	0.012	2.54	2.90	0.058	2.23	-0.63	0.517
**E70-E73**	2.26	3.44	0.021	2.10	2.21	0.084	2.23	-1.82	0.091
**E70-E83**	2.24	3.20	0.044	2.35	2.79	0.054	2.25	-1.18	0.245
**E71-E83**	2.24	4.23	0.003	2.38	3.43	0.016	2.26	-0.10	0.927
**E72-E83**	2.21	3.52	0.006	2.28	3.66	0.008	2.23	0.36	0.717
**E72-E98**	2.10	2.40	0.048	1.99	1.08	0.290	2.15	-3.57	0.006
**E72-E99**	2.13	3.01	0.030	1.93	2.13	0.066	2.21	-1.36	0.202
**E72-E107**	2.16	2.83	0.018	2.02	3.27	0.003	2.10	-0.73	0.486
**E73-E74**	2.25	4.19	0.010	2.18	0.71	0.453	2.33	-3.38	0.006
**E73-E98**	2.22	2.69	0.038	2.25	0.15	0.890	2.15	-3.28	0.030
**E74-E98**	2.22	2.82	0.042	2.32	-0.21	0.792	2.16	-3.80	0.012
**E74-E110**	2.20	2.89	0.047	2.15	0.58	0.569	2.18	-2.50	0.057
**E75-E76**	2.36	3.41	0.033	2.25	0.90	0.383	2.33	-2.89	0.033
**E75-E83**	2.38	3.44	0.024	2.45	2.56	0.082	2.17	-1.42	0.180
**E76-E81**	2.33	2.46	0.080	2.24	-0.27	0.777	2.24	-5.41	0.003
**E76-E83**	2.34	3.19	0.032	2.28	3.28	0.027	2.22	-0.20	0.854
**E77-E81**	2.27	2.71	0.042	2.23	0.13	0.890	2.12	-4.12	0.006
**E77-E82**	2.30	2.90	0.050	2.28	1.29	0.196	2.10	-2.57	0.060
**E77-E84**	2.34	3.30	0.030	2.35	2.84	0.042	2.19	-0.15	0.889
**E77-E85**	2.27	2.80	0.038	2.34	3.16	0.030	2.31	0.33	0.758
**E78-E85**	2.28	2.39	0.086	2.42	4.09	0.012	2.07	1.51	0.151
**E78-E98**	2.10	2.77	0.049	2.17	1.35	0.201	2.20	-2.36	0.068
**E80-E102**	2.31	2.03	0.138	2.23	3.59	0.021	2.11	1.15	0.279
**E80-E103**	2.30	2.51	0.072	2.29	3.74	0.009	2.13	0.11	0.931
**E80-E105**	2.33	2.71	0.046	2.17	3.56	0.003	2.28	0.23	0.820
**E81-E82**	2.18	3.74	0.006	2.20	1.24	0.244	2.14	-1.95	0.144
**E81-E109**	2.15	2.10	0.116	2.19	-0.73	0.467	2.11	-3.72	0.003
**E87-E103**	2.12	3.25	0.015	2.15	2.72	0.015	2.28	-0.66	0.500

**E94-E98**	2.21	-2.02	0.166	2.16	-3.08	0.042	2.21	-1.58	0.166
**E97-E107**	2.37	2.87	0.038	2.30	1.31	0.253	2.08	-2.67	0.033
**E108-E115**	2.05	3.10	0.024	2.27	2.08	0.118	2.27	-0.83	0.414

Table S15. Results of post-hoc analyses are presented for those electrode couples (i.e. connectivities), showing a significant phase-effect in theta band. For each couple and comparison (i.e. post-SNB vs post-SMB, post-SNB vs baseline and post-SMB vs baseline) three statistics are presented: $|t_{0.05}|$ indicates the two-sided significance threshold (at $p < 0.05$) derived by the permutation test on t-statistic (1000 permutations), *t-value*, the t-statistics of the paired t-test, and p_{BH} the test significance after Bonferroni-Holm correction.

Alpha

**electrodes couples**	**post-SNB vs post-SMB**			**post-SNB vs baseline**			**post-SMB vs baseline**		
	**$\|t_{0.05}\|$**	**t-value**	**p_{BH}**	**$\|t_{0.05}\|$**	**t-value**	**p_{BH}**	**$\|t_{0.05}\|$**	**t-value**	**p_{BH}**
**E3-E19**	2.28	-3.73	0.012	2.17	-3.19	0.022	2.19	0.27	0.776
**E4-E36**	2.19	-1.36	0.258	2.18	1.63	0.258	2.34	5.11	0.003
**E9-E26**	2.26	-0.46	0.664	2.36	-3.47	0.018	2.03	-2.55	0.030
**E11-E19**	2.32	-4.17	0.018	2.26	-2.11	0.114	2.09	1.43	0.194
**E30-E102**	2.25	-4.49	0.003	2.25	-2.67	0.036	2.17	-0.74	0.447
**E33-E95**	2.23	0.43	0.652	2.25	-4.05	0.009	2.33	-3.93	0.010
**E34-E95**	2.25	-0.91	0.377	2.17	-3.77	0.009	2.32	-2.87	0.054
**E37-E41**	2.24	0.64	0.539	2.07	-2.62	0.026	2.25	-4.28	0.003
**E39-E95**	2.28	1.03	0.298	2.22	-2.25	0.088	2.29	-4.20	0.021
**E46-E102**	2.22	-2.34	0.092	2.22	-3.38	0.024	2.20	-1.45	0.179
**E50-E102**	2.37	-2.48	0.082	2.32	-4.76	0.003	2.41	-1.56	0.153
**E59-E103**	2.14	-0.03	0.963	2.31	2.66	0.058	2.29	3.39	0.003
**E64-E81**	2.33	-4.07	0.006	2.24	-3.13	0.034	2.20	1.21	0.256
**E99-E107**	2.31	-3.00	0.024	2.28	-3.35	0.024	2.21	-0.21	0.800
**E115-E122**	2.34	-3.12	0.036	2.27	-2.94	0.042	2.07	0.10	0.919

Table S16. Results of post-hoc analyses are presented for those electrode couples (i.e. connectivities), showing a significant phase-effect in alpha band. For each couple and comparison (i.e. post-SNB vs post-SMB, post-SNB vs baseline and post-SMB vs baseline) three statistics are presented: $|t_{0.05}|$ indicates the two-sided significance threshold (at $p < 0.05$) derived by the permutation test on t-statistic (1000 permutations), *t-value*, the t-statistics of the paired t-test, and p_{BH} the test significance after Bonferroni-Holm correction.

Low-Beta

**electrodes couples**	**post-SNB vs post-SMB**			**post-SNB vs baseline**			**post-SMB vs baseline**		
	**$\|t_{0.05}\|$**	**t-value**	**p_{BH}**	**$\|t_{0.05}\|$**	**t-value**	**p_{BH}**	**$\|t_{0.05}\|$**	**t-value**	**p_{BH}**
**E6-E80**	2.12	3.01	0.016	2.30	3.46	0.003	2.18	-0.55	0.591
**E15-E65**	2.15	-0.06	0.964	2.22	3.11	0.015	2.27	3.52	0.015
**E36-E99**	2.26	-2.68	0.038	2.22	0.23	0.819	2.35	4.32	0.009
**E40-E99**	2.22	-3.31	0.010	2.11	0.79	0.478	2.41	5.27	0.003
**E41-E99**	2.38	-3.06	0.009	2.30	0.79	0.461	2.40	4.11	0.009
**E45-E99**	2.27	-2.32	0.092	2.29	1.01	0.334	2.29	4.45	0.003
**E46-E99**	2.30	-3.01	0.030	2.32	0.07	0.933	2.35	3.95	0.018
**E77-E106**	2.13	1.47	0.176	2.29	4.82	0.003	2.35	2.13	0.134

Table S17. Results of post-hoc analyses are presented for those electrode couples (i.e. connectivities), showing a significant phase-effect in low-beta band. For each couple and

comparison (i.e. post-SNB vs post-SMB, post-SNB vs baseline and post-SMB vs baseline) three statistics are presented: $|t_{0.05}|$ indicates the two-sided significance threshold (at $p < 0.05$) derived by the permutation test on t-statistic (1000 permutations), *t-value*, the t-statistics of the paired t-test, and p_{BH} the test significance after Bonferroni-Holm correction.

High-Beta

**electrodes couples**	**post-SNB vs post-SMB**			**post-SNB vs baseline**			**post-SMB vs baseline**		
	$\|t_{0.05}\|$	**t-value**	p_{BH}	$\|t_{0.05}\|$	**t-value**	p_{BH}	$\|t_{0.05}\|$	**t-value**	p_{BH}
**E2-E61**	2.17	1.25	0.230	2.29	3.04	0.045	2.25	1.97	0.126
**E2-E67**	2.28	1.82	0.178	2.26	3.09	0.042	2.12	1.35	0.206
**E2-E95**	2.23	1.95	0.172	2.10	2.66	0.042	2.26	0.98	0.351
**E2-E101**	2.17	2.77	0.030	2.14	3.59	0.018	2.24	0.76	0.440
**E2-E108**	2.30	2.90	0.048	2.16	2.44	0.062	2.18	-0.45	0.673
**E2-E115**	1.95	2.55	0.024	2.23	2.26	0.094	2.27	-0.43	0.699
**E3-E50**	2.17	0.69	0.478	2.19	2.96	0.027	2.14	2.08	0.110
**E3-E55**	2.19	0.64	0.522	2.17	3.76	0.018	2.17	2.07	0.128
**E3-E58**	2.17	0.75	0.463	2.28	3.02	0.021	2.34	2.32	0.100
**E3-E60**	2.06	0.65	0.533	2.26	3.28	0.012	2.35	2.62	0.062
**E3-E61**	2.00	1.30	0.230	2.31	3.87	0.009	2.28	2.08	0.134
**E3-E62**	2.16	1.47	0.158	2.20	3.39	0.024	2.15	2.18	0.094
**E3-E63**	2.24	2.68	0.048	2.10	3.53	0.003	2.18	1.53	0.135
**E3-E66**	2.05	0.94	0.377	2.30	3.53	0.024	2.22	2.80	0.038
**E3-E67**	2.08	1.45	0.174	2.31	4.19	0.009	2.23	1.96	0.174
**E3-E72**	2.13	1.21	0.245	2.18	4.19	0.003	2.25	1.69	0.234
**E3-E86**	2.16	2.71	0.030	2.14	2.97	0.024	2.24	1.42	0.192
**E3-E92**	2.10	2.91	0.036	2.18	2.47	0.036	2.13	0.27	0.780
**E3-E101**	2.13	1.76	0.218	2.30	3.52	0.012	2.36	1.22	0.228
**E3-E108**	2.17	2.34	0.078	2.18	3.98	0.006	2.22	0.43	0.672
**E3-E109**	2.19	1.76	0.194	2.28	4.03	0.018	2.11	1.37	0.217
**E3-E110**	1.97	2.67	0.030	2.18	2.59	0.042	2.16	0.42	0.699
**E3-E115**	2.20	2.19	0.104	2.13	3.29	0.021	2.14	0.48	0.651
**E3-E123**	2.26	3.78	0.003	2.27	2.78	0.032	1.74	-1.74	0.051
**E4-E46**	2.07	0.37	0.711	2.33	4.10	0.003	2.22	2.23	0.096
**E4-E52**	2.23	1.01	0.317	2.23	3.39	0.006	2.28	2.02	0.142
**E4-E53**	2.24	1.14	0.270	2.29	3.06	0.015	2.28	1.96	0.174
**E4-E54**	2.29	1.44	0.191	2.33	3.49	0.012	2.44	2.05	0.152
**E4-E55**	2.27	1.13	0.273	2.13	4.35	0.009	2.29	1.81	0.218
**E4-E56**	2.13	0.54	0.575	2.39	3.27	0.021	2.19	2.54	0.052
**E4-E58**	2.11	1.46	0.167	2.27	3.20	0.012	2.30	1.96	0.164
**E4-E60**	2.13	0.59	0.562	2.25	3.35	0.006	2.31	2.78	0.044
**E4-E61**	2.17	1.43	0.194	2.40	4.04	0.009	2.26	2.93	0.036
**E4-E62**	2.27	2.27	0.100	2.25	3.79	0.012	2.31	0.64	0.538
**E4-E63**	2.12	2.67	0.028	2.10	3.15	0.003	2.22	0.89	0.369
**E4-E66**	2.18	1.22	0.249	2.25	3.35	0.027	2.24	2.58	0.044
**E4-E67**	2.16	1.72	0.116	2.35	4.45	0.009	2.20	2.09	0.112
**E4-E72**	2.18	1.96	0.150	2.14	4.56	0.003	2.27	0.69	0.498
**E4-E79**	2.32	2.85	0.047	2.29	2.67	0.063	2.20	0.09	0.920
**E4-E86**	2.09	3.15	0.015	2.13	2.66	0.034	2.25	0.64	0.519

**E4-E91**	2.00	2.54	0.044	2.23	2.92	0.048	2.27	0.98	0.327
**E4-E101**	2.23	2.04	0.124	2.31	3.39	0.021	2.32	1.17	0.260
**E5-E58**	2.23	1.22	0.257	2.32	3.90	0.018	2.38	1.84	0.208
**E5-E61**	2.08	1.34	0.209	2.23	3.05	0.021	2.22	2.63	0.054
**E5-E62**	2.24	2.12	0.124	2.26	3.27	0.036	2.29	0.46	0.630
**E5-E63**	2.30	3.24	0.003	2.23	4.10	0.003	2.23	0.34	0.758
**E5-E66**	2.05	0.87	0.437	2.24	3.08	0.033	2.31	2.58	0.048
**E5-E67**	2.03	1.58	0.137	2.28	3.66	0.015	2.11	2.50	0.070
**E5-E106**	2.37	2.83	0.046	2.26	3.04	0.021	2.10	-0.64	0.546
**E5-E115**	2.26	3.84	0.006	2.14	3.82	0.014	2.18	0.38	0.689
**E6-E58**	2.15	1.20	0.268	2.27	3.60	0.021	2.28	2.33	0.090
**E6-E63**	2.26	2.83	0.042	2.24	2.99	0.042	2.15	0.57	0.581
**E6-E72**	2.16	2.04	0.118	2.15	3.55	0.015	2.15	0.44	0.647
**E6-E101**	2.30	2.26	0.104	2.13	2.88	0.033	2.31	0.30	0.772
**E6-E106**	2.31	3.93	0.006	2.11	1.75	0.117	2.21	-2.12	0.116
**E6-E108**	2.21	1.60	0.274	2.14	4.06	0.003	2.08	1.26	0.274
**E6-E109**	2.08	1.60	0.234	2.22	3.37	0.018	2.29	1.75	0.234
**E6-E115**	2.26	2.82	0.034	2.21	3.60	0.015	2.34	-0.22	0.838
**E9-E50**	2.28	0.72	0.507	2.27	3.45	0.006	2.19	2.30	0.068
**E9-E51**	1.98	-0.04	0.977	2.14	2.77	0.024	2.25	2.92	0.038
**E9-E52**	2.20	0.38	0.702	2.22	2.61	0.039	2.32	2.64	0.050
**E9-E54**	2.36	1.51	0.168	2.25	2.96	0.042	2.28	1.92	0.168
**E9-E55**	2.27	1.32	0.198	2.13	3.90	0.009	2.26	2.01	0.140
**E9-E58**	2.07	0.26	0.794	2.35	3.28	0.021	2.35	3.22	0.022
**E9-E59**	2.30	0.28	0.759	2.27	3.24	0.018	2.38	2.93	0.036
**E9-E60**	2.21	0.37	0.701	2.20	2.98	0.030	2.36	3.05	0.030
**E9-E61**	2.17	1.60	0.141	2.31	3.53	0.027	2.26	2.68	0.052
**E9-E62**	2.30	1.87	0.170	2.21	3.15	0.039	2.26	1.45	0.170
**E9-E66**	2.15	1.12	0.272	2.24	3.14	0.036	2.23	2.41	0.062
**E9-E67**	2.21	1.91	0.086	2.27	3.77	0.015	2.16	2.38	0.056
**E9-E72**	2.14	1.51	0.310	2.22	3.64	0.015	2.17	1.44	0.310
**E9-E101**	2.20	1.91	0.172	2.20	3.99	0.009	2.23	0.93	0.322
**E9-E106**	2.42	2.91	0.044	2.25	3.28	0.018	2.15	-0.04	0.967
**E9-E115**	2.15	2.68	0.044	2.07	2.98	0.033	2.08	1.42	0.175
**E9-E122**	2.10	0.58	0.566	2.22	3.16	0.024	2.35	3.14	0.030
**E9-E124**	2.19	3.25	0.027	2.18	1.38	0.246	2.05	-1.59	0.246
**E10-E46**	2.08	-0.10	0.927	2.40	3.51	0.003	2.21	2.78	0.046
**E10-E53**	2.28	1.10	0.275	2.16	2.94	0.048	2.24	2.15	0.118
**E10-E54**	2.19	1.39	0.204	2.27	3.17	0.027	2.33	1.90	0.182
**E10-E55**	2.27	1.29	0.294	2.17	3.79	0.009	2.32	1.58	0.294
**E10-E58**	2.08	0.50	0.639	2.24	3.34	0.015	2.34	2.61	0.060
**E10-E59**	2.30	0.28	0.758	2.25	3.12	0.024	2.41	2.99	0.036
**E10-E60**	2.24	0.42	0.667	2.23	3.26	0.015	2.34	3.29	0.016
**E10-E61**	2.12	1.48	0.167	2.28	3.91	0.012	2.26	2.55	0.050
**E10-E62**	2.19	2.02	0.130	2.16	3.32	0.012	2.24	0.80	0.430
**E10-E63**	2.31	1.83	0.202	2.15	2.97	0.006	2.26	1.56	0.202
**E10-E66**	2.15	1.06	0.308	2.27	3.32	0.024	2.24	2.93	0.024
**E10-E67**	2.17	1.84	0.098	2.33	4.24	0.003	2.13	2.13	0.098
**E10-E101**	2.26	1.60	0.284	2.17	4.28	0.003	2.20	1.42	0.284

**E10-E106**	2.40	2.84	0.042	2.25	3.12	0.027	2.07	-0.52	0.609
**E10-E115**	2.20	2.36	0.070	2.12	2.88	0.036	2.24	0.24	0.816
**E11-E31**	2.18	0.75	0.441	2.27	3.15	0.036	2.35	2.60	0.062
**E11-E46**	2.12	-0.70	0.488	2.36	3.70	0.003	2.27	3.68	0.016
**E11-E50**	2.15	0.11	0.911	2.33	2.63	0.058	2.21	2.91	0.033
**E11-E51**	2.09	-0.57	0.567	2.18	2.68	0.033	2.31	3.28	0.033
**E11-E52**	2.36	-0.12	0.884	2.35	2.56	0.058	2.33	3.18	0.042
**E11-E54**	2.30	0.98	0.319	2.25	3.35	0.024	2.39	2.46	0.090
**E11-E56**	2.19	0.52	0.607	2.18	3.43	0.015	2.15	2.60	0.032
**E11-E58**	2.40	0.02	0.991	2.23	3.72	0.015	2.26	3.63	0.018
**E11-E59**	2.20	0.04	0.965	2.21	3.10	0.020	2.27	3.49	0.012
**E11-E60**	2.25	0.15	0.881	2.23	3.61	0.003	2.32	4.70	0.004
**E11-E61**	2.26	1.31	0.210	2.26	3.91	0.006	2.33	2.52	0.062
**E11-E63**	2.28	1.05	0.321	2.21	2.97	0.030	2.19	2.03	0.130
**E11-E66**	2.39	1.08	0.271	2.24	3.16	0.030	2.30	2.79	0.032
**E11-E67**	2.19	1.66	0.116	2.34	4.32	0.003	2.27	2.75	0.034
**E11-E83**	2.03	2.24	0.076	2.22	3.61	0.018	2.12	-0.49	0.631
**E11-E115**	2.23	2.71	0.044	2.11	2.96	0.027	2.19	1.12	0.272
**E12-E50**	2.29	1.96	0.116	2.35	3.48	0.018	2.20	2.12	0.116
**E12-E54**	2.25	1.08	0.318	2.31	3.29	0.039	2.34	2.11	0.128
**E12-E56**	2.28	0.79	0.431	2.18	3.36	0.006	2.23	3.91	0.006
**E12-E58**	2.31	0.26	0.798	2.22	3.15	0.015	2.24	2.81	0.054
**E12-E60**	2.20	0.53	0.634	2.26	3.72	0.006	2.26	3.03	0.024
**E12-E61**	1.98	1.23	0.267	2.32	5.08	0.003	2.35	2.32	0.116
**E12-E65**	2.08	0.92	0.372	2.21	3.03	0.039	2.20	2.70	0.050
**E12-E66**	2.15	1.50	0.170	2.22	4.04	0.006	2.38	2.06	0.170
**E12-E67**	2.18	1.99	0.073	2.39	6.45	0.003	2.28	3.11	0.034
**E12-E70**	2.20	1.66	0.254	2.08	3.09	0.009	2.21	1.23	0.254
**E12-E83**	2.07	2.61	0.032	2.26	4.00	0.003	2.10	-0.25	0.804
**E12-E90**	2.11	2.59	0.045	2.26	3.73	0.006	2.17	0.63	0.555
**E12-E101**	2.26	1.69	0.168	2.37	3.77	0.030	2.27	1.98	0.168
**E12-E106**	2.15	2.92	0.030	2.27	2.20	0.114	2.10	-0.73	0.475
**E12-E109**	2.16	0.81	0.435	2.32	3.42	0.030	2.32	2.59	0.052
**E12-E115**	2.26	4.32	0.003	2.12	3.74	0.003	2.14	1.14	0.294
**E12-E116**	2.16	1.36	0.199	2.17	4.11	0.003	2.15	2.43	0.032
**E12-E117**	2.20	1.60	0.168	2.17	4.67	0.003	2.25	2.01	0.168
**E13-E50**	2.29	1.55	0.138	2.25	3.00	0.039	2.21	2.38	0.082
**E13-E56**	2.20	2.00	0.148	2.22	3.08	0.024	2.35	1.04	0.310
**E13-E67**	2.25	1.16	0.277	2.23	2.86	0.048	2.35	1.90	0.190
**E13-E109**	2.17	1.21	0.255	2.18	3.56	0.009	2.26	2.87	0.030
**E13-E110**	2.20	0.87	0.418	2.11	3.52	0.003	2.34	2.01	0.186
**E15-E60**	2.30	0.30	0.782	2.23	2.75	0.027	2.33	3.05	0.036
**E15-E67**	2.18	2.06	0.128	2.27	4.28	0.003	2.19	1.43	0.169
**E15-E101**	2.20	1.65	0.254	2.14	3.88	0.006	2.35	1.18	0.254
**E15-E106**	2.33	2.82	0.046	2.27	2.93	0.045	2.22	-0.36	0.710
**E16-E60**	2.22	0.52	0.615	2.27	3.15	0.012	2.30	3.03	0.038
**E16-E61**	2.25	1.89	0.188	2.35	3.41	0.018	2.34	1.37	0.206
**E16-E62**	2.19	2.29	0.082	2.15	3.14	0.021	2.24	0.00	0.999
**E16-E67**	2.17	2.63	0.042	2.31	4.69	0.003	2.25	2.07	0.066

**E16-E83**	2.11	2.59	0.032	2.35	4.24	0.009	2.11	-0.74	0.464
**E16-E90**	2.07	2.66	0.033	2.29	2.80	0.040	2.22	-0.02	0.979
**E16-E101**	2.32	1.50	0.306	2.19	3.78	0.006	2.20	1.20	0.306
**E16-E106**	2.25	2.25	0.100	2.29	3.24	0.021	2.39	-0.11	0.920
**E16-E115**	2.19	2.39	0.072	2.11	2.83	0.027	2.12	1.11	0.270
**E18-E59**	2.25	-0.18	0.847	2.19	2.13	0.120	2.40	2.93	0.045
**E18-E60**	2.27	0.33	0.756	2.30	3.10	0.003	2.35	3.20	0.024
**E18-E61**	2.23	1.55	0.284	2.34	3.61	0.018	2.33	1.52	0.284
**E18-E66**	2.24	1.28	0.218	2.24	3.02	0.015	2.25	2.39	0.076
**E18-E67**	2.17	1.92	0.114	2.29	5.81	0.003	2.30	2.25	0.114
**E18-E115**	2.30	2.60	0.046	2.16	2.94	0.033	2.26	0.75	0.481
**E19-E56**	2.14	0.68	0.479	2.11	3.94	0.012	2.31	2.40	0.076
**E19-E58**	2.21	0.38	0.693	2.22	3.45	0.015	2.26	3.69	0.015
**E19-E59**	2.21	-0.21	0.818	2.25	2.33	0.064	2.25	3.27	0.033
**E19-E60**	2.24	0.18	0.869	2.32	3.42	0.003	2.40	3.92	0.010
**E19-E61**	2.18	1.38	0.420	2.39	3.96	0.003	2.46	1.42	0.420
**E19-E66**	2.21	1.74	0.098	2.30	3.30	0.009	2.27	2.47	0.064
**E19-E67**	2.19	1.84	0.178	2.33	5.60	0.003	2.35	1.88	0.178
**E19-E115**	2.30	1.72	0.176	2.16	5.32	0.003	2.17	1.91	0.176
**E20-E50**	2.25	-0.05	0.967	2.16	2.69	0.024	2.17	2.64	0.044
**E20-E56**	2.22	1.13	0.284	2.26	3.08	0.024	2.17	2.15	0.102
**E20-E58**	2.09	-0.05	0.966	2.28	3.04	0.022	2.30	3.96	0.018
**E20-E59**	2.13	-0.15	0.885	2.31	2.63	0.054	2.32	3.67	0.003
**E20-E60**	1.89	0.83	0.511	2.36	2.99	0.033	2.31	2.99	0.033
**E20-E65**	2.08	0.73	0.488	2.19	2.89	0.018	2.32	2.75	0.034
**E20-E66**	2.16	1.19	0.268	2.19	3.31	0.018	2.30	2.91	0.026
**E20-E67**	2.34	0.92	0.366	2.39	3.46	0.018	2.30	3.09	0.022
**E20-E116**	2.14	0.69	0.524	2.20	3.11	0.042	2.30	3.17	0.042
**E20-E123**	2.10	0.79	0.450	2.20	3.53	0.012	2.30	2.76	0.046
**E22-E58**	2.06	-0.11	0.920	2.32	2.82	0.042	2.28	3.02	0.042
**E22-E59**	2.14	0.41	0.722	2.18	2.59	0.042	2.31	3.07	0.042
**E22-E60**	2.29	0.52	0.625	2.35	3.03	0.006	2.34	3.06	0.028
**E22-E66**	2.19	1.52	0.148	2.30	3.51	0.012	2.20	2.14	0.116
**E22-E67**	2.14	1.64	0.120	2.41	5.13	0.003	2.25	2.24	0.100
**E22-E70**	2.26	2.86	0.044	2.07	2.95	0.024	2.18	0.50	0.614
**E22-E78**	2.20	3.24	0.027	2.17	2.69	0.048	2.13	-0.70	0.489
**E22-E86**	2.15	2.99	0.021	2.05	2.33	0.044	2.20	-0.20	0.851
**E23-E60**	2.22	0.20	0.847	2.34	2.94	0.024	2.39	3.21	0.026
**E23-E66**	2.17	1.18	0.263	2.30	2.92	0.033	2.25	2.12	0.132
**E23-E67**	2.15	1.47	0.175	2.32	6.36	0.003	2.29	2.72	0.044
**E23-E115**	2.21	2.58	0.036	2.23	4.30	0.003	2.20	0.98	0.354
**E24-E50**	2.19	-0.37	0.722	2.16	2.68	0.027	2.09	2.95	0.027
**E24-E51**	2.10	-0.70	0.504	2.19	2.53	0.066	2.28	3.58	0.033
**E24-E56**	2.26	0.63	0.530	2.24	3.59	0.015	2.34	2.55	0.068
**E24-E58**	2.08	0.12	0.910	2.28	3.33	0.010	2.33	4.64	0.006
**E24-E59**	2.13	0.08	0.931	2.25	2.52	0.060	2.40	3.42	0.024
**E24-E60**	2.04	0.47	0.744	2.40	2.96	0.024	2.28	3.44	0.015
**E24-E66**	2.07	1.82	0.172	2.26	2.85	0.048	2.13	1.89	0.172
**E24-E67**	2.28	1.06	0.333	2.37	3.89	0.003	2.30	3.13	0.020

**E24-E123**	2.28	0.29	0.790	2.31	4.49	0.003	2.18	2.68	0.044
**E24-E124**	2.28	0.85	0.427	2.28	4.27	0.003	2.14	2.25	0.072
**E26-E58**	2.31	0.17	0.865	2.34	3.04	0.039	2.23	2.32	0.084
**E26-E67**	2.12	1.62	0.188	2.21	3.65	0.003	2.34	1.94	0.188
**E27-E58**	2.13	-0.31	0.752	2.20	2.89	0.036	2.30	4.10	0.003
**E27-E59**	2.22	0.09	0.925	2.22	2.67	0.054	2.39	3.18	0.039
**E27-E60**	2.25	0.23	0.826	2.35	2.78	0.044	2.33	3.33	0.030
**E27-E67**	2.29	0.69	0.513	2.31	3.47	0.030	2.26	3.24	0.030
**E27-E115**	2.13	1.57	0.286	2.20	3.21	0.036	2.26	1.49	0.286
**E27-E124**	2.40	1.50	0.286	2.35	3.68	0.015	2.18	1.54	0.286
**E29-E56**	2.34	2.83	0.030	2.21	2.85	0.030	2.32	0.42	0.677
**E30-E56**	2.18	3.06	0.018	2.22	3.30	0.009	2.30	0.90	0.407
**E30-E109**	2.17	1.40	0.248	2.22	3.04	0.021	2.24	1.66	0.248
**E33-E108**	2.16	0.60	0.565	2.18	3.81	0.015	2.20	2.30	0.086
**E35-E37**	2.08	2.58	0.046	2.09	2.77	0.030	2.29	-0.87	0.375
**E35-E39**	2.00	1.64	0.226	2.23	3.58	0.012	2.15	1.08	0.308
**E36-E37**	2.08	2.71	0.033	2.08	2.17	0.074	2.19	-0.56	0.569
**E36-E39**	1.88	1.68	0.210	2.20	3.92	0.003	2.11	0.96	0.363
**E37-E39**	2.06	2.92	0.003	2.27	3.35	0.008	2.12	0.11	0.917
**E37-E40**	2.18	3.62	0.006	2.26	2.60	0.050	2.11	-1.87	0.079
**E39-E41**	2.16	1.35	0.276	2.19	3.06	0.030	2.18	1.58	0.276
**E40-E42**	2.12	3.18	0.009	2.19	2.09	0.128	2.25	-0.41	0.689
**E40-E54**	2.02	2.52	0.045	2.11	2.41	0.048	2.17	-0.73	0.447
**E40-E61**	2.09	2.53	0.036	2.23	2.73	0.050	2.01	-1.08	0.311
**E42-E45**	2.18	2.63	0.048	2.33	2.60	0.064	2.32	0.95	0.349
**E42-E103**	2.36	2.88	0.042	2.40	2.64	0.060	2.24	-0.84	0.408
**E42-E109**	2.25	2.44	0.074	2.10	2.38	0.045	2.23	-0.91	0.371
**E45-E46**	2.20	2.11	0.120	2.38	5.39	0.003	2.11	1.09	0.286
**E45-E47**	2.20	2.71	0.042	2.45	3.39	0.042	2.16	1.06	0.298
**E45-E51**	2.06	3.06	0.006	2.26	3.02	0.046	2.30	0.55	0.600
**E45-E52**	2.20	3.73	0.006	2.36	3.76	0.006	2.25	0.13	0.899
**E45-E54**	2.15	3.01	0.033	2.33	2.66	0.050	2.19	-0.11	0.900
**E45-E55**	2.43	3.07	0.048	2.35	1.77	0.146	2.18	-1.95	0.146
**E45-E60**	2.19	3.09	0.021	2.32	3.05	0.021	2.19	-0.24	0.799
**E45-E61**	2.14	2.80	0.039	2.33	3.25	0.039	2.22	-0.51	0.597
**E45-E67**	2.01	1.99	0.104	2.32	3.03	0.039	2.28	0.15	0.872
**E46-E52**	1.96	2.10	0.072	2.11	2.81	0.045	2.19	-0.22	0.829
**E47-E86**	2.06	2.23	0.076	2.21	0.09	0.929	2.21	-4.17	0.003
**E50-E123**	2.16	0.86	0.406	2.14	3.08	0.027	2.15	1.95	0.152
**E51-E63**	2.30	3.39	0.045	2.30	2.63	0.070	2.15	-0.32	0.723
**E52-E103**	2.12	2.71	0.045	2.15	2.07	0.128	2.07	-1.39	0.207
**E53-E103**	2.22	2.77	0.046	2.19	1.57	0.162	2.20	-1.94	0.138
**E54-E70**	2.00	0.88	0.454	2.22	3.04	0.021	2.31	2.69	0.052
**E54-E78**	2.17	2.69	0.018	2.29	0.93	0.346	2.24	-2.62	0.062
**E56-E102**	2.30	1.40	0.236	2.13	2.94	0.039	2.40	1.72	0.236
**E56-E103**	2.22	1.94	0.144	2.17	3.17	0.012	2.36	1.73	0.144
**E56-E107**	1.98	2.44	0.024	2.12	3.65	0.009	2.10	-0.12	0.908
**E56-E110**	2.02	1.01	0.337	2.21	2.78	0.042	2.16	2.52	0.050
**E58-E111**	2.11	1.41	0.406	2.27	3.91	0.003	2.25	1.37	0.406

**E58-E122**	2.31	1.18	0.258	2.27	3.47	0.015	2.31	2.00	0.176
**E58-E123**	2.20	1.34	0.242	2.25	3.34	0.015	2.27	1.73	0.242
**E58-E124**	2.16	1.72	0.224	2.36	3.18	0.036	2.33	1.54	0.224
**E61-E70**	2.00	1.72	0.208	2.28	3.26	0.009	2.27	1.66	0.208
**E61-E124**	2.10	1.34	0.214	2.30	3.55	0.009	2.24	1.83	0.206
**E62-E115**	2.18	2.57	0.045	2.33	2.75	0.048	2.14	-0.38	0.708
**E62-E118**	2.08	1.95	0.134	2.20	3.73	0.027	2.18	0.66	0.511
**E62-E123**	2.13	1.55	0.242	2.24	4.10	0.009	2.31	1.74	0.242
**E62-E124**	2.13	2.31	0.072	2.19	4.07	0.012	2.22	0.82	0.424
**E63-E101**	2.19	2.20	0.098	2.20	2.96	0.015	2.11	1.31	0.211
**E63-E102**	2.23	2.95	0.045	2.34	2.84	0.051	2.23	0.56	0.593
**E63-E103**	2.28	4.12	0.012	2.25	3.34	0.030	2.30	0.59	0.562
**E63-E104**	2.33	3.20	0.047	2.26	2.95	0.069	2.21	0.23	0.834
**E63-E108**	2.23	2.52	0.060	2.22	3.38	0.030	2.20	1.03	0.328
**E63-E109**	2.18	3.67	0.009	2.19	3.51	0.010	2.20	1.55	0.154
**E63-E110**	2.37	2.99	0.032	2.21	3.21	0.024	2.19	1.04	0.336
**E63-E111**	2.41	3.05	0.048	2.19	2.51	0.048	2.16	0.37	0.725
**E63-E112**	2.24	2.79	0.047	2.36	3.22	0.015	2.22	0.18	0.852
**E63-E116**	2.37	3.33	0.024	2.30	2.95	0.034	2.24	0.32	0.757
**E63-E117**	2.25	3.54	0.003	2.19	3.00	0.020	2.17	0.37	0.740
**E63-E118**	2.24	2.72	0.044	2.24	3.36	0.009	2.08	0.90	0.388
**E63-E123**	2.29	3.78	0.009	2.18	3.34	0.018	2.26	0.81	0.411
**E63-E124**	2.25	3.95	0.003	2.19	3.48	0.010	2.18	0.41	0.689
**E64-E103**	2.47	3.12	0.047	2.17	1.63	0.272	2.32	-1.45	0.272
**E64-E106**	2.29	2.87	0.042	2.13	2.49	0.045	2.17	-0.16	0.889
**E64-E109**	2.27	4.24	0.003	2.17	2.47	0.052	2.17	-0.98	0.318
**E64-E110**	2.41	3.87	0.012	2.12	2.42	0.060	2.22	-0.20	0.829
**E64-E111**	2.30	2.94	0.033	2.01	2.42	0.033	2.30	0.02	0.971
**E66-E70**	2.26	2.94	0.049	2.24	2.60	0.069	2.22	0.35	0.719
**E66-E124**	2.15	1.49	0.170	2.27	3.84	0.003	2.30	2.22	0.112
**E67-E69**	2.27	2.83	0.050	2.19	3.18	0.018	2.08	-0.48	0.606
**E67-E70**	2.17	2.23	0.066	2.19	3.40	0.006	2.24	1.41	0.195
**E67-E71**	2.11	1.88	0.158	2.12	3.80	0.009	2.05	1.13	0.307
**E67-E122**	2.17	1.54	0.250	2.29	3.75	0.003	2.23	1.74	0.250
**E67-E123**	2.04	1.32	0.336	2.26	3.86	0.006	2.29	1.53	0.336
**E67-E124**	2.13	1.77	0.192	2.32	4.56	0.003	2.09	1.32	0.214
**E68-E106**	2.20	2.67	0.048	2.11	3.14	0.006	2.20	0.57	0.579
**E68-E112**	1.95	2.07	0.060	2.20	2.94	0.018	2.21	0.77	0.440
**E70-E124**	2.14	2.05	0.148	2.08	3.09	0.015	2.06	0.43	0.683
**E72-E123**	2.21	1.22	0.342	2.25	5.00	0.003	2.26	1.47	0.342
**E72-E124**	2.13	2.07	0.110	2.23	4.58	0.003	2.24	0.54	0.586
**E80-E109**	2.16	2.97	0.048	2.20	1.65	0.132	2.14	-2.25	0.092
**E80-E110**	2.13	2.89	0.046	2.18	2.28	0.078	2.26	-1.48	0.157
**E86-E117**	2.29	3.51	0.018	2.18	3.22	0.009	2.10	0.41	0.695
**E86-E118**	2.36	2.54	0.048	2.22	3.34	0.003	2.38	0.88	0.379
**E86-E123**	2.25	2.92	0.021	2.32	2.97	0.036	2.28	0.78	0.455
**E86-E124**	2.22	3.24	0.012	2.33	3.33	0.009	2.27	0.34	0.737
**E87-E103**	2.37	3.29	0.039	2.21	1.34	0.212	2.07	-2.46	0.050
**E91-E116**	2.07	1.46	0.190	2.21	3.65	0.018	2.17	1.76	0.190

**E91-E117**	2.13	1.58	0.214	2.11	3.62	0.015	2.06	1.68	0.214
**E91-E118**	2.33	2.45	0.080	2.25	3.02	0.015	2.25	0.78	0.435
**E92-E116**	2.13	2.79	0.036	2.20	2.32	0.084	2.23	-1.15	0.268
**E92-E117**	2.07	3.32	0.021	2.12	3.41	0.021	2.19	0.34	0.733
**E92-E118**	2.21	4.18	0.015	2.13	2.74	0.020	2.04	-0.29	0.745
**E92-E123**	2.15	2.85	0.036	2.21	2.75	0.036	2.22	-0.01	0.994
**E92-E124**	2.11	3.52	0.012	2.13	2.73	0.030	2.13	-0.79	0.446
**E93-E103**	2.44	2.88	0.045	2.32	0.61	0.550	2.37	-4.85	0.012
**E93-E117**	2.15	2.72	0.046	2.16	3.05	0.024	2.27	0.72	0.510
**E97-E98**	2.17	1.11	0.291	2.14	2.50	0.040	2.06	2.96	0.021
**E97-E116**	2.33	2.92	0.044	2.23	2.79	0.054	2.17	-0.19	0.855
**E97-E117**	2.34	3.24	0.028	2.18	3.25	0.024	2.04	1.10	0.336
**E97-E118**	2.18	3.72	0.021	2.13	2.83	0.021	2.07	0.61	0.544
**E97-E124**	2.29	3.72	0.024	2.18	2.26	0.080	2.01	-0.26	0.759
**E99-E122**	2.20	-1.83	0.132	2.18	2.07	0.132	2.14	3.32	0.036
**E101-E118**	2.19	1.44	0.178	2.35	3.52	0.012	2.19	1.83	0.178
**E101-E124**	2.18	1.72	0.224	2.29	3.08	0.039	2.29	1.47	0.224
**E106-E109**	2.20	3.09	0.041	2.36	2.49	0.070	2.01	-1.26	0.238
**E106-E111**	2.21	2.62	0.063	2.42	2.77	0.063	2.09	-0.85	0.415
**E108-E124**	2.16	1.46	0.340	2.19	3.88	0.009	2.06	1.25	0.340
**E109-E117**	2.18	1.53	0.145	2.31	3.66	0.018	2.17	2.18	0.098
**E109-E124**	2.27	1.08	0.301	2.31	3.53	0.024	2.30	1.94	0.166

Table S18. Results of post-hoc analyses are presented for those electrode couples (i.e. connectivities), showing a significant phase-effect in high-beta band. For each couple and comparison (i.e. post-SNB vs post-SMB, post-SNB vs baseline and post-SMB vs baseline) three statistics are presented: $|t_{0.05}|$ indicates the two-sided significance threshold (at $p < 0.05$) derived by the permutation test on t-statistic (1000 permutations), *t-value*, the t-statistics of the paired t-test, and p_{BH} the test significance after Bonferroni-Holm correction.

Gamma

**electrodes couples**	**post-SNB vs post-SMB**			**post-SNB vs baseline**			**post-SMB vs baseline**		
	**$\|t_{0.05}\|$**	**t-value**	**p_{BH}**	**$\|t_{0.05}\|$**	**t-value**	**p_{BH}**	**$\|t_{0.05}\|$**	**t-value**	**p_{BH}**
**E10-E70**	2.23	-0.06	0.934	2.34	-4.03	0.024	2.34	-2.36	0.096
**E11-E19**	2.17	-3.11	0.015	2.33	-0.34	0.742	2.36	2.22	0.126
**E15-E70**	2.27	-0.03	0.976	2.23	-3.39	0.030	2.20	-2.88	0.036
**E20-E95**	2.29	2.54	0.068	2.51	3.17	0.048	2.29	0.42	0.700
**E27-E95**	2.31	3.06	0.045	2.20	2.80	0.045	2.08	0.31	0.746
**E28-E95**	2.27	3.00	0.048	2.31	2.71	0.058	2.19	-0.81	0.461
**E29-E96**	2.13	3.00	0.024	2.26	1.89	0.196	2.13	-0.92	0.380
**E31-E80**	2.21	3.12	0.021	2.22	-0.43	0.683	2.46	-3.09	0.042
**E39-E94**	2.32	3.37	0.039	2.29	2.57	0.048	2.20	-0.49	0.643
**E39-E95**	2.31	3.24	0.042	2.19	1.77	0.254	2.23	-1.33	0.254
**E41-E101**	2.31	2.58	0.056	2.41	3.25	0.036	2.46	-0.27	0.791
**E41-E107**	2.44	3.32	0.012	2.19	2.45	0.064	2.22	-0.69	0.504
**E46-E101**	2.39	3.71	0.009	2.30	2.79	0.046	2.33	-1.10	0.308
**E46-E107**	2.28	3.20	0.021	2.14	2.82	0.021	2.14	-0.30	0.780
**E85-E91**	2.30	3.67	0.015	2.25	1.61	0.140	2.23	-2.05	0.136

Table S19. Results of post-hoc analyses are presented for those electrode couples (i.e. connectivities), showing a significant phase-effect in gamma band. For each couple and comparison (i.e. post-SNB vs post-SMB, post-SNB vs baseline and post-SMB vs baseline) three statistics are presented: $|t_{0.05}|$ indicates the two-sided significance threshold (at $p < 0.05$) derived by the permutation test on t-statistic (1000 permutations), *t-value,* the t-statistics of the paired t-test, and p_{BH} the test significance after Bonferroni-Holm correction.

Please note that in the post-hoc tables, the statistics of those electrodes showing both a significant phase-effect and at least one significant post-hoc comparison are reported.

SM6. Phase Amplitude Coupling

Envelope to Signal Correlation, ESC (Phase-Amplitude Coupling).

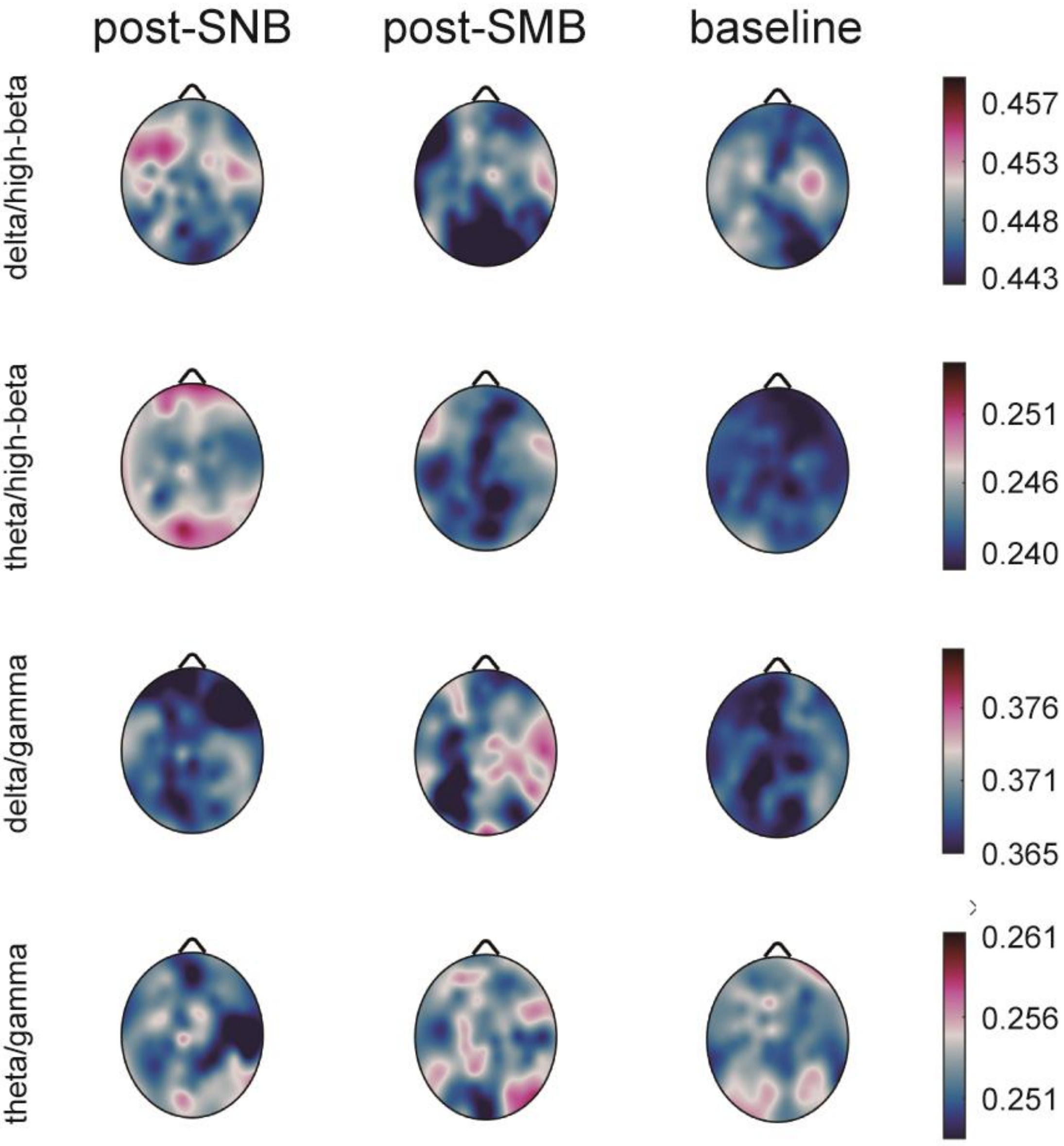

Figure S13. Average ESC scalp maps are presented for each couple of bands and phase. For visual clarity, each band has its own colorbar.

For each couple of bands and channel (ESC), a repeated-measures ANOVA with *phase* as a three-levels within factor (post-SNB, post-SMB, baseline) was conducted. For each band, *phase* significance at each channel was assessed using a single threshold premutation test for the maximum F-statistic (1000 permutations).

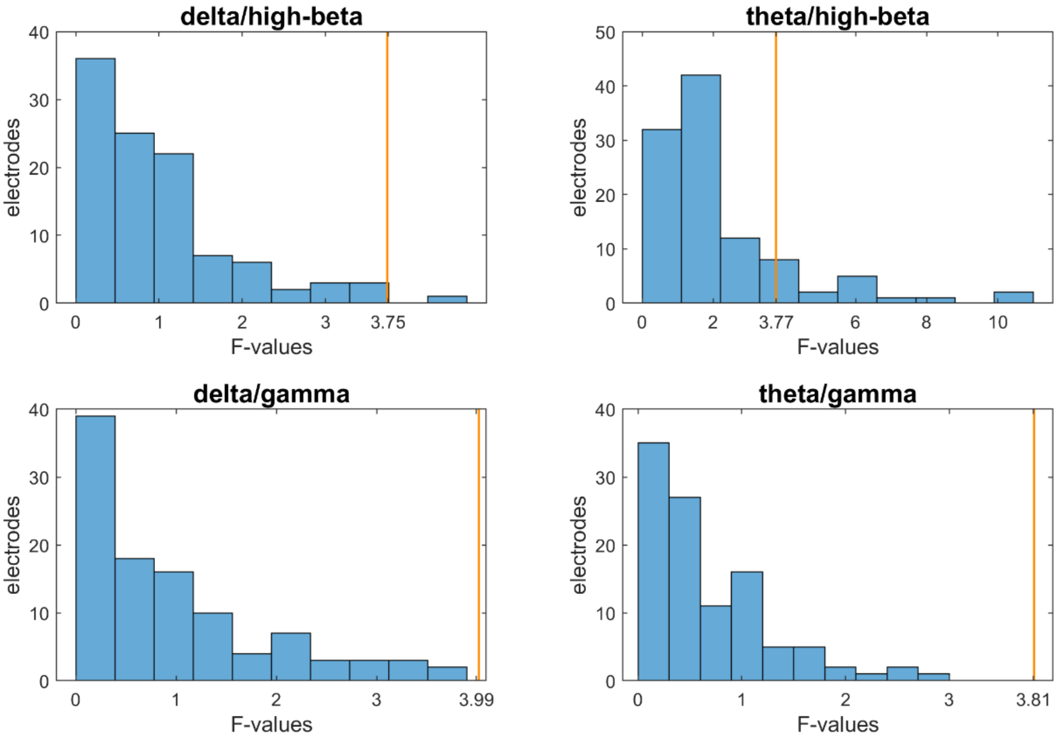

Figure S14. The distributions of F-values related to the electrode-wise Repeated Measures ANOVA are presented for each couple of bands (blue bars). In each plot the F-threshold for significance at $p < 0.05$ (estimated using a single threshold permutation test for the maximum F-statistics, 1000 permutations), is denoted by an orange line.

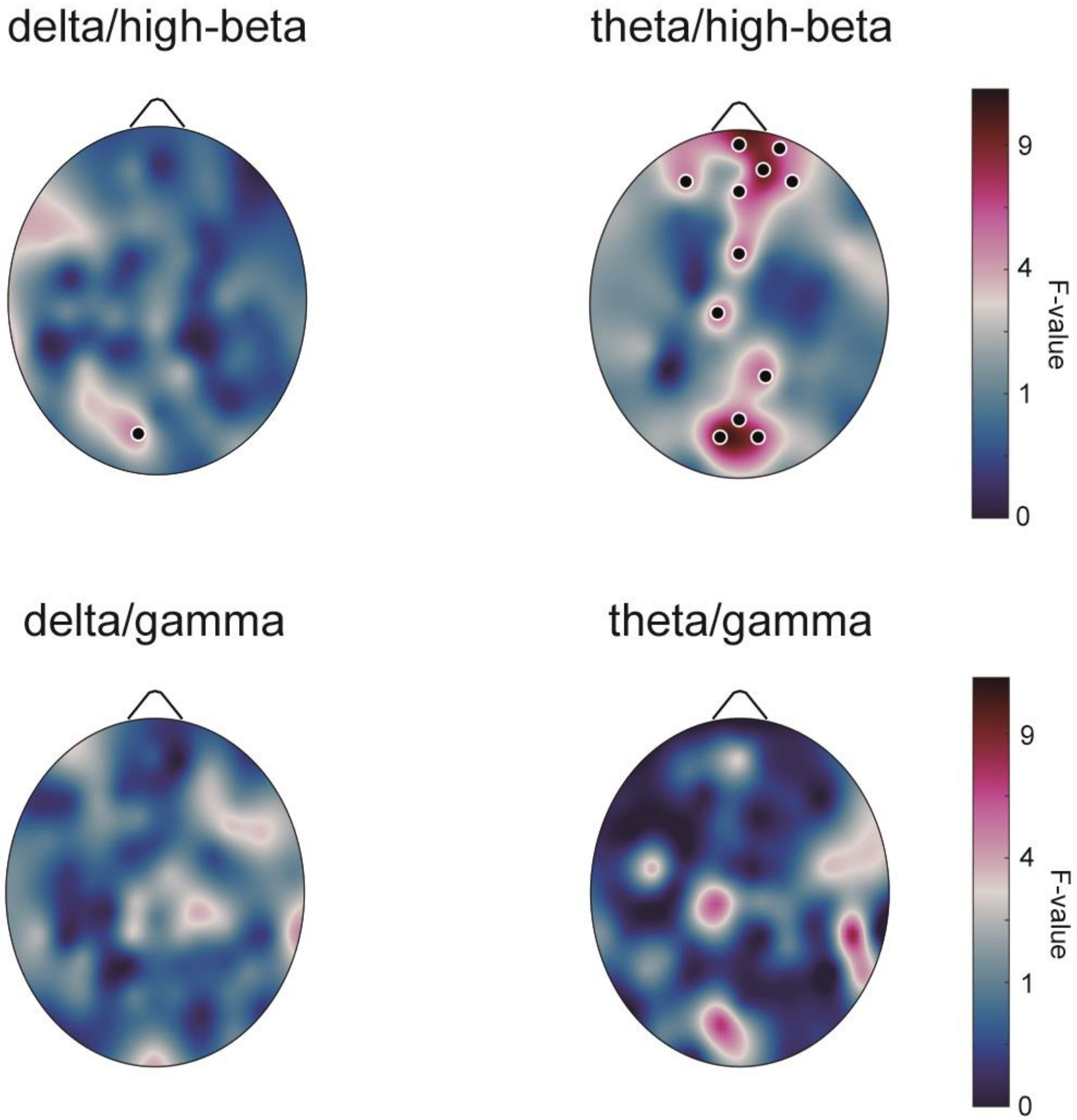

Figure S15. Topographic maps of F-values distributions (within subject *phase*-effect: post-SNB, post-SMB, baseline) are presented for each couple of bands. Black dots indicate electrodes showing a significant *phase*-effect ($p < 0.05$, single threshold permutation test for the maximum F-statistics).

Post-hocs tables

Delta/High-Beta

electrodes	post-SNB vs post-SMB			post-SNB vs baseline			post-SMB vs baseline		
	$\|t_{0.05}\|$	t-value	p_{BH}	$\|t_{0.05}\|$	t-value	p_{BH}	$\|t_{0.05}\|$	t-value	p_{BH}
**E71**	2.12	2.12	0.100	2.14	-0.24	0.813	2.22	-3.16	0.015

Table S20. Results of post-hoc analyses are presented for those electrode showing a significant phase-effect for delta/high-beta coupling. For each electrode and comparison (i.e. post-SNB vs post-SMB, post-SNB vs baseline and post-SMB vs baseline) three statistics are presented: $|t_{0.05}|$ indicates the two-sided significance threshold (at $p < 0.05$) derived by the permutation test on t-statistic (1000 permutations), *t-value*, the t-statistics of the paired t-test, and p_{BH} the test significance after Bonferroni-Holm correction.

Theta/High-Beta

electrodes	post-SNB vs post-SMB			post-SNB vs baseline			post-SMB vs baseline		
	$\|t_{0.05}\|$	t-value	p_{BH}	$\|t_{0.05}\|$	t-value	p_{BH}	$\|t_{0.05}\|$	t-value	p_{BH}
**E6**	2.22	3.15	0.018	2.15	3.28	0.018	2.21	-1.21	0.256
**E9**	2.20	3.28	0.022	2.17	4.00	0.009	2.29	0.55	0.608
**E10**	2.13	4.90	0.001	2.20	3.99	0.008	2.32	0.12	0.889
**E11**	2.33	2.77	0.054	2.18	3.42	0.018	2.31	0.08	0.947
**E15**	2.17	3.50	0.014	2.12	4.36	0.009	2.03	0.97	0.394
**E23**	2.23	2.24	0.096	2.14	3.28	0.001	2.26	0.60	0.571
**E31**	2.22	3.20	0.027	2.16	2.96	0.032	2.16	-0.01	0.991
**E71**	2.02	4.64	0.003	2.17	3.09	0.020	2.28	-1.05	0.309
**E72**	2.19	3.24	0.018	2.20	2.72	0.040	2.06	-0.58	0.586
**E76**	2.17	3.63	0.012	2.13	2.74	0.054	2.25	-1.13	0.287
**E78**	2.19	3.37	0.030	2.11	1.51	0.179	2.10	-1.84	0.162

Table S21. Results of post-hoc analyses are presented for those electrode showing a significant phase-effect for theta/high-beta coupling. For each electrode and comparison (i.e. post-SNB vs post-SMB, post-SNB vs baseline and post-SMB vs baseline) three statistics are presented: $|t_{0.05}|$ indicates the two-sided significance threshold (at $p < 0.05$) derived by the permutation test on t-statistic (1000 permutations), *t-value*, the t-statistics of the paired t-test, and p_{BH} the test significance after Bonferroni-Holm correction.

Please note that in the post-hoc tables, we report only the statistics of those electrodes showing both a significant phase-effect and at least one significant post-hoc comparison.

SM7. Graph Theory Metrics

DELTA METRICS	RM ANOVA		
	$F_{0.05}$	F	p_{FDR}
GRAPH STRENGTH	3.62	3.32	0.11
CLUSTERING COEFFICIENT	3.02	3.69	0.11
GLOBAL EFFICIENCY	3.65	3.82	0.11
MODULARITY	3.06	0.57	0.71
PARTICIPATION COEFFICIENT	3.53	1.05	0.57
MODULAR SPAN	3.16	0.32	0.74

Table S22. Repeated Measures ANOVA statistics for delta metrics are reported (*phase*: baseline, post-SNB, post-SMB, baseline, as a three-levels within factor). $|F_{0.05}|$ indicates the *phase*-effect threshold for significance at $p < 0.05$, derived by the permutation test (1000 permutations), *F-value*, the statistics of the Repeated Measures ANOVA and p_{FDR} the significance after Benjamini-Hochberg correction.

THETA METRICS	RM ANOVA		
	$F_{0.05}$	F	p_{FDR}
GRAPH STRENGTH	6.13	3.20	0.009
CLUSTERING COEFFICIENT	2.88	3.31	0.089
GLOBAL EFFICIENCY	5.35	3.26	0.012
MODULARITY	1.53	3.47	0.238
PARTICIPATION COEFFICIENT	5.09	3.36	0.027
MODULAR SPAN	10.36	3.15	0.006

Table S23a. Repeated Measures ANOVA statistics for theta metrics are reported (*phase*: baseline, post-SNB, post-SMB, baseline, as a three-levels within factor). $|F_{0.05}|$ indicates the *phase*-effect threshold for significance at $p < 0.05$, derived by the permutation test (1000 permutations), *F-value*, the statistics of the Repeated Measures ANOVA and p_{FDR} the significance after Benjamini-Hochberg correction.

THETA METRICS	post-SNB vs post-SMB			post-SNB vs baseline			post-SMB vs baseline		
	$\|t_{0.05}\|$	t-value	p_{BH}	$\|t_{0.05}\|$	t-value	p_{BH}	$\|t_{0.05}\|$	t-value	p_{BH}
GRAPH STRENGTH	2.10	1.94	0.148	2.13	3.45	0.015	2.29	1.45	0.166
GLOBAL EFFICIENCY	2.18	1.65	0.204	2.14	3.20	0.015	2.23	1.74	0.214
PARTICIPATION COEFFICIENT	2.07	-2.40	0.050	2.06	-3.65	0.021	2.19	-0.78	0.446
MODULAR SPAN	2.07	2.74	0.018	2.09	4.18	0.003	2.02	1.94	0.062

Table S23b. Results of post-hoc analyses are presented for those metrics showing a significant phase-effect in theta band. For each metric and comparison (i.e. post-SNB vs post-SMB, post-SNB vs baseline and post-SMB vs baseline) three statistics are presented: $|t_{0.05}|$ indicates the two-sided significance threshold (at $p < 0.05$) derived by the permutation test on t-statistic (1000 permutations), *t-value*, the t-statistics of the paired t-test, and p_{BH} the test significance after Bonferroni-Holm correction.

ALPHA METRICS	RM ANOVA		
	$F_{0.05}$	F	p_{FDR}
**GRAPH STRENGTH**	3.38	1.22	0.630
**CLUSTERING COEFFICIENT**	3.08	1.03	0.630
**GLOBAL EFFICIENCY**	3.19	1.38	0.630
**MODULARITY**	3.42	0.03	0.972
**PARTICIPATION COEFFICIENT**	3.48	0.54	0.713
**MODULAR SPAN**	3.53	0.89	0.630

Table S24. Repeated Measures ANOVA statistics for alpha metrics are reported (*phase*: baseline, post-SNB, post-SMB, baseline, as a three-levels within factor). $|F_{0.05}|$ indicates the *phase*-effect threshold for significance at $p < 0.05$, derived by the permutation test (1000 permutations), *F-value*, the statistics of the Repeated Measures ANOVA and p_{FDR} the significance after Benjamini-Hochberg correction.

LOW-BETA METRICS	RM ANOVA		
	$F_{0.05}$	F	p_{FDR}
**GRAPH STRENGTH**	3.67	2.79	0.176
**CLUSTERING COEFFICIENT**	3.62	2.18	0.185
**GLOBAL EFFICIENCY**	3.73	3.26	0.176
**MODULARITY**	3.51	0.34	0.724
**PARTICIPATION COEFFICIENT**	3.16	2.82	0.176
**MODULAR SPAN**	3.50	1.69	0.253

Table S25. Repeated Measures ANOVA statistics for low-beta metrics are reported (*phase*: baseline, post-SNB, post-SMB, baseline, as a three-levels within factor). $|F_{0.05}|$ indicates the *phase*-effect threshold for significance at $p < 0.05$, derived by the permutation test (1000 permutations), *F-value*, the statistics of the Repeated Measures ANOVA and p_{FDR} the significance after Benjamini-Hochberg correction.

HIGH-BETA METRICS	RM ANOVA		
	$F_{0.05}$	F	p_{FDR}
**GRAPH STRENGTH**	3.32	13.74	0.003
**CLUSTERING COEFFICIENT**	3.57	6.20	0.012
**GLOBAL EFFICIENCY**	3.40	11.98	0.003
**MODULARITY**	3.56	0.61	0.668
**PARTICIPATION COEFFICIENT**	3.56	0.19	0.841
**MODULAR SPAN**	3.40	3.88	0.049

Table S26a. Repeated Measures ANOVA statistics for high-beta metrics are reported (*phase*: baseline, post-SNB, post-SMB, baseline, as a three-levels within factor). $|F_{0.05}|$ indicates the *phase*-effect threshold for significance at $p < 0.05$, derived by the permutation test (1000 permutations), *F-value*, the statistics of the Repeated Measures ANOVA and p_{FDR} the significance after Benjamini-Hochberg correction.

HIGH-BETA METRICS	post-SNB vs post-SMB			post-SNB vs baseline			post-SMB vs baseline		
	$\|t_{0.05}\|$	t-value	p_{BH}	$\|t_{0.05}\|$	t-value	p_{BH}	$\|t_{0.05}\|$	t-value	p_{BH}
**GRAPH STRENGTH**	3.28	2.24	0.016	2.17	4.31	0.003	2.16	2.65	0.021
**CLUSTERING COEFFICIENT**	2.77	2.15	0.024	2.13	3.04	0.024	2.18	1.13	0.272
**GLOBAL EFFICIENCY**	3.05	2.26	0.018	2.19	4.17	0.006	2.14	2.41	0.027
**MODULAR SPAN**	0.01	2.00	0.994	2.17	2.23	0.129	2.26	2.30	0.129

Table S26b. Results of post-hoc analyses are presented for those metrics showing a significant phase-effect in high-beta band. For each metric and comparison (i.e. post-SNB vs post-SMB, post-SNB vs baseline and post-SMB vs baseline) three statistics are presented: $|t_{0.05}|$ indicates the two-sided significance threshold (at $p < 0.05$) derived by the permutation test on t-statistic (1000 permutations), *t-value*, the t-statistics of the paired t-test, and p_{BH} the test significance after Bonferroni-Holm correction.

GAMMA METRICS	RM ANOVA		
	$F_{0.05}$	F	p_{FDR}
**GRAPH STRENGTH**	3.31	0.49	0.586
**CLUSTERING COEFFICIENT**	3.13	2.64	0.240
**GLOBAL EFFICIENCY**	3.11	0.94	0.445
**MODULARITY**	3.41	2.12	0.300
**PARTICIPATION COEFFICIENT**	3.74	5.24	0.096
**MODULAR SPAN**	3.38	1.40	0.445

Table S27. Repeated Measures ANOVA statistics for gamma metrics are reported (*phase*: baseline, post-SNB, post-SMB, baseline, as a three-levels within factor). $|F_{0.05}|$ indicates the *phase*-effect threshold for significance at $p < 0.05$, derived by the permutation test (1000 permutations), *F-value*, the statistics of the Repeated Measures ANOVA and p_{FDR} the significance after Benjamini-Hochberg correction.

Graph metrics as a function of connection density

In the following figures (one for each band), the six chosen graph theory metrics as a function of connection densities are presented for the three phases (dark red: post-SNB, blue: post-SMB and gray: baseline). For each band, metric and phase, the error-bar at each connection density value (0.9 down to 0.1) represents the mean ± standard error of the metric averaged across subjects and condition.

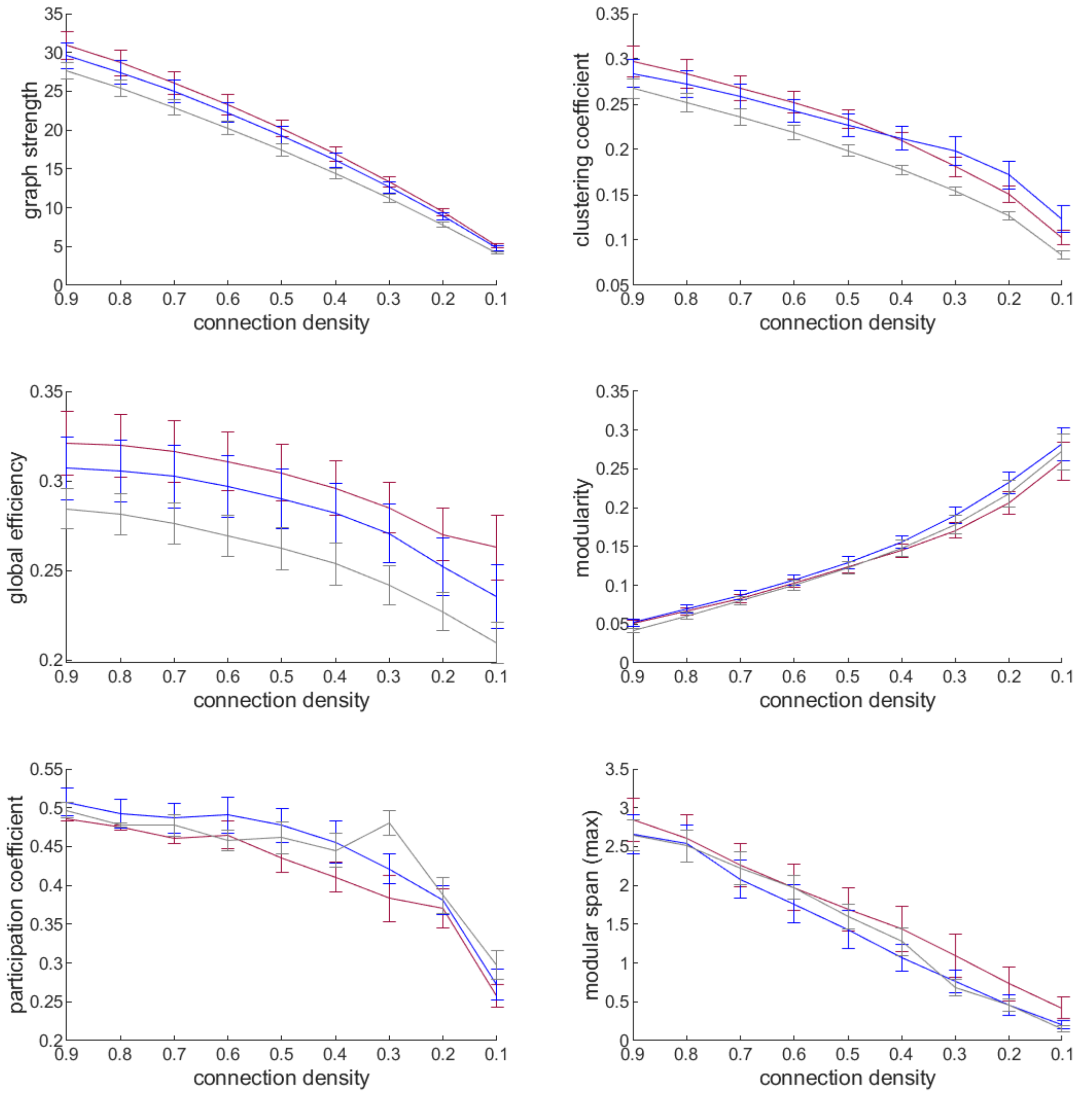

Figure S16. Delta Band

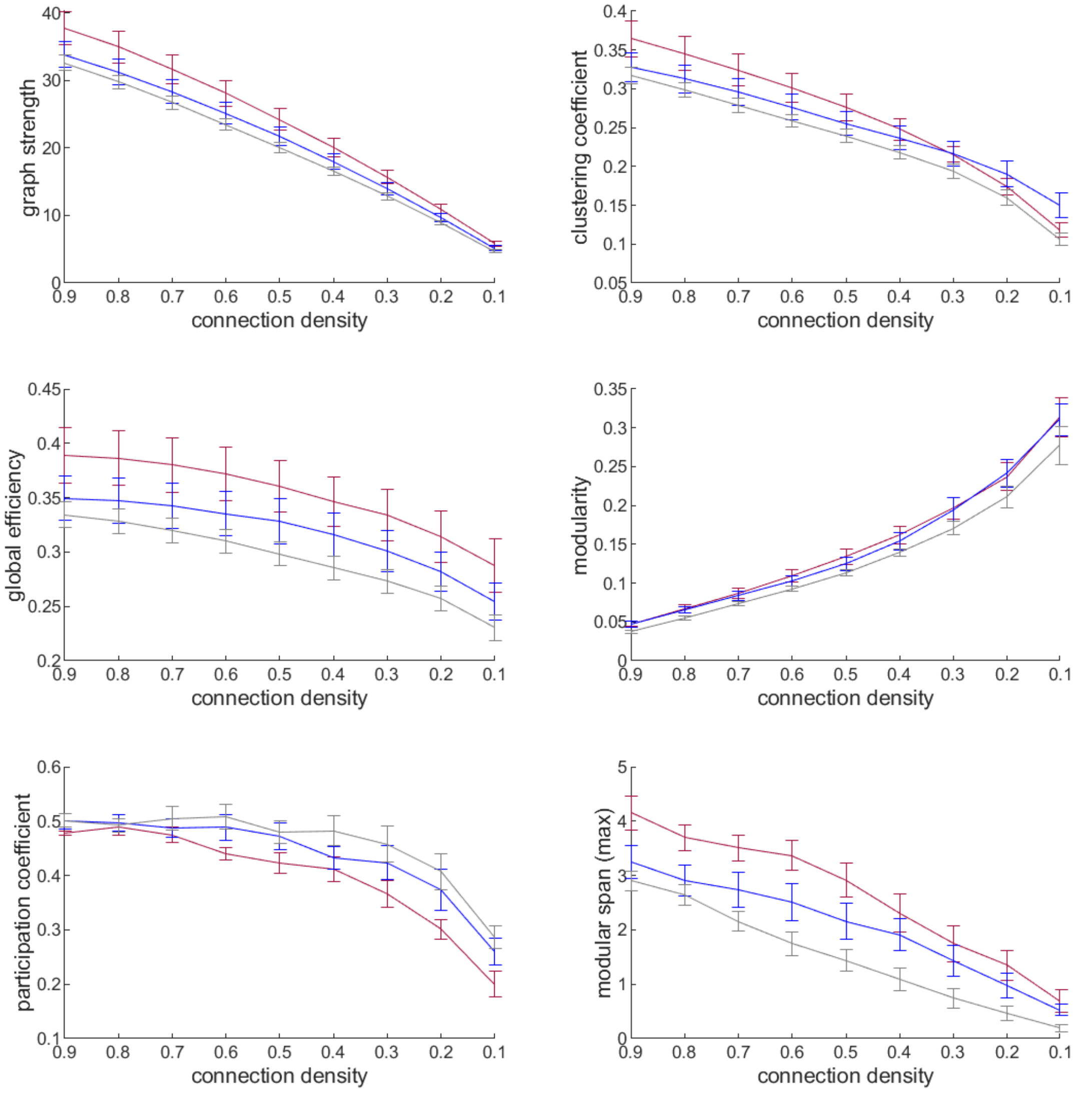

Figure S17. Theta Band

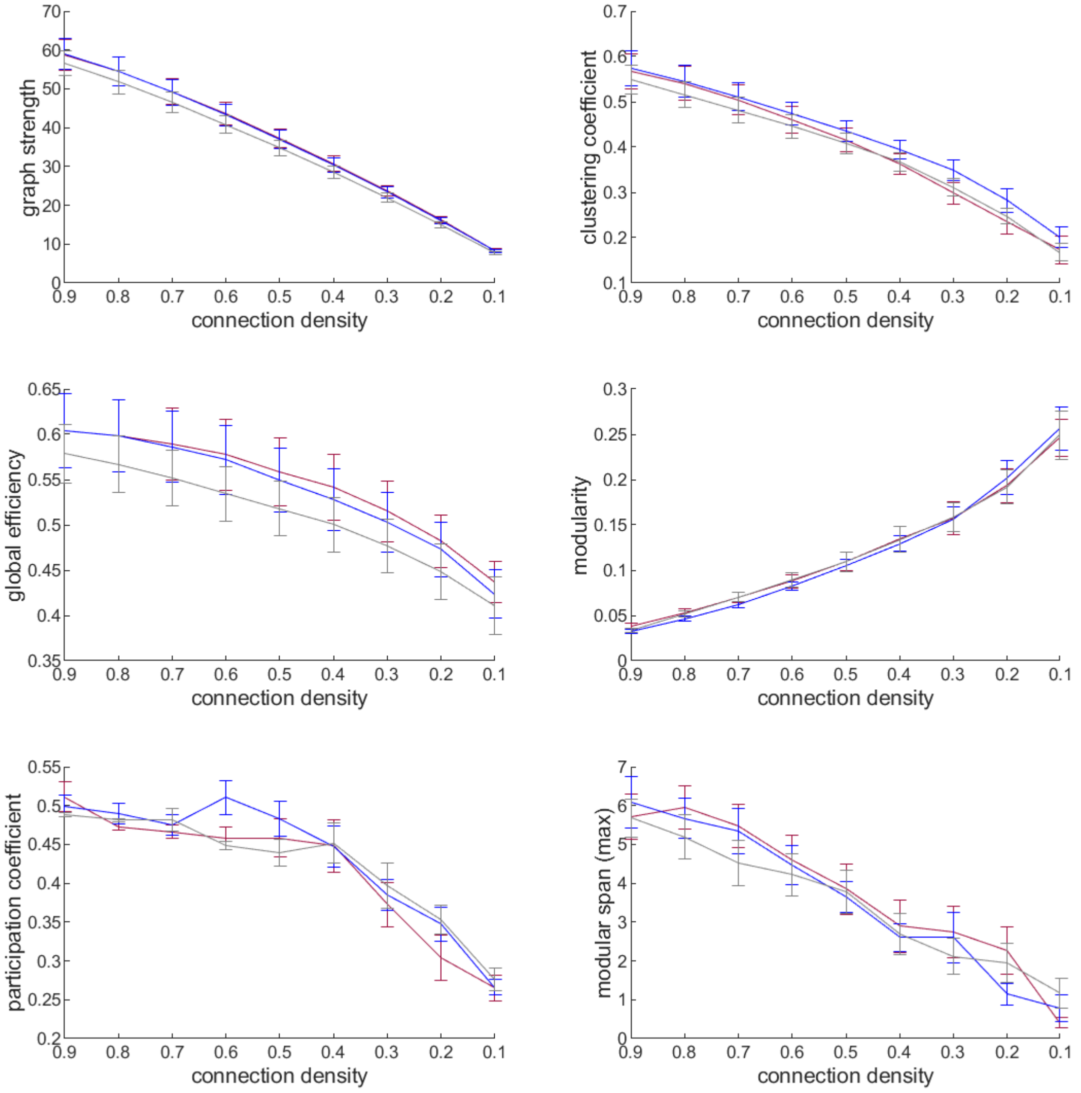

Figure S18. Alpha Band

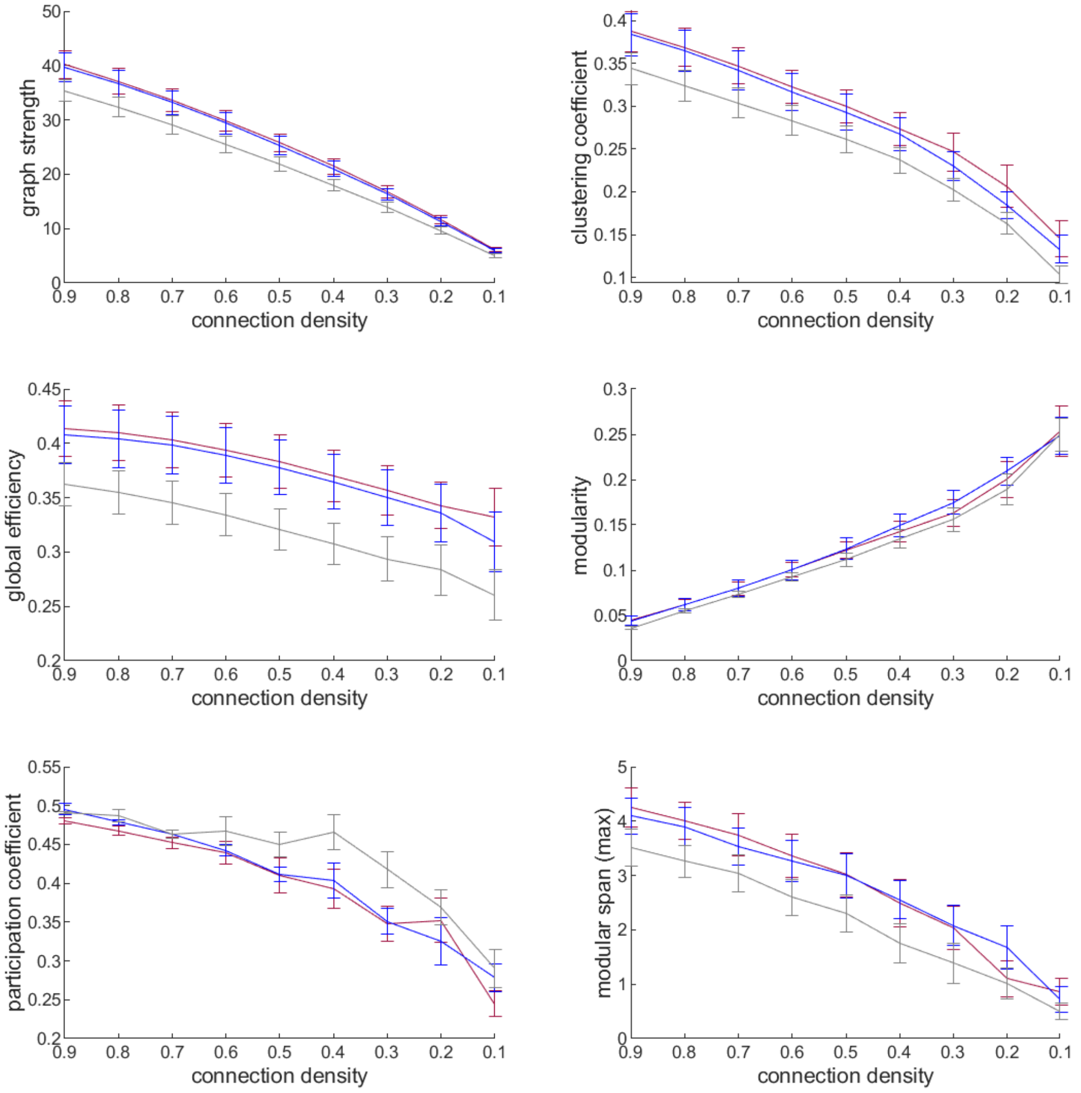

Figure S19. Low-Beta Band

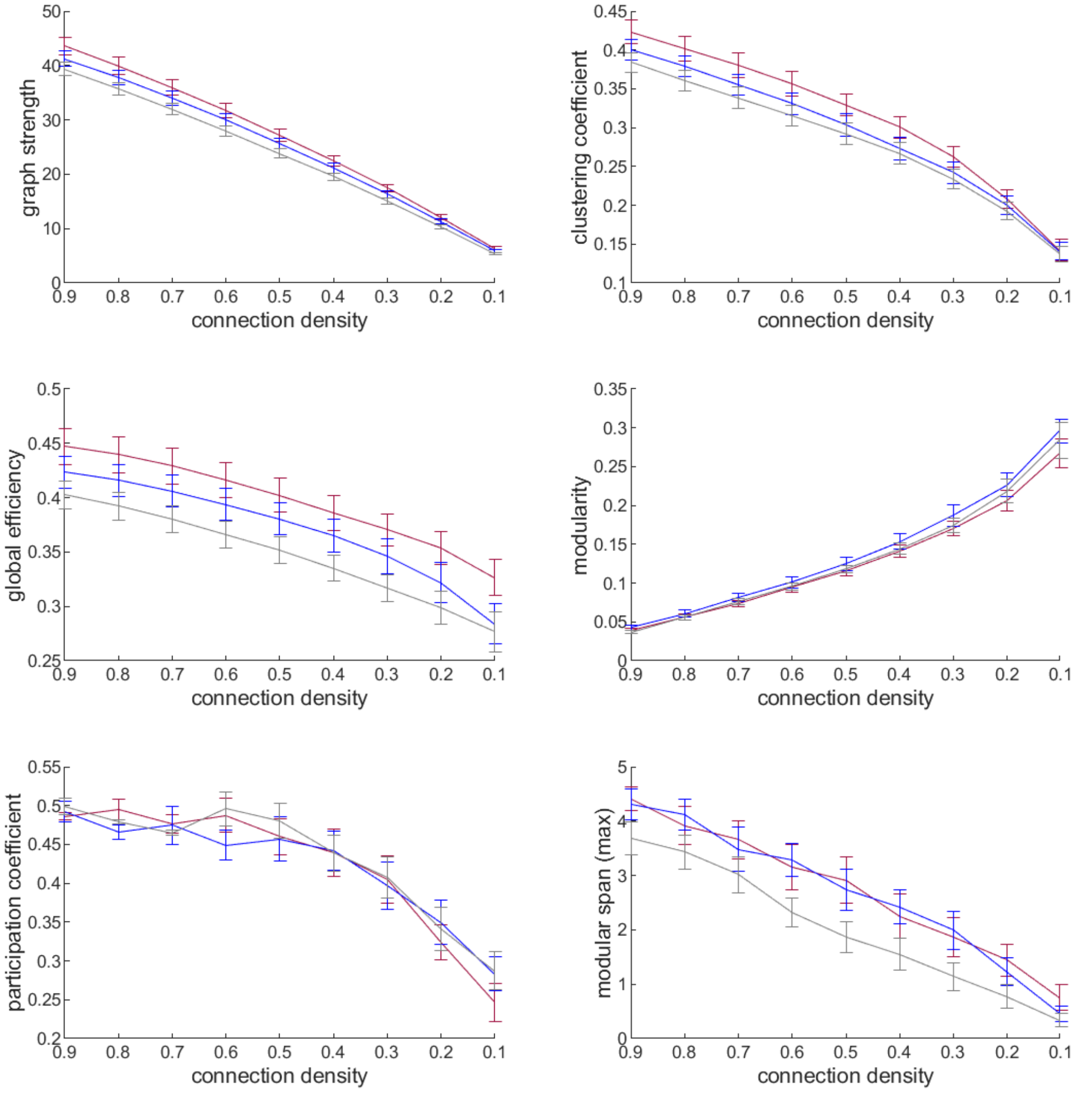

Figure S20. High-Beta Band

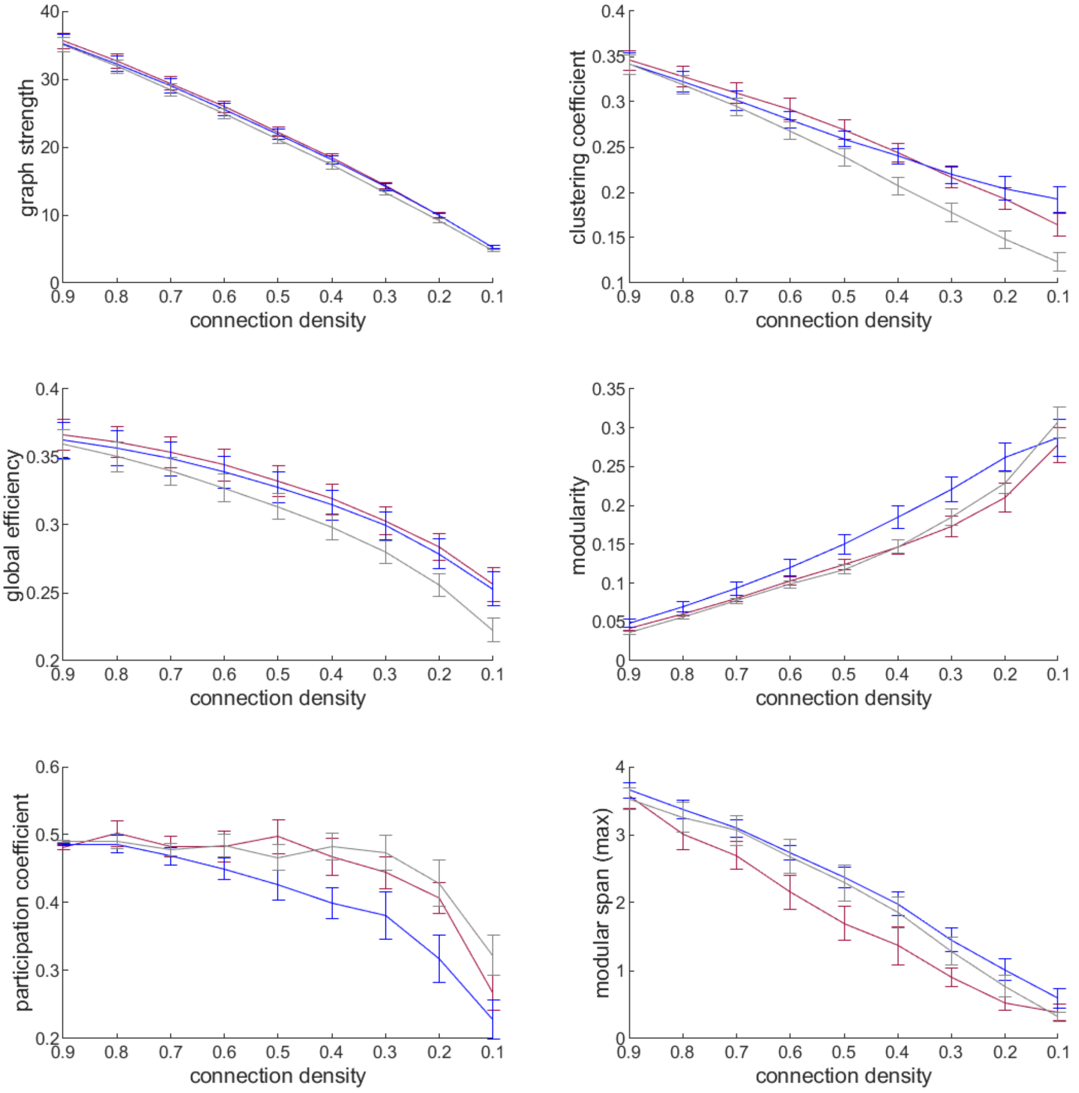

Figure S21. Gamma Band

SM8. Psychometric assessment

Psychometric evaluation

	post-SNB		post-SMB		baseline	
	**mean**	**stde**	**mean**	**stde**	**mean**	**stde**
**positive affect**	2.09	0.39	1.23	0.31	1.60	0.29
joy	2.63	0.42	1.25	0.35	1.58	0.39
sex	1.00	0.49	0.58	0.36	0.63	0.27
love	2.67	0.50	1.83	0.40	2.56	0.38
**negative affect**	0.43	0.18	1.02	0.29	0.53	0.34
anger	0.54	0.29	1.04	0.33	0.44	0.31
sad	0.29	0.17	0.79	0.31	0.67	0.40
fear	0.46	0.26	1.21	0.35	0.48	0.32
**altered experience**	2.55	0.21	1.63	0.29	1.52	0.30
body	3.38	0.31	1.93	0.25	2.10	0.36
time	2.95	0.45	2.51	0.48	1.41	0.38
perception	1.61	0.33	1.07	0.37	1.08	0.29
meaning	2.33	0.37	1.18	0.32	1.46	0.34
**visual imagery**	3.81	0.30	2.85	0.22	3.71	0.31
amount	4.04	0.38	2.58	0.51	3.83	0.51
vividness	3.54	0.40	3.08	0.21	3.54	0.29
**attention**	4.42	0.27	3.98	0.32	3.90	0.22
inward	4.56	0.26	3.98	0.37	3.94	0.25
absorption	4.21	0.42	4.00	0.45	3.83	0.33
**self awareness**	4.22	0.35	4.42	0.36	4.29	0.26
**altered awareness**	3.33	0.41	1.99	0.40	1.84	0.39
**internal dialogue**	2.92	0.48	2.79	0.51	2.13	0.42
**rationality**	4.08	0.47	4.08	0.39	4.26	0.36
**volition**	3.33	0.44	3.81	0.41	3.35	0.35
**memory**	4.23	0.50	4.87	0.28	4.42	0.32
**arousal**	0.83	0.32	2.25	0.43	1.02	0.39
**STAI**	8.73	0.51	10.08	0.48	10.40	0.79

Table S28. Descriptive statistics (mean and standard error) related to the three phases (post-SNB, post-SMB and baseline), are presented for PCI scales and subscales and for STAI scores.

	RM ANOVA		
	**$F_{0.05}$**	**F**	**p_{FDR}**
**PCI**			
**positive affect**	**3.15**	**4.34**	**0.043**
joy	**3.51**	**6.80**	**0.027**
sex	2.93	1.23	0.457
love	3.38	2.53	0.21
**negative affect**	3.02	2.00	0.295
anger	3.13	1.65	0.365
sadness	3.28	1.44	0.374
fear	3.18	1.95	0.329
**altered experience**	**3.03**	**8.04**	**0.027**
body	**3.48**	**6.53**	**0.032**
time	**3.57**	**8.59**	**0.027**
perception	3.33	2.05	0.322
meaning	**3.04**	**5.49**	**0.036**
**visual imagery**	**3.45**	**5.72**	**0.043**
amount	3.50	3.18	0.162
vividness	3.54	0.97	0.457
attention	3.13	1.29	0.413
inward	3.39	1.46	0.374
absorption	3.20	0.27	0.826
**self awareness**	3.41	0.17	0.867
**altered awareness**	**3.56**	**5.66**	**0.039**
**internal dialogue**	3.20	1.62	0.341
**rationality**	3.15	0.11	0.88
**volition**	3.08	0.55	0.67
**memory**	3.52	1.27	0.415
**arousal**	**3.19**	**4.82**	**0.043**
**STAI**	**3.23**	**5.81**	**0.027**

Table S29. Repeated Measures ANOVA statistics for PCI scales and sub-scale and STAI scores are reported (*phase*: post-SNB, post-SMB, baseline, as a three-levels within factor). $|F_{0.05}|$ indicates the *phase*-effect threshold for significance at $p < 0.05$, derived by the permutation test on the F-value (1000 permutations), F the statistics of the Repeated Measures ANOVA (phase-effect) and p_{FDR} the significance after Benjamini-Hochberg correction.

	post-SNB vs post-SMB			post-SNB vs baseline			post-SMB vs baseline		
	**|t|0.05**	**t-value**	**pBH**	**|t|0.05**	**t-value**	**pBH**	**|t|0.05**	**t-value**	**pBH**
**PCI- positive affect**	**2.30**	**2.97**	**0.033**	2.09	1.45	0.324	2.183	-1.528	0.324
joy	**2.28**	**3.36**	**0.006**	**2.13**	**2.51**	**0.042**	2.293	-0.983	0.37
**PCI- altered experience**	**2.00**	**3.00**	**0.021**	**2.11**	**3.32**	**0.021**	2.157	0.529	0.611
body	**2.16**	**3.28**	**0.012**	**2.20**	**2.54**	**0.05**	2.129	-0.478	0.635
time	2.22	1.17	0.245	**2.16**	**3.40**	**0.018**	**2.299**	**3.636**	**0.015**
meaning	**2.01**	**2.72**	**0.018**	**1.99**	**2.17**	**0.044**	2.133	-1.188	0.284
**PCI - visual imagery**	**2.21**	**3.06**	**0.042**	2.08	0.36	0.712	2.192	-2.05	0.07
**PCI - altered awareness**	**2.10**	**2.40**	**0.046**	**2.20**	**3.95**	**0.009**	2.189	0.295	0.773
**PCI - Arousal**	2.21	-2.55	0.059	2.10	-1.66	0.312	2.065	1.996	0.122
**STAI-Y**	**2.06**	**-2.63**	**0.042**	**2.107**	**-2.776**	**0.042**	2.190	-0.732	0.503

Table S30. Results of post-hoc analyses are presented for those psychometric scales and sub-scales showing a significant phase-effect. For each psychometric parameter and comparison (i.e. post-SNB vs post-SMB, post-SNB vs baseline and post-SMB vs baseline) three statistics are presented: $|t_{0.05}|$ indicates the two-sided significance threshold (at $p < 0.05$) derived by the permutation test on t-statistic (1000 permutations), *t-value*, the t-statistics of the paired t-test, and p_{BH} the test significance after Bonferroni-Holm correction.
